# Penetrance of Neurodevelopmental Copy Number Variants Is Associated With Variations in Cortical Morphology

**DOI:** 10.1016/j.bpsc.2025.05.010

**Published:** 2025-05-23

**Authors:** Ana I. Silva, Ida E. Sønderby, George Kirov, Abdel Abdellaoui, Ingrid Agartz, David Ames, Nicola J. Armstrong, Eric Artiges, Tobias Banaschewski, Anne S. Bassett, Carrie E. Bearden, John Blangero, Rune Boen, Dorret I. Boomsma, Robin Bülow, Nancy J. Butcher, Vince Calhoun, Linda E. Campbell, Eva W.C. Chow, Simone Ciufolini, Michael C. Craig, Benedicto Crespo-Farroco, Adam C. Cunningham, Shareefa Dalvie, Eileen Daly, Paola Dazzan, Eco J.C. de Geus, Greig I. de Zubicaray, Joanne L. Doherty, Gary Donohoe, Mark Drakesmith, Thomas Espeseth, Vincent Frouin, Hugh Garavan, David C. Glahn, Naomi J. Goodrich-Hunsaker, Penny A. Gowland, Hans J. Grabe, Antoine Grigis, Maria Gudbrandsen, Boris A. Gutman, Jan Haavik, Asta K. Håberg, Jeremy Hall, Andreas Heinz, Sarah Hohmann, Jouke-Jan Hottenga, Sébastien Jacquemont, Neda Jahanshad, Rachel K. Jonas, Derek K. Jones, Erik G. Jönsson, Sanne Koops, Kuldeep Kumar, Stephanie Le Hellard, Herve Lemaitre, Jingyu Liu, Astri J. Lundervold, Jean-Luc Martinot, Karen A. Mather, Donna M. McDonald-McGinn, Katie L. McMahon, Allan F. McRae, Sarah E. Medland, Clara A. Moreau, Kieran C. Murphy, Declan Murphy, Robin M. Murray, Frauke Nees, Michael J. Owen, Marie-Laure Paillère Martinot, Diimitri Papadopoulos Orfanos, Tomas Paus, Luise Poustka, Tiago Reis Marques, David R. Roalf, Perminder S. Sachdev, Freda Scheffler, J. Eric Schmitt, Gunter Schumann, Vidar M. Steen, Dan J. Stein, Lachlan T. Strike, Alexander Teumer, Anbupalam Thalamuthu, Sophia I. Thomopoulos, Diana Tordesillas-Gutiérrez, Julian N. Trollor, Anne Uhlmann, Ariana Vajdi, Dennis van ’t Ent, Therese van Amelsvoort, Marianne B.M. van den Bree, Dennis van der Meer, Javier Vázquez-Bourgon, Julio E. Villalón-Reina, Uwe Völker, Henry Völzke, Jacob A.S. Vorstman, Lars T. Westlye, Nigel Williams, Katharina Wittfeld, Margaret J. Wright, Paul M. Thompson, Ole A. Andreassen, David E.J. Linden

**Affiliations:** School for Mental Health and Neuroscience, Department of Psychiatry and Neuropsychology, Faculty of Health, Medicine and Life Sciences, Maastricht University, Maastricht, the Netherlands (AIS, TvA, DvdM, DEJL); Neuroscience and Mental Health Innovation Institute, Cardiff University, Cardiff, United Kingdom (AIS, GK, JHal, MJO, MBMvdB); Center for Magnetic Resonance Research, Department of Radiology, University of Minnesota, Minneapolis, Minnesota (AIS); Centre for Precision Psychiatry, Institute of Clinical Medicine, University of Oslo, Oslo, Norway (IES, EGJ, DvdM, LTW, OAA); Department of Medical Genetics, Oslo University Hospital, Oslo, Norway (IES); KG Jebsen Centre for Neurodevelopmental Disorders, University of Oslo, Oslo, Norway (IES, LTW); Division of Psychological Medicine and Clinical Neurosciences, Cardiff University, Cardiff, United Kingdom (GK, ACC, JLD, JHal, MJO, MBMvdB, NW); Centre for Neuropsychiatric Genetics and Genomics, School of Medicine, Cardiff University, Cardiff, United Kingdom (GK, ACC, JLD, JHal, MJO, MBMvdB, NW); Department of Psychiatry, Amsterdam UMC, University of Amsterdam, Amsterdam, the Netherlands (AA); Norwegian Centre for Mental Disorders Research (NORMENT), Institute of Clinical Medicine, University of Oslo, Oslo, Norway (IA); Centre for Psychiatry Research, Department of Clinical Neuroscience, Karolinska Institutet, Stockholm & Stockholm Health Care Services, Stockholm Region, Stockholm, Sweden (IA, EGJ); Department of Psychiatric Research, Diakonhjemmet Hospital, Oslo, Norway (IA); University of Melbourne Academic Unit for Psychiatry of Old Age, Kew, Victoria, Australia (DA); Mathematics and Statistics, Curtin University, Perth, Western Australia, Australia (NJA); Institut National de la Santé et de la Recherche Médicale (INSERM) U1299 “Trajectoires développementales et psychiatrie,” Université Paris-Saclay, Centre National de la Recherche Scientifique, Centre Borelli, Gif-sur-Yvette, France (EA, J-LM, M-LPM); Psychiatry Department, Établissement public de santé Barthélémy Durand, Étampes, Île-de-France, France (EA); Department of Child and Adolescent Psychiatry and Psychotherapy, Central Institute of Mental Health, Medical Faculty Mannheim, University of Heidelberg, Mannheim, Germany (TB, SH); Clinical Genetics Research Program, Centre for Addiction and Mental Health, Toronto, Ontario, Canada (ASB, EWCC); Department of Psychiatry, University of Toronto, Toronto, Ontario, Canada (ASB, NJB, EWCC); Dalglish Family 22q Clinic for Adults with 22q11.2 Deletion Syndrome, Toronto General Hospital, University Health Network, Toronto, Ontario, Canada (ASB); Semel Institute for Neuroscience and Human Behavior, University of California, Los Angeles, California (CEB, RBo, RKJ, AV); Department of Psychology, University of California, Los Angeles, California (CEB); Department of Human Genetics and South Texas Diabetes and Obesity Institute, University of Texas Rio Grande Valley, Brownsville, Texas (JB); Complex Trait Genetics, Center for Neurogenomics and Cognitive Research, Vrije Universiteit Amsterdam, Amsterdam, the Netherlands (DIB); Amsterdam Public Health Research Institute, Amsterdam UMC, Amsterdam, the Netherlands (DIB, EJCdG, Dv’E); Institute for Diagnostic Radiology and Neuroradiology, University Medicine Greifswald, Greifswald, Germany (RBü); Child Health Evaluative Sciences, The Hospital for Sick Children Research Institute, Toronto, Ontario, Canada (NJB); Tri-institutional Center for Translational Research in Neuroimaging and Data Science, Georgia State, Georgia Tech, Emory University, Atlanta, Georgia (VC, JL); School of Psychological Science, University of Newcastle, Newcastle, New South Wales, Australia (LEC); Department of Psychosis Studies, Institute of Psychiatry, Psychology and Neuroscience, King’s College London, London, United Kingdom (SC, RMM, TRM); National Female Hormone Clinic, London, United Kingdom (MCC); Department of Forensic and Neurodevelopmental Science, Institute of Psychiatry, Psychology and Neuroscience, King’s College London, London, United Kingdom (MCC, ED, MG, DM); Centro de Investigación Biomédica en Red en Salud Mental, Sevilla, Spain (BC-F, JV-B); Instituto de Investigación Biomédica de Sevilla IBIS-CSIC, Hospital Universitario Virgen del Rocío, Universidad de Sevilla, Departamento de Psiquiatría, Sevilla, Spain (BC-F); Biomedical Research and Innovation Platform, South African Medical Research Council, Cape Town, South Africa (SD); Department of Psychiatry and Neuroscience Institute, University of Cape Town, Cape Town, South Africa (SD); Department of Psychological Medicine, Institute of Psychiatry, Psychology and Neuroscience, King’s College London, London, United Kingdom (PD); Department of Biological Psychology, Faculty of Behavioral and Movement Sciences, Vrije Universiteit Amsterdam, Amsterdam, the Netherlands (EJCdG, J-JH, Dv’E); School of Psychology and Counselling, Faculty of Health, Queensland University of Technology, Brisbane, Queensland, Australia (GIdZ); Cardiff University’s Brain Research Imaging Centre, School of Psychology, Cardiff University, Cardiff, United Kingdom (JLD, MD, DKJ); School of Psychology, University of Galway, Galway, Ireland (GD); Center for Neuroimaging, Cognition and Genomics, University of Galway, Galway, Ireland (GD); Communicable Disease Surveillance Centre, Public Health Wales, Cardiff, Wales, United Kingdom (MD); Department of Psychology, University of Oslo, Oslo, Norway (TE, LTW); Department of Psychology, Oslo New University College, Oslo, Norway (TE); Université Paris-Saclay, French Alternative Energies and Atomic Energy Commission, Neurospin, Gif-sur-Yvette, France (VF, AG, DPO); Department of Psychological Science, University of Vermont, Burlington, Vermont (HG); Department of Psychiatry and Behavioral Sciences, Boston Children’s Hospital, Boston, Massachusetts (DCG); Department of Psychiatry, Harvard Medical School, Boston, Massachusetts (DCG); Department of Neurology, The University of Utah, Salt Lake City, Utah (NJG-H); Sir Peter Mansfield Imaging Centre, University of Nottingham, Nottingham, United Kingdom (PAG); Department of Psychiatry and Psychotherapy, University Medicine Greifswald, Greifswald, Germany (HJG, ATe, KW); Centre for Research in Psychological Wellbeing, School of Psychology, University of Roehampton, London, United Kingdom (MG); Department of Biomedical Engineering, Illinois Institute of Technology, Chicago, Illinois (BAG); Department of Biomedicine, University of Bergen, Bergen, Norway (JHaa); Division of Psychiatry, Haukeland University Hospital, Bergen, Norway (JHaa); Department of Neuromedicine and Movement Science, Norwegian University of Science and Technology, Trondheim, Norway (AKH); MiDT National Research Center, Centre for Medical Equipment, Technology, and Innovation, St. Olav’s Hospital, Trondheim, Norway (AKH); Department of Psychiatry and Psychotherapy, Charité Universitätsmedizin Berlin, corporate member of Freie Universität Berlin and Humboldt-Universität zu Berlin, Berlin, Germany (AH); German Center for Mental Health, Partner Site Berlin-Potsdam, Berlin, Germany (AH); Department of Child and Adolescent Psychiatry and Psychotherapy, University Hospital Hamburg-Eppendorf, Hamburg, Germany (SH); Neurological Disorder Research Center, Qatar Biomedical Research Institute, Hamad Bin Khalifa University, Qatar Foundation, Doha, Qatar (J-JH); Centre de recherche CHU Sainte Justine, Department of Psychiatry and Addictology, University of Montreal, Montreal, Quebec, Canada (SJ, KK, CAM); Department of Pediatrics, University of Montreal, Montreal, Quebec, Canada (SJ); Imaging Genetics Center, Mark and Mary Stevens Neuroimaging and Informatics Institute, Keck School of Medicine of the University of Southern California, Marina del Rey, California (NJ, SIT, JEV-R, PMT); Biomedical Sciences of Cells and Systems, University Medical Center Groningen, Rijksuniversiteit Groningen, Groningen, the Netherlands (SK); NORMENT, Department of Clinical Science, University of Bergen, Bergen, Norway (SLH, VMS); Dr. Einar Martens Research Group for Biological Psychiatry, Department of Medical Genetics, Haukeland University Hospital, Bergen, Norway (SLH, VMS); Groupe d’Imagerie Neurofonctionnelle, Institut des Maladies Neurodégénératives, Centre National de la Recherche Scientifique UMR 5293, Université de Bordeaux, Centre Broca Nouvelle-Aquitaine, Bordeaux, France (HL); Department of Computer Science, Georgia State University, Atlanta, Georgia (JL); Department of Biological and Medical Psychology, University of Bergen, Bergen, Norway (AJL); Centre for Healthy Brain Ageing, Discipline of Psychiatry and Mental Health, School of Clinical Medicine, Faculty of Medicine and Health, University of New South Wales, Sydney, New South Wales, Australia (KAM, PSS, ATh, JNT); Neuroscience Research Australia, Sydney, New South Wales, Australia (KAM); Department of Pediatrics, Perelman School of Medicine of the University of Pennsylvania, Philadelphia, Pennsylvania (DMM-M); Division of Human Genetics, 22q and You Center, Clinical Genetics Center, Section of Genetic Counseling, Children’s Hospital of Philadelphia, Philadelphia, Pennsylvania (DMM-M); Department of Human Biology and Medical Genetics, Sapienza University, Rome, Italy (DMM-M); School of Clinical Sciences, Queensland University of Technology, Brisbane, Queensland, Australia (KLM); Institute for Molecular Bioscience, The University of Queensland, Brisbane, Queensland, Australia (AFM); Psychiatric Genetics, QIMR Berghofer Medical Research Institute, Brisbane, Queensland, Australia (SEM, LTS); Department of Psychiatry, Royal College of Surgeons in Ireland, Dublin, Ireland (KCM); Institute of Medical Psychology and Medical Sociology, University Medical Center Schleswig-Holstein, Kiel University, Kiel, Germany (FN); Assistance Publique-Hôpitaux de Paris, Sorbonne Université, Child and Adolescent Psychiatry Department, Pitiésalpêtrière Hospital, Paris, France (M-LPM); Departments of Psychiatry and Neuroscience, Faculty of Medicine and Centre Hospitalier, Universitaire Sainte-Justine, University of Montreal, Montreal, Quebec, Canada (TP); Department of Psychiatry, McGill University, Montreal, Quebec, Canada (TP); Department of Child and Adolescent Psychiatry and Psychotherapy, University Medical Center Göttingen, Göttingen, Germany (LP); Psychiatric Imaging Group, MRC London Institute of Medical Sciences, Hammersmith Hospital, Imperial College London, London, United Kingdom (TRM); Neurodevelopment and Psychosis Section, Department of Psychiatry, University of Pennsylvania, Philadelphia, Pennsylvania (DRR, JES); Neuropsychiatric Institute, Prince of Wales Hospital, Sydney, New South Wales, Australia (PSS); Department of Psychiatry and Mental Health, University of Cape Town, Cape Town, Western Cape, South Africa (FS); Neuroscience Institute, University of Cape Town, Cape Town, Western Cape, South Africa (FS); Division of Neuroradiology, Department of Radiology, University of Pennsylvania, Philadelphia, Pennsylvania (JES); Centre for Population Neuroscience and Stratified Medicine, Institute of Science and Technology for Brain-inspired Intelligence, Fudan University, Shanghai, Shanghai, China (GS); Centre for Population Neuroscience and Stratified Medicine, Department of Psychiatry and Clinical Neuroscience, Charite University Medicine, Berlin, Germany (GS); South African Medical Research Council Unit on Risk and Resilience, Department of Psychiatry and Neuroscience Institute, University of Cape Town, Cape Town, South Africa (DJS); German Centre for Cardiovascular Research, Partner Site Greifswald, Greifswald, Germany (ATe, UV); Instituto de Física de Cantabria, Santander, Spain (DT-G); Department of Radiology, Instituto de Investigación Valdecilla, Marqués de Valdecilla University Hospital, Santander, Spain (DT-G); National Centre of Excellence in Intellectual Disability Health, Faculty of Medicine and Health, University of New South Wales, Sydney, New South Wales, Australia (JNT); Department of Child and Adolescent Psychiatry and Psychotherapy, TU Dresden, Dresden, Germany (AU); Department of Psychiatry, Charles R. Drew University of Medicine and Science, Los Angeles, California (AV); Department of Psychiatry, Instituto de Investigación Valdecilla, University Hospital Marqués de Valdecilla, Santander, Spain (JV-B); Departamento de Medicina y Psiquiatría, Universidad de Cantabria, Santander, Spain (JV-B); Interfaculty Institute for Genetics and Functional Genomics, University Medicine Greifswald, Greifswald, Germany (UV); Institute for Community Medicine, University Medicine Greifswald, Greifswald, Germany (HV); Department of Psychiatry, The Hospital for Sick Children, University of Toronto, Toronto, Ontario, Canada (JASV); Genetics and Genome Biology, SickKids Research Institute, Toronto, Ontario, Canada (JASV); Queensland Brain Institute, The University of Queensland, Brisbane, Queensland, Australia (MJW); Section for Precision Psychiatry, Oslo University Hospital, Oslo, Norway (OAA).

## Abstract

**BACKGROUND:**

Copy number variants (CNVs) may increase the risk for neurodevelopmental conditions. The neurobiological mechanisms that link these high-risk genetic variants to clinical phenotypes are largely unknown. An important question is whether brain abnormalities in individuals who carry CNVs are associated with their degree of penetrance.

**METHODS:**

We investigated whether increased CNV penetrance for schizophrenia and other developmental disorders was associated with variations in cortical and subcortical morphology. We pooled T1-weighted brain magnetic resonance imaging and genetic data from 22 cohorts from the ENIGMA (Enhancing Neuro Imaging Genetics through Meta Analysis)-CNV consortium. In the main analyses, we included 9268 individuals (aged 7–90 years, 54% female), from which we identified 398 carriers of 36 neurodevelopmental CNVs at 20 distinct loci. A secondary analysis was performed including additional neuroimaging data from the ENIGMA-22q consortium, including 274 carriers of the 22q11.2 deletion and 291 noncarriers. CNV penetrance was estimated through penetrance scores that were previously generated from large cohorts of patients and controls. These scores represent the probability risk of developing either schizophrenia or other developmental disorders (including developmental delay, autism spectrum disorder, and congenital malformations).

**RESULTS:**

For both schizophrenia and developmental disorders, increased penetrance scores were associated with lower surface area in the cerebral cortex and lower intracranial volume. For both conditions, associations between CNV-penetrance scores and cortical surface area were strongest in regions of the occipital lobes, specifically in the cuneus and lingual gyrus.

**CONCLUSIONS:**

Our findings link global and regional cortical morphometric features with CNV penetrance, providing new insights into neurobiological mechanisms of genetic risk for schizophrenia and other developmental disorders.

Copy number variants (CNVs) are structural variations in the genome that involve deletions or duplications of over 1000 base pairs of DNA. Several rare recurrent CNVs have been proposed as pathogenic, leading to genomic disorders and increased risk for neurodevelopmental disorders (NDDs) (1,2) such as schizophrenia, autism spectrum disorder (ASD), and developmental delay (DD) (3). Different CNVs can lead to similar clinical conditions, although with variable penetrance. For example, 22q11.2 deletions are among the strongest genetic risk factors for schizophrenia (odds ratio > 28), whereas 15q11.2 breakpoint (BP)1-BP2 deletions impart low-level risk (odds ratio = 1.3–2.2) (2,4). Genetic studies suggest that distinct CNVs are likely to converge in the path from genome to clinical phenotypes (2,5–8), leading to a degree of similar cognitive and anatomical brain effects across CNVs. Relatively large studies, which have compared wide-ranging phenotypic manifestations across a number of different CNVs, have supported this hypothesis by showing similar effects across traits (9,10).

Several magnetic resonance imaging (MRI) studies have informed on how CNVs at 22q11.2, 1q21.1 distal, 7q11.23, 16p11.2 (BP2-BP3 and BP4-BP5), and 15q11.2 BP1-BP2 loci affect brain macro- and microstructure (8,11–18). The majority of these CNVs were shown to impact global brain morphology, with variable regional effects. ENIGMA (Enhancing Neuro Imaging Genetics through Meta Analysis)-CNV and ENIGMA-22q consortia have published studies on cortical and subcortical alterations in 22q11.2 (16,17), 16p11.2 BP2-BP3 (13), 1q21.1 distal (12), and 15q11.2 (14) CNV carriers compared with noncarrier controls. Modenato *et al*. summarized cortical and subcortical findings from 76 studies on 20 pathogenic CNVs in a systematic review (11). Carriers of 15q11.2 BP1-BP2, 1q21.1 distal, 22q11.2, and 7q11.23 deletions and 16p11.2 BP4-BP5 duplications show similar effects on global measures (lower surface area and lower total brain volume), whereas 16p11.2 BP4-BP5 deletion carriers show opposite effects (higher surface area and higher total brain volume). Effects on global cortical thickness were more variable, with 15q11.2 BP1-BP2, 22q11.2, and 7q11.23 deletions showing higher and 16p11.2 BP4-BP5 duplications showing lower cortical thickness. Emerging studies have looked at both convergent and CNV-specific effects (19–22). A study that combined neuroimaging data from 8 neuropsychiatric CNVs highlighted similar effects on regional volumes across CNV carriers when compared with noncarrier controls—particularly in the cingulate gyrus, insula, supplementary motor cortex, and cerebellum—but the largest proportion of effects was distinct across CNVs (20). However, it is unclear whether specific brain features in individuals who carry CNVs are associated with increased disease risk.

A previous multimodal neuroimaging study investigated how penetrance of each CNV for schizophrenia and other developmental disorders was correlated with brain features in 21 adult participants carrying CNVs with variable penetrance (23). Penetrance scores were used as a measure of CNV penetrance, reflecting the probability of developing either schizophrenia or other developmental disorders (including DD, ASD, and congenital malformations [CMs]) given the presence of a certain CNV. These scores were previously calculated for each CNV by Kirov *et al*. (24) using large patient cohorts from previous studies. Higher CNV penetrance for either schizophrenia or developmental disorders was associated with changes in the curvature of the cingulum and with volumetric interrelationships between segments of the corpus callosum (23). No associations were found between gray matter features and penetrance scores, possibly because the small sample size affected the statistical power.

In this study, we used a much larger neuroimaging dataset (*N* = 9268) from the ENIGMA-CNV consortium, including 398 carriers of 36 CNVs with potential risk for NDDs. We utilized previously estimated CNV-penetrance scores for schizophrenia and developmental disorders (including DD/ASD/CMs) from Kirov *et al*. (24) and updated these scores with control frequency from UK Biobank data (25). We analyzed associations between CNV penetrance and subcortical volumes, intracranial volume (ICV), as well as global and regional surface area and thickness measures of the cerebral cortex. We aimed to identify brain features that are related to neurodevelopmental disease risk across multiple CNVs. This is a key question both mechanistically and clinically because brain mechanisms that are most related to pathogenicity may represent relevant treatment targets.

## METHODS AND MATERIALS

### Sample Description

The main sample comprised MRI and genotyping data from 22 cohorts from the ENIGMA-CNV consortium (12) (see Table S1 for cohort details). We considered 93 CNV regions (3) (Table S2) as having potential risk for NDDs (hereafter designated as NDD-CNVs). This includes reciprocal deletions/duplications of confirmed neurodevelopmental CNVs even if evidence for the pathogenicity of the reciprocal CNV is unclear (25). In the main dataset, comprising 9268 individuals, we identified 398 carriers of 36 NDD-CNVs (at 20 CNV loci). We considered individuals carrying none of the 93 CNVs as the noncarrier group. Demographic information is provided in Table 1. Neuroimaging data were collected from 40 acquisition sites up until September 30, 2019, with different ascertainment methods (family, clinical, population studies, and case-control studies for psychiatric disorders) (Table S1). Information on psychiatric or neurological medical conditions was based on available reports from different cohorts. We conducted an additional analysis including independent MRI data from the ENIGMA-22q consortium, comprising 274 individuals carrying the 22q11.2 (3 Mb) deletion, as well as 291 matched noncarrier controls. Demographic information for cohorts included in ENIGMA-22q is described in Table S6, and details of exclusion criteria, genotyping, and scanner parameters are described in Sun *et al*. (16) and Ching *et al*. (17).

### Genotyping, CNV Calling, and CNV Quality Control

Genotypes were obtained using commercially available platforms and conducted at each participating site (Table S1). All cohorts had CNVs called and identified in a unified manner as described previously in Sønderby *et al*. (12). Briefly, CNVs were called using PennCNV (26) and appropriate population frequency files and GC (content) model files (Table S3). Samples were filtered and CNVs identified based on standardized quality control metrics. CNVs overlapping the regions of interest were identified with the R package iPsychCNV, Select-SamplesFromRoi with parameters OverlapMin = 0.4 and OverlapMax = 5. Individuals with a minimum overlap of 0.4 to regions with known pathogenic CNVs were excluded regardless of copy number status.

### Image Acquisition and Processing

Structural T1-weighted MRI data were collected and processed locally at each site (12) using standardized neuroimaging protocols from the ENIGMA consortium (http://enigma.ini.usc.edu/protocols/imaging-protocols/), using FreeSurfer software (27). Brain measures consisted of volumes for left and right hemispheres of 7 subcortical regions and surface area and thickness for left and right hemispheres of 34 cortical regions, as well as total cortical surface area, mean cortical thickness, and ICV according to the Desikan-Killiany atlas (28). Scanner parameters and processing details are described in Table S4.

### CNV-Penetrance Scores

Penetrance scores represent the probability risk of developing either schizophrenia (PenSZ) or other developmental disorders (PenDDs) for individuals carrying a specific CNV. These scores were previously calculated in Kirov *et al*. (24) and were recently updated using control frequency from UK Biobank data in Kendall *et al*. (25). Penetrance scores are documented in Table 1. Briefly, the authors utilized data from large studies/samples of patients with schizophrenia and developmental disorders (including DD/ASD/CMs) to estimate the frequency of 70 CNVs in these disorder populations. Penetrance scores for either schizophrenia or developmental disorders were calculated on the basis of these frequencies by multiplying the frequency of a specific CNV in the disease population (either schizophrenia or developmental disorders) by the disease frequency in the general population (estimated at 1% for schizophrenia and 4% for DD/ASD/CMs) (24) and dividing by the frequency of the CNV in the general population (24).

### Statistical Analyses

Statistical analyses were performed in R version 4.1.2 (29). Prior to analyses, left and right hemispheric measures were averaged, and individual measures were excluded if they deviated more than ±4 SDs from the mean for each individual scanner site. We used ComBat harmonization to account for scanner effects while preserving differences between noncarriers (controls) and CNV carriers, as well as age and sex (30). Effects of age, age^2^, sex, and ICV were regressed out separately using linear regression on ComBat harmonized data. ICV was not regressed out when analyzing ICV. We used the entire sample for data harmonization, including data from noncarriers, to preserve biological differences across CNVs that were not explained by age, sex, or scanner differences. Covariance-corrected residuals were normalized and used in downstream analysis for each brain measure. Penetrance scores were log-transformed and normalized before analyses. Data from ENIGMA-22q were not included in the main analysis given the high number of 22q11.2 deletion carriers because such a highly penetrant CNV would likely influence the analysis. A separate analysis was performed including this dataset.

General linear models were used to identify associations between normalized log-transformed penetrance scores and normalized brain measures (23). To ensure that effects were associated with CNV penetrance and not simply due to the presence of NDD-CNVs, noncarriers were removed from the analyses. NDD-CNV carriers (as a group) were also compared with noncarriers to verify this assumption using a binary classification and correcting for age, age^2^, sex, and ICV.

We used the Benjamini–Hochberg false discovery rate (FDR) (*q* < .05) to account for multiple testing, taking 78 brain measures into account (7 subcortical volumes, 34 regional cortical surface area, 34 regional cortical thickness, and 3 global measures, see above). We also provide adjusted *p* values using Bonferroni correction in the main analysis, which is a more conservative approach. Regional cortical visualization was done with the R package “fsbrain” (version 0.5.3) (31).

We conducted sensitivity analyses repeating the main analysis after excluding 1) participants who were younger than 18 years, 2) individuals with known neurological or psychiatric diagnoses, 3) first-degree and second-degree relatives, 4) individual CNVs to assess whether individual CNVs were driving significant associations, and 5) CNVs with <3 individuals. We also repeated the analyses without regressing out the effects of ICV.

## RESULTS

### Sample Characteristics

The main dataset consisted of 398 carriers of 36 NDD-CNVs (20 deletions and 16 duplications at 20 CNV loci) and 8870 noncarriers (Table 1). In this sample, 601 individuals had a medical diagnosis (6.5%), 57 of whom were NDD-CNV carriers; among these, 481 individuals (447 noncarriers and 34 NDD-CNV carriers) had a neurological disorder, NDD, or psychiatric diagnosis (Table S5). The sample comprised 1920 individuals younger than 18 years (20.7%), 82 of whom were NDD-CNV carriers. There was a negative correlation between age and both PenSZ and PenDD (*t* = −3.03, *p* < .001 and *t* = −3.04, *p* < .001, respectively), indicating that carriers of highly penetrant CNVs were younger on average.

### CNV Penetrance Is Associated With Total Surface Area and Intracranial Volume

**Figure 1.**
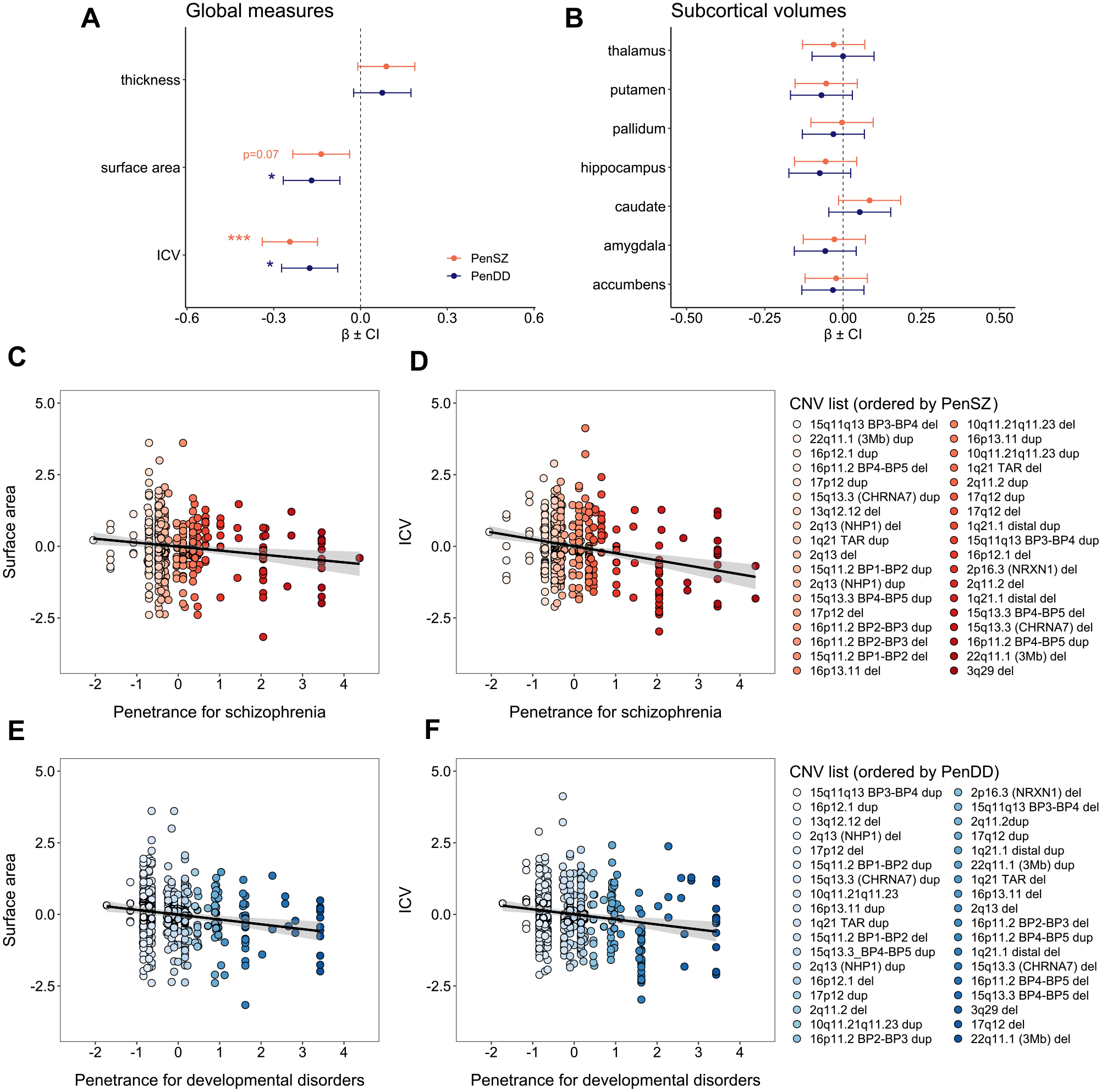
Copy number variant (CNV)-penetrance scores for schizophrenia (PenSZ) and developmental disorders (PenDD) (including developmental delay, autism spectrum disorder, and congenital malformations) are associated with total cortical surface area and intracranial volume (ICV). Standardized beta coefficients (βs) derived from the linear regression analysis for associations between penetrance scores (PenSZ and PenDD) and **(A)** global brain measures (mean cortical thickness, total cortical surface area, and ICV) and **(B)** subcortical volumes. *p < .05, ***p < .001 **(C**–**F)** Scatterplots showing linear associations between normalized logarithmic-transformed penetrance scores and normalized scanner harmonized and covariance-corrected residuals for both total surface area and ICV. Increased PenSZ for each CNV is represented with increasing red color intensity, and increased PenDD is represented with increasing blue color intensity.

Among 398 NDD-CNV carriers, increased PenDD was associated with lower cortical surface area (PenDD: β = −0.17, *t* = −3.39, *p*_FDR_ = .01). PenSZ had a marginal effect on cortical surface area (PenSZ: β = −0.14, *t* = −2.72, *p*_FDR_ = .07). Both PenDD and PenSZ were associated with lower ICV (PenSZ: β = −0.24, *t* = −5.01, *p*_FDR_ < .001; PenDD: β = −0.18, *t* = −3.56, *p*_FDR_ = .01). There were no significant associations between penetrance scores and mean cortical thickness or subcortical volumes (Figure 1 and Table S7). When we compared all NDD-CNV carriers (as a group) to noncarriers, there were no significant effects on global measures (Table S8A).

### Associations Between CNV Penetrance and Surface Area Are Strongest in the Occipital Lobes

**Figure 2.**
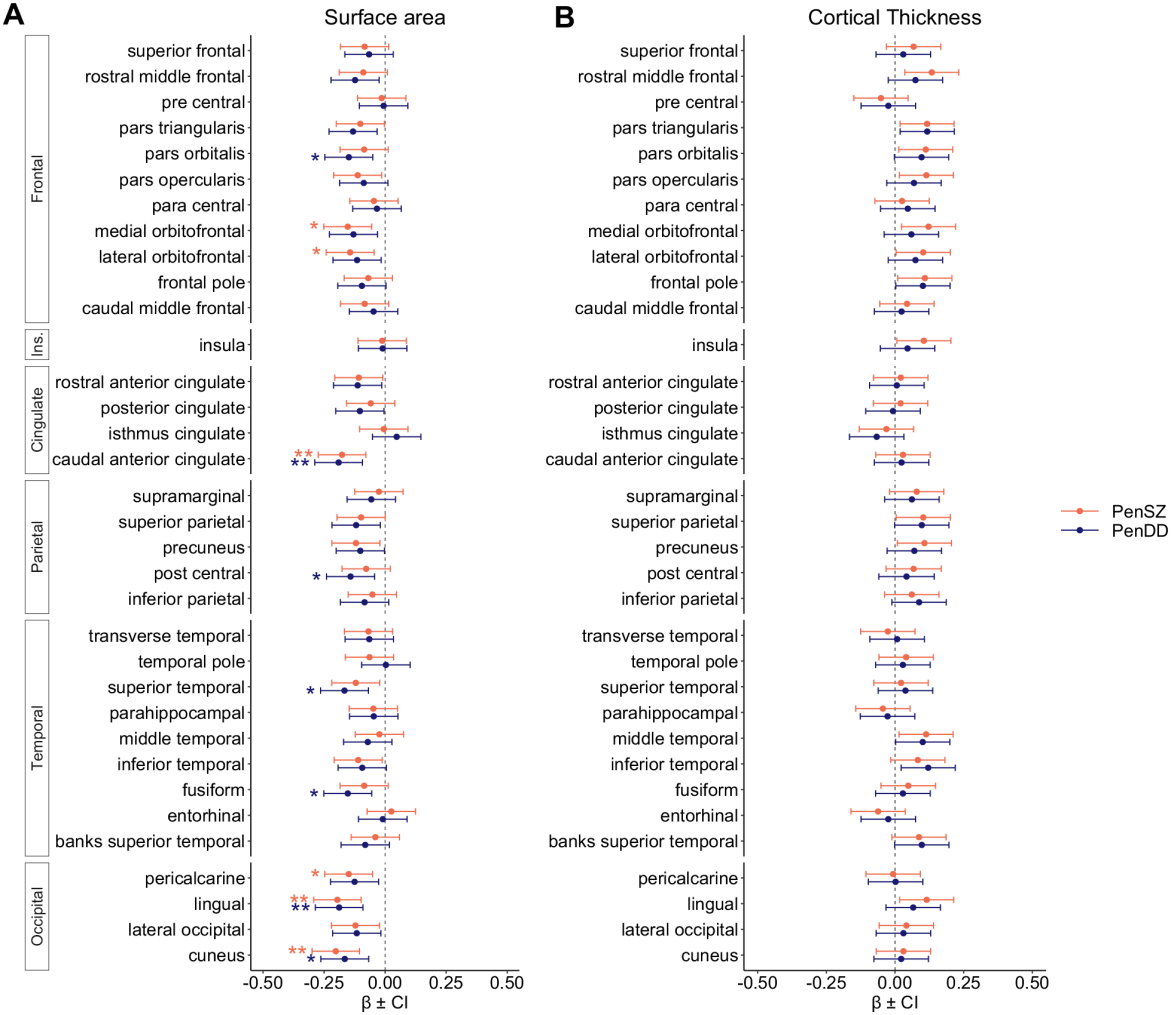
Associations between copy number variant-penetrance scores and surface area measures are strongest in the occipital lobes. Effect sizes (standardized βs) for linear associations between penetrance scores for schizophrenia (PenSZ) and developmental disorders (PenDD) (including developmental delay, autism spectrum disorder, and congenital malformations) and **(A)** regional cortical surface area and **(B)** regional cortical thickness measures. *p < .05, **p < .01.

**Figure 3.**
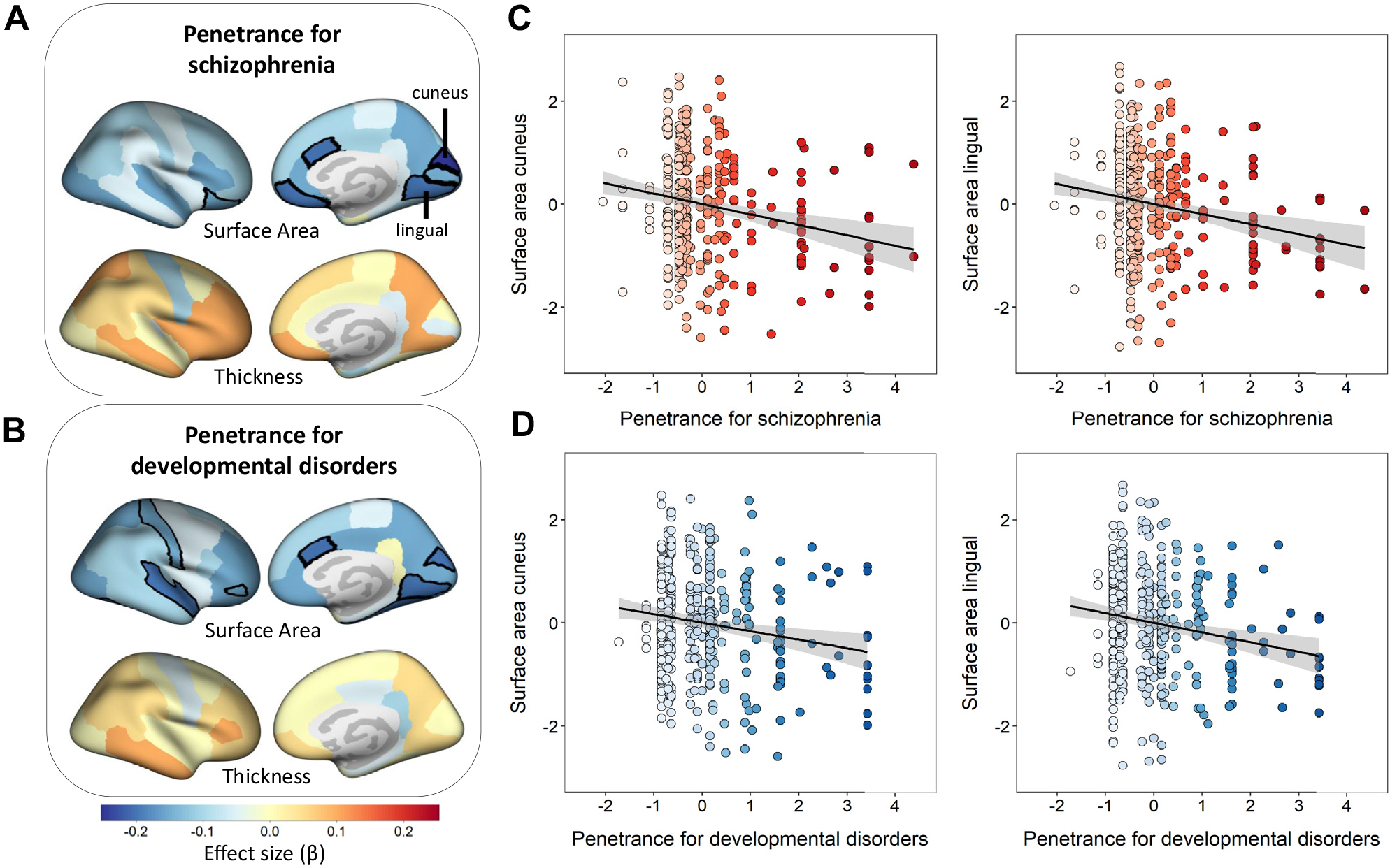
Associations between copy number variant (CNV) penetrance and surface area in the cuneus and in the lingual gyrus were the most robust findings. Brain plots showing effect sizes (standardized βs) for linear associations between CNV-penetrance scores for **(A)** schizophrenia (PenSZ) and **(B)** developmental disorders (PenDD) (including developmental delay, autism spectrum disorder, and congenital malformations) and regional cortical surface area and thickness. Significant areas (after false discovery rate correction) are delineated with black lines. Scatterplots showing linear associations between normalized logarithmic-transformed CNV-penetrance scores for **(C)** schizophrenia and **(D)** developmental disorders and normalized scanner harmonized covariance-corrected residuals for surface area in the cuneus and lingual gyrus. Increased PenSZ for each CNV is represented with increasing red color intensity, and increased PenDD is represented with increasing blue color intensity.

The largest effects for regional cortical surface area were found in the occipital lobes (Figures 2 and 3), where higher PenSZ was associated with lower surface area in the lingual gyrus, cuneus, and pericalcarine area (β = −0.2, *t* = −3.95, *p*_FDR_ =.002; β = −0.2, *t* = −4.09, *p*_FDR_ = .002; and β = −0.15, *t* = −2.99, *p*_FDR_ = .04, respectively). Higher PenDD was associated with lower surface area in the lingual gyrus and cuneus (β = −0.19, *t* = −3.78, *p*_FDR_ = .007 and β = −0.17, *t* = −3.3, *p*_FDR_ = .01, respectively). Additionally, higher PenSZ was associated with lower surface area in the frontal lobe (medial orbitofrontal and lateral orbitofrontal) and cingulate cortex (caudal anterior cingulate). Higher PenDD was associated with lower surface area in the frontal lobe (pars orbitalis), cingulate cortex (caudal anterior cingulate), parietal lobe (postcentral), and temporal lobe (superior temporal and fusiform gyrus). No significant associations were found between penetrance scores and regional cortical thickness (Figure 2 and Table S7).

When comparing NDD-CNV carriers to noncarrier controls, there were no significant effects on regions associated with CNV penetrance. Significant effects were found in surface area (smaller in NDD-CNV carriers) in the lateral occipital, precentral, and temporal pole and in cortical thickness (smaller in NDD-CNV carriers) in the parahippocampal and frontal pole (Table S8A).

### Inclusion of Data From 22q11.2 Deletion Carriers From the ENIGMA-22q Consortium

The inclusion of a large number of 22q11.2 deletion carriers substantially influenced the results in that several new associations became significant (Table S9). New associations were not only found in regional cortical surface area measures but also in subcortical volumes and cortical thickness measures. The association between PenSZ and surface area in the lateral orbitofrontal became nonsignificant following the inclusion of ENIGMA-22q data.

### Sensitivity Analyses

The associations between penetrance scores and surface area of the cuneus and lingual gyrus in the occipital lobes were the most robust findings (Tables S10–S15) in that the association between PenSZ and surface area in the cuneus survived all sensitivity tests (it was only reduced to trend level when we excluded carriers younger than 18 years; PenSZ association in the cuneus: β = −0.17, *t* = −3.05, *p*_FDR_ = .06). When we excluded carriers younger than 18 years, associations between brain features and both penetrance scores were still nominally significant while showing the same trend of effect (Table S10). Some of the associations became nonsignificant after excluding individuals with NDDs and neuropsychiatric conditions (Table S11). However, the associations between both penetrance scores and surface area in the cuneus and lingual gyrus remained significant, as well as the association with ICV. There was no effect on the direction of results from excluding any CNVs (results not shown), but the exclusion of 1q21.1 distal and 22q11.2 deletions had an impact on the size of the association effects (Table S13): the association between penetrance scores and ICV was largely influenced by the presence of 1q21.1 distal deletion carriers, whereas associations with PenSZ and PenDD were no longer significant after removing this CNV; however, the direction of effect was preserved (PenSZ: β = −0.11, *t* = −2.24, *p*_FDR_ = .2; PenDD: β = −0.07, *t* = −1.29, *p*_FDR_ = .5). The omission of 1q21.1 distal or 22q11.2 deletion carriers also affected associations between PenDD and surface area in the cuneus and lingual gyrus; results were no longer significant after omission (the association between PenSZ and surface area in the cuneus remained significant even after the omission of 1q21.1 distal or 22q11.2 carriers) (Figure S1).

Carriers of highly penetrant CNVs were younger than carriers of lower-penetrant CNVs even after removing carriers younger than 18 years (PenSZ ~ age: *t* = −3.17, *p* = .002; PenDD ~ age: *t* = −2.69, *p* = .008). To assess whether associations between penetrance scores and brain measures could be caused by age differences, we looked at age effects on brain measures in noncarriers. Generally, each brain measure decreased significantly with age (Table S16), meaning that older participants had lower cortical surface area and lower cortical thickness than younger participants on average.

Because cortical surface area and ICV are known to be correlated (32), we repeated the analyses without adjusting for ICV. This led to more brain features showing significant associations with both PenSZ and PenDD, in particular wide-spread associations in surface area (Table S15A). Given the influence of 1q21.1 distal deletion on ICV associations with CNV penetrance, we repeated the analysis without correcting for ICV and excluding individuals carrying the 1q21.1 distal deletion. Results were similar to the ones that were seen in the main analysis, with a few more regions (lateral occipital, postcentral, precuneus, superior parietal, and total surface area) showing associations between PenSZ and surface area (Table S15B).

## DISCUSSION

We assessed whether brain morphology was associated with risk for NDDs, as measured by penetrance scores for schizophrenia and other developmental disorders (including DD, ASD, and CMs), in individuals carrying NDD-CNVs. To our knowledge, this study analyzed the broadest cross-CNV neuroimaging sample to date, including 398 carriers of 36 NDD-CNVs. Higher PenSZ and higher PenDD were each associated with both smaller cortical surface area and smaller ICV, whereas no associations were found for cortical thickness measures. Associations between both penetrance scores and surface area were strongest in the occipital lobes, specifically in the cuneus and lingual gyrus. When we compared NDD-CNV carriers (as a group) to noncarriers, no significant effects were found in brain measures/regions showing associations with CNV penetrance, suggesting that these findings are related to CNV penetrance and not simply due to the presence of an NDD-CNV.

Our findings suggest that higher risk for both schizophrenia and developmental disorders is associated with smaller cortical surface area and smaller ICV in NDD-CNV carriers. The association with ICV was influenced by the 1q21.1 distal deletion, which is a CNV known to cause decreases in head circumference (33) and ICV (12). Our findings are consistent with previous ENIGMA-CNV and ENIGMA-22q studies that used the same sample; carriers of the 15q11.2 BP1-BP2, 1q21.1 distal, and 22q11.2 deletions showed lower total cortical surface area, and 1q21.1 distal deletions showed lower ICV than noncarrier controls (12,14,16). Our findings are also consistent with a UK Biobank study in which Caseras *et al*. (19) showed that carriers of 6 schizophrenia-associated CNVs (as a group) had smaller total cortical surface area and increased mean cortical thickness compared with noncarriers. When we compared the carriers of all 36 NDD-CNV to noncarriers, we did not find significant differences in global measures. However, our sample included predominantly lower-penetrant CNVs (Table 1), and some of these CNVs may lead to opposite effects in the brain. We repeated the analysis including only CNVs that were analyzed in the Caseras *et al*. study and found reduced surface area in a few regions and increased regional cortical thickness as well as decreased ICV in CNV carriers (Table S8B). Our approach of characterizing brain features based on penetrance scores (rather than treating all CNVs as a homogeneous group) allows us to distinguish brain features that are most related to pathogenicity (in our case reduced cortical surface area) from those that are not (in our case variations in cortical thickness, which were not significantly associated with CNV penetrance).

In a large-scale study from ENIGMA-Schizophrenia (34), patients with schizophrenia showed global decreases in cortical surface area, consistent with our findings, and wide-spread cortical thinning. Disease severity and antipsychotic medication treatment were associated with cortical thinning but not with surface area. In our study, although not statistically significant, there were trend-level increases in cortical thickness with higher CNV penetrance. Notably, 22q11.2 deletion carriers have smaller cortical surface area but wide-spread higher cortical thickness; however, 22q11.2 deletion carriers with psychotic illness have lower cortical thickness than those without psychosis, with no differences in cortical surface area (16). ENIGMA-attention-deficit/hyperactivity disorder (ADHD) found lower surface area and cortical thinning in children with ADHD, with no differences in adolescent or adult groups (35). Notably, lower surface area was also found in unaffected siblings, suggesting that changes in surface area occur independently of disease onset. Reduced cortical surface area in patients with schizophrenia and ADHD and NDD-CNV carriers may indicate a premorbid risk for neurodevelopmental conditions, whereas reduced cortical thickness in patients with schizophrenia (contrasting with increased thickness in NDD-CNV carriers) may be influenced by disease onset, illness progression, medication, and age.

Surface area and thickness are distinct features of cortical structure (32,36). A large-scale genome-wide association study of MRI data (32) suggests that surface area is influenced by genetic variants involved in neural progenitor cell activity during fetal development, whereas thickness is influenced by adult-specific processes (e.g., pruning, branching, or myelination). Notably, the authors found genetic correlations and evidence for causation of surface area with both general cognitive functioning and educational attainment, as well as correlations with other traits and disorders. The thickness of some regions showed genetic correlations with general cognitive function and educational attainment but no evidence of causal relationship, adding to the hypothesis that cortical thickness changes reflect environmental influences or effects of illness progression/treatment.

Associations between cortical surface area and CNV penetrance could indicate shared disease mechanisms affecting corticogenesis, which may be points of convergence across NDDs and CNVs (37,38). Evidence suggests that CNVs affect progenitor cell proliferation: an LgDel mouse model of the 22q11.2 deletion exhibited deficits in intermediate progenitor cell proliferation (39), and cortical surface area alterations in human 22q11.2 deletion carriers were associated with expression of genes involved in cell proliferation and apoptosis (40). Moreover, 1q21.1 distal deletions altered neural progenitor cell proliferation in induced pluripotent stem cell–derived cells (41), and 16p11.2 BP4-BP5 CNVs altered the proportion of neurons and progenitor populations in cerebral organoids (42). Disruptions in cell proliferation may profoundly affect brain size, resulting in macro- and microcephaly, which are known phenotypes associated with some CNVs (e.g., 1q21.1 distal and both 16p11.2 BP2-BP3 and BP4-BP5) (33,43). More research is needed to understand how CNVs affect cortical development and developmental trajectories.

We found the strongest and most robust associations between CNV penetrance and surface area in the medial occipital lobes (cuneus and lingual gyrus), which include major early-forming sulci of the brain. The medial occipital lobes are centers for long-range association fibers (44), which supports involvement in roles beyond basic visual processing, such as language and memory (45). Both the lingual gyrus and cuneus are involved in processing of emotional facial expressions (46,47), which is disrupted in schizophrenia (48) and ASD (49). Volumetric abnormalities in the occipital lobes predict severity of ultra-high-risk prodromal symptoms of psychosis in 22q11.2 deletion carriers (50). In a linear mixed effects model, we found a significant interaction between brain region and CNV penetrance on surface area (PenSZ: region, *p* < .001; PenDD: region, *p* < .001). However, larger effect sizes in specific regions could be affected by the accuracy of ComBat harmonization, which may vary by region (51).

This study has some limitations. Carriers of highly penetrant NDD-CNVs were younger than less-penetrant NDD-CNV carriers. Age effects were accounted for before statistical analysis, using data from noncarriers to preserve CNV effects. Furthermore, cortical surface area decreases with age (Table S16), suggesting that age effects are unlikely to explain the association between increased CNV penetrance and reduced cortical surface area. Nevertheless, NDD-CNV carriers may display altered trajectories of cortical development (52), and evidence from ENIGMA-ADHD and ENIGMA-Schizophrenia suggest that there are age-dependent effects in these conditions (34,35). Our sample includes a wide age range in most CNVs (Figure S2), but it does not include sufficient numbers of carriers in each age bracket across CNVs to reliably investigate effects of age. Future studies with age-balanced samples are needed to examine possible age and penetrance interaction effects. ENIGMA-CNV is a multisite consortium with carriers and noncarriers scanned at each site. Nevertheless, the inclusion of both clinically and nonclinically ascertained cohorts might have introduced some bias. However, we found similar results after excluding participants with known psychiatric or neurological diagnoses (Table S11). Factors related to medication could not be investigated because medication information was not universally available. Although our study comprised a large number of CNVs, CNVs were not equally represented, and as was expected given the sample ascertainment, there were more carriers of lower-penetrance CNVs than of rarer, higher-penetrance CNVs (Table 1). Therefore, although our study had adequate power to detect an effect of CNV penetrance, it was not powered to detect effects of individual higher-penetrant CNVs. The inclusion of a large number of 22q11.2 deletion carriers (a highly penetrant CNV) from the ENIGMA-22q dataset (*n* = 274) led to additional significant findings (Table S9). However, these findings may be related to specific effects of the 22q11.2 locus (16,17). Future studies with a higher number of carriers across higher-penetrant CNVs are needed to reliably study the effects of CNV penetrance on the brain. Some effects may be dosage-dependent given reports that deletions and duplications can lead to opposite effects for certain brain traits in 22q11.2 (16,17), 16p11.2 proximal (43,53,54) and distal (13), and 15q11.2 BP1-BP2 (14,55) CNVs. Notably, the effect sizes for the associations that were found in this study are considered small according to Cohen’s criteria (56), even for the strongest associations that we found in the cuneus and lingual (β ≈ −0.2). This is in contrast with previous studies on CNV versus non-CNV carrier comparisons, where effect sizes were moderate to strong (57). Future studies that include more carriers per CNV are needed to understand relationships between brain alterations, issues related to gene dosage, the potential role of other genetic variants, and risk for NDDs.

### Conclusions

Increased risk for schizophrenia and other developmental disorders (including DD, ASD, and CMs) in CNV carriers, as measured through penetrance scores, was associated with variations in brain morphology, specifically with lower ICV and lower cortical surface area. Penetrance for schizophrenia and developmental disorders was associated with lower cortical surface area in parts of the occipital and frontal lobes, as well as in the anterior cingulate cortex. Penetrance for developmental disorders was also associated with lower cortical surface area in parts of the parietal and temporal lobes. Our findings suggest shared mechanisms across NDD-CNVs that affect cortical development.

## Supplementary Material

Supplement

Supplementary material cited in this article is available online at https://doi.org/10.1016/j.bpsc.2025.05.010.

## Figures and Tables

**Table 1.** Demographic Data for NDD-CNV Carriers and Noncarrier Control Participants From the ENIGMA-CNV and ENIGMA-22q Cohorts

	Deletions					Duplications				
NDD-CNVs	*n*	PenSZ	PenDD	Age, Years, Mean (SD)	Sex	*n*	PenSZ	PenDD	Age, Years, Mean (SD)	Sex
ENIGMA-CNV (Main Sample)										
Carriers										
1q21 TAR	2	1.89%	14.85%	58.8 (2.51)	2 male	9	1.00%	5.86%	45 (24.4)	3 female, 6 male
1q21.1 BP3-BP4 (Distal)	19	4.95%	22.91%	24.5 (14.4)	9 female, 10 male	10	2.24%	13.58%	41.7 (18.2)	4 female, 6 male
2p16.3 (*NRXN1*)	2	3.54%	9.77%	33.3 (14.6)	1 female, 1 male	–	–	–	–	–
2q11.2	2	3.60%	9.18%	29.5 (12.1)	2 male	1	1.91%	11.67%	47	1 male
2q13 (*NHP1*)	72	0.99%	3.25%	42.5 (19.4)	36 female, 36 male	48	1.12%	7.59%	41.4 (20.3)	25 female, 23 male
2q13	1	1.00%	15.87%	26.4	1 female	–	–	–	–	–
3q29	2	15.35%	48.65%	34.4 (20.6)	2 male	–	–	–	–	–
10q11.21q11.23	5	1.74%	5.32%	42.8 (15.5)	3 female, 2 male	1	1.85%	9.20%	14.1	1 female
13q12.12	3	0.97%	3.21%	34.7 (17.1)	3 female	–	–	–	–	–
15q11.2 BP1-BP2	26	1.56%	6.15%	39.2 (20.9)	15 female, 11 male	42	1.04%	3.67%	44.7 (22)	22 female, 20 male
15q11q13 BP3-BP4	1	0.00%	11.66%	68	1 female	1	2.33%	1.28%	37.2	1 female
15q13.3 BP4-BP5	2	5.09%	45.96%	30 (4.53)	2 male	7	1.15%	6.64%	33.4 (17)	3 female, 4 male
15q13.3 (*CHRNA7*)	1	6.67%	30.80%	14.7	1 female	53	0.79%	3.88%	38.2 (23.7)	27 female, 26 male
16p13.11	2	1.61%	15.83%	14.3 (0.69)	1 female, 1 male	21	1.85%	5.46%	41.1 (20.4)	14 female, 7 male
16p12.1	7	2.80%	7.96%	23.5 (8.45)	2 female, 5 male	6	0.52%	2.40%	40.9 (17.7)	5 female, 1 male
16p11.2 BP2-BP3 (Distal)	3	1.41%	21.98%	22.5 (9.45)	1 female, 2 male	8	1.40%	9.61%	36 (20.5)	4 female, 4 male
16p11.2 BP4-BP5 (Proximal)	3	0.77%	36.82%	46.4 (33)	2 female, 1 male	2	7.00%	22.10%	51.1 (2.75)	2 female
17p12	3	1.20%	3.54%	42 (22.3)	3 male	5	0.78%	8.15%	33.8 (22.4)	3 female, 2 male
17q12	2	2.05%	54.78%	31.7 (0.99)	2 female	9	1.99%	13.27%	45.9 (23.5)	3 female, 6 male
22q11.1 (3 Mb)	11	9.98%	83.98%	22.6 (14.1)	5 female, 6 male	6	0.20%	14.13%	33.7 (17.1)	4 female, 2 male
	*n*			Age, Years, Mean (SD)	Sex					
Noncarriers (Controls)	8870			40.6 (21.4)	4783 female, 4087 male					
ENIGMA-22q										
	*n*	PenSZ	PenDD	Age, Years, Mean (SD)	Sex					
22q11 (3 Mb) Deletion Carriers	274	9.98%	83.98%	18.52 (9.59)	144 female, 130 male					
Noncarriers (Controls)	291			18.34 (9.47)	132 female, 159 male					

PenSZ and PenDD for each CNV were previously calculated using large cohorts, as described in Kirov *et al*. (24), and recalculated in Kendall *et al*. (25) using control frequency from the UK Biobank data. Penetrance scores were calculated by multiplying the probability of carrying a specific CNV, given disease status, by the frequency of the disease in the population (which was estimated at 1% for schizophrenia and 4% for developmental disorders [including developmental delay, autism spectrum disorder, and congenital malformations]) (24).

CNV, copy number variant; ENIGMA, Enhancing Neuro Imaging Genetics through Meta Analysis; NDD, neurodevelopmental disorder; PenDD, penetrance scores for developmental disorders; PenSZ, penetrance scores for schizophrenia.
