# Supplementary material for: Penetrance of Neurodevelopmental Copy Number Variants Is Associated With Variations in Cortical Morphology
Source: Biol Psychiatry Cogn Neurosci Neuroimaging. Author manuscript; Available in PMC 2026 Apr 24. (PMC13107928; doi:10.1016/j.bpsc.2025.05.010)
Supplement: Supplement [file NIHMS2163160-supplement-Supplement.pdf]

SUPPLEMENTARY INFORMATION

Penetrance of Neurodevelopmental Copy Number Variants Is Associated With Variations in Cortical Morphology

Silva *et al.*

Supplementary Materials and Methods

Table S1 – Details of cohorts from ENIGMA-CNV that were included in this study. 3
Table S2 – CNVs included in this study. 7
Table S3 – Chips and corresponding PFB-files used for PennCNV CNV calling. 9
Table S4 – Technical details concerning scanners and acquisition parameters utilized at the participating ENIGMA-CNV scanner sites. 9
Table S5 – Number carriers that have an established diagnosis and a specific neurological or psychiatric diagnosis for each CNV in the ENIGMA-CNV dataset. 12
Table S6 – Details of cohorts from ENIGMA-22q (274 carriers of the 22q11.2 (3Mb) deletion and 291 non-carriers). 13

Supplementary Results

Table S7 – Main analyses results.. 18
Table S8 – Comparisons between CNV carriers and non-carrier controls. 20
Table S9 – Linear regression results including additional data from the ENIGMA-22q dataset. 23
Table S10 – Linear regression results after excluding CNV carriers younger than 18 years old. 25
Table S11 – Linear regression results after excluding individuals with neurodevelopment and neuropsychiatric conditions. 28
Table S12 – Linear regression results after excluding first-degree and second-degree relatives. 31
Table S13 – Linear regression results after excluding A)1q21.1 distal deletion carriers and B) 22q11.2 deletion carriers. 33
Table S14 – Linear regression results after excluding CNVs with n<3. 38
Table S15 – Linear regression results when not correcting for ICV. A) Main sample and B) when excluding 1q21.1 distal deletion. 41
Table S16 – Effects of age on brain measures. 46

Figure S1 – Effect size change due to each CNV omission from the main analysis in the association between penetrance scores for schizophrenia (PenSZ) and developmental disorders (PenDD) and cortical surface area of the cuneus and lingual gyrus.. 49
Figure S2 – Scatterplots showing linear associations between logarithmic-transformed CNV penetrance scores and age 50

Supplementary Materials and Methods

Table S1 – Details of cohorts from ENIGMA-CNV that were included in this study. Study design, participant demographics, and references to articles containing descriptions of individual inclusion and exclusion parameters for all datasets in ENIGMA-CNV collected up until Sep 30, 2019.

Cohort	Study design	Total n	n, females	Mean age	sd age	min age	max age	No of chips	Chip names	No of scanner sites	References
16p11.2Consortium	Neurodevelopmental cohort	78	37	30.8	14.8	6.4	65.4	5	Agilent oligo 400k;CGH array;FISH;MLPA;noinfo	2	1, 2
Cardiff/ECHO-DEFINE	Neurodevelopmental cohort	56	27	31.6	15.4	12.1	70.8	2	PsychChip;noinfo	1	3, 4
COBRE	Case-control (Schizophrenia and healthy controls)	158	38	38.1	13.1	18	65	2	HumanOmni1-Quad;HumanOmni5-4	1	5
Dublin	Case-control (SCZ, BD, other psychoses, and healthy controls)	108	53	37.3	12.2	18	64	1	Affymetrix6.0	1	6
GOBS	Family study	1149	664	39.9	15.5	18	84	1	HumanOmni2.5	1	7
Hubin	Case-control (SCZ and healthy controls)	200	59	41.6	8.36	19.4	56.3	1	PsychChip	1	8, 9
HUNT	Population-based	872	459	58.9	4.19	50.5	66.8	1	HumanOmni2.5	1	10, 11
IMAGEN	Population-based	1720	875	14.5	0.44	12.9	17.2	2	Illumina Human660W-Quad;Illumina HumanQuad 610	9	12
MAS	Population-based	505	292	78.3	4.72	70.5	90.1	1	Affymetrix6.0	2	13
MCIC	Case-control (SCZ and healthy controls)	216	72	33.2	10.9	18	59	1	HumanOmni1-Quad	4	14
METH-CT	Case-control (schizophrenia including individuals with methamphetamine dependence (MA) or methamphetamine-associated psychosis (MAP) and healthy controls)	99	18	26.4	6.45	18	53	1	PsychChip	1	16
OATS1	Population-based Twin Study	358	236	70.5	5.11	65	89	1	Illumina OmniExpress	4	17
OATS2	Population-based Twin Study	15	8	67.9	3.03	65	74	1	Illumina OmniExpress	3 (same as OATS1)	17
QTIM	Population-based Twin Study	965	622	22.3	3.32	15.4	30.1	2	Illumina Human Core Exome;Illumina HumanQuad 610	1	18
Stroke (TOP_T3)	Stroke project (only healthy controls)	51	32	45.6	22.1	18	78	1	Illumina OmniExpress	1	19, 20
GAP	Case-control (Schizophrenia and healthy controls)	96	37	27.7	7.65	18	54	1	Illumina Human Core Exome	1	

Haavik	ADHD	53	28	30.2	6.97	20	47	2	Illumina OmniExpress;PsychChip	1	21
NCNG	Population-based	363	244	52.6	17.2	19.4	82.3	1	Illumina HumanQuad 610	2	22
NTR	population-based Twin Study	245	143	26.9	10.9	9.05	56	1	Affymetrix6.0	5	23, 24, 25, 26, 27, 28
PAFIP	Case-control (SCZ and healthy controls)	134	58	29.9	7.93	17.7	54.2	1	Illumina OmniExpress Exome	1	29, 30
SHIP-2	Population-based	950	493	56.1	12.6	31	90	1	Affymetrix6.0	1	31
SHIP-Trend	Population-based	885	495	50.4	13.5	22	81	1	HumanOmni2.5	1 (same as SHIP-2)	31

References to Table S1:

1 Maillard, A.M., et al. The 16p11.2 locus modulates brain structures common to autism, schizophrenia and obesity. Mol Psychiatry 20, 140-147 (2015).

2 Martin-Brevet, S.R.-H., B. ; Nielsen, JA;. Quantifying the effects of 16p11.2 copy number variants on brain structure: A multi-site ‘genetic-first’ study. (in submission).

3 Chawner, S. J., et al. (In press (Lancet Psychiatry). "Genotype-phenotype relationships in children with Copy Number Variants associated with high neuropsychiatric risk: Findings from the case-control IMAGINE-ID cohort in the United Kingdom ".

4 Chawner, S., et al. (2018). "The emergence of psychotic experiences in the early adolescence of 22q11.2 Deletion Syndrome." J Psychiatr Res 109: 10-17.

5 Aine, C.J., et al. Multimodal Neuroimaging in Schizophrenia: Description and Dissemination. Neuroinformatics 15, 343-364 (2017).

6 Morris, DW et al. An inherited duplication at the gene p21 Protein-Activated Kinase 7 (PAK7) is a risk factor for psychosis. Hum Mol Genet. 2014 Jun 15;23(12):3316-26. doi: 10.1093/hmg/ddu025

7 McKay, D.R., et al. Influence of age, sex and genetic factors on the human brain. Brain Imaging Behav 8, 143-152 (2014).

8 Agartz, I. et al., BDNF gene variants and brain morphology in schizophrenia. American Journal of Medical Genetics Part B 141B, 513-523 (2006).

9 Jönsson, E. G. et al., Brain-derived neurotrophic factor gene (BDNF) variants and schizophrenia: an association study. Progress in Neuropsychopharmacology and Biological Psychiatry 30, 924-933 (2006).

10 Honningsvag, L.M., Linde, M., Haberg, A., Stovner, L.J. & Hagen, K. Does health differ between participants and non-participants in the MRI-HUNT study, a population based neuroimaging study? The Nord-Trondelag health studies 1984-2009. BMC medical imaging 12, 23 (2012).

11 Haberg, A.K., et al. Incidental Intracranial Findings and Their Clinical Impact; The HUNT MRI Study in a General Population of 1006 Participants between 50-66 Years. PloS one 11, e0151080 (2016).

12 Schumann, G., et al. The IMAGEN study: reinforcement-related behaviour in normal brain function and psychopathology. Mol Psychiatry 15, 1128-1139 (2010).

13 Sachdev, P.S., et al. The Sydney Memory and Ageing Study (MAS): methodology and baseline medical and neuropsychiatric characteristics of an elderly epidemiological non-demented cohort of Australians aged 70-90 years. International psychogeriatrics 22, 1248-1264 (2010).

14 Gollub, R.L., et al. The MCIC collection: a shared repository of multi-modal, multi-site brain image data from a clinical investigation of schizophrenia. Neuroinformatics 11, 367-388 (2013).

16 Uhlmann, A., et al. Fronto-temporal alterations and affect regulation in methamphetamine dependence with and without a history of psychosis. Psychiatry Res 248, 30-38 (2016).

17 Sachdev, P.S., et al. A comprehensive neuropsychiatric study of elderly twins: the Older Australian Twins Study. Twin Res Hum Genet 12, 573-582 (2009).

18 Renteria, M.E., et al. Genetic architecture of subcortical brain regions: common and region-specific genetic contributions. Genes, brain, and behavior 13, 821-830 (2014).

19 Richard, Geneviève, et al. "Assessing distinct patterns of cognitive aging using tissue-specific brain age prediction based on diffusion tensor imaging and brain morphometry." PeerJ 6 (2018): e5908

20 Beck, Dani, et al. "White matter microstructure across the adult lifespan: A mixed longitudinal and cross-sectional study using advanced diffusion models and brain-age prediction." NeuroImage 224 (2021): 117441

21 Dramsdahl M et al. Adults with attention-deficit/hyperactivity disorder - a diffusion-tensor imaging study of the corpus callosum. Psychiatry Res. 2012 Feb 28;201(2):168-73.

22 Espeseth, T. et al. Imaging and cognitive genetics: The Norwegian Cognitive NeuroGenetics sample. Twin Research and Human Genetics, 15, 442-452 (2012).

23 van 't Ent, D., et al. A structural MRI study in monozygotic twins concordant or discordant for attention/hyperactivity problems: evidence for genetic and environmental heterogeneity in the developing brain. NeuroImage 35, 1004-1020 (2007).

24 den Braber, A. et al. Brain activation during cognitive planning in twins discordant or concordant for obsessive-compulsive symptoms. Brain 133, 3123–3140 (2010).

25 de Geus, E. J. et al. Intrapair differences in hippocampal volume in monozygotic twins discordant for the risk for anxiety and depression. Biol.Psychiatry 61, 1062–1071 (2007).

26 Baare, W. F. et al. Quantitative genetic modeling of variation in human brain morphology. Cereb.Cortex 11, 816–824 (2001).

27 den Braber, A. et al.Heritability of subcortical brain measures: a perspective for future genome-wide association studies. Neuroimage 83, 98-102 (2013).

28 Abdellaoui, A. et al. CNV Concordance in 1,097 MZ Twin Pairs. Twin Research and Human Genetics 18, 1-12 (2015).

29 Pelayo-Teran, J.M.,et al. Epidemiological factors associated with treated incidence of first-episode non-affective psychosis in Cantabria: insights from the clinical programme on early phases of psychosis. Early Interv. Psychiatry 2 (3), 178–187. (2008)

30 Tordesillas-Gutierrez D,et al.Grey matter volume differences in non-affective psychosis and the effects of age of onset on grey matter volumes: A voxelwise study.Schizophr Res. 2015 May;164(1-3):74-82

31 Volzke, H., et al. Cohort profile: the study of health in Pomerania. International journal of epidemiology 40, 294-307 (2011).

Table S2 – CNVs included in this study. Individuals with a minimum overlap of 0.4 to these CNVs were excluded from the analysis. Coordinates are Human Genome Build NCBI36/hg18 and GRCh37/hg19.

CNVs present in the study sample	Locus	Chr	Start	Stop	Length	Start	Stop	Length	source
1q21 TAR	1q21.1	1	144106312	144519174	412862	145394955	145807817	412862	Kendall et al 2017
1q21.1 BP3-BP4 (distal)	1q21.1	1	144994611	145861068	866457	146527987	147394444	866457	Kendall et al 2017
2p16.3 (NRXN1)	2p16.3	2	49999147	51113178	1114031	50145643	51259674	1114031	Kendall et al 2017
2q11.2	2q11.2	2	96106136	97041243	935107	96742409	97677516	935107	Kendall et al 2017
2q13 (NPHP1)	2q13	2	110220005	110341237	121232	110862716	110983948	121232	Kendall et al 2017
2q13	2q13	2	111110510	111729120	618610	111394040	112012649	618609	Kendall et al 2017
3q29	3q29	3	197204564	198839223	1634659	195720167	197354826	1634659	Kendall et al 2017
10q11.21q11.23	10q11.21q11.23	10	49060205	50728802	1668597	49390199	51058796	1668597	Kendall et al 2017
13q12.12	13q12.12	13	22453358	23782622	1329264	23555358	24884622	1329264	Kendall et al 2017
15q11.2 BP1-BP2	15q11.2	15	20356677	20645971	289294	22805313	23094530	289217	Kendall et al 2017
15q11q13 BP3-BP4	15q13.1q13.2	15	26996736	28163259	1166523	29161368	30375967	1214599	Kendall et al 2017
15q13.3 BP4-BP5	15q13.3	15	28867937	30250068	1382131	31080645	32462776	1382131	Kendall et al 2017
15q13.3 (CHRNA7)	15q13.3	15	29804362	30240360	435998	32017070	32453068	435998	Kendall et al 2017
16p13.11	16p13.11	16	15419156	16201190	782034	15511655	16293689	782034	Kendall et al 2017
16p12.1	16p12.1	16	21857636	22339390	481754	21950135	22431889	481754	Kendall et al 2017
16p11.2 BP2-BP3 (distal)	16p11.2	16	28730697	28954284	223587	28823196	29046783	223587	Kendall et al 2017
16p11.2 BP4-BP5 (proximal)	16p11.2	16	29558341	30108274	549933	29650840	30200773	549933	Kendall et al 2017
17p12	17p12	17	14082112	15367686	1285574	14141387	15426961	1285574	Kendall et al 2017
17q12	17q12	17	31890017	33291545	1401528	34815904	36217432	1401528	Kendall et al 2017
22q11 (3Mb)	22q11.2	22	17417332	19796726	2379394	19037332	21466726	2429394	Kendall et al 2017
**Other neurodevelopmental CNVs not present in the study**									
1p36 (GABRD)	1p36	1	0	2489861	2489861	0	2500000	2500000	Kendall et al 2017
2q37 (HDAC4)	2q37	2	239381424	242864118	3482694	239716679	243199373	3482694	Kendall et al 2017
4p16.3 Wolf-Hirschhorn	4p16.3	4	1522161	2061101	538940	1552030	2091303	539273	Kendall et al 2017
5q35 (Sotos)	5q35	5	175653530	176985200	1331670	175720924	177052594	1331670	Kendall et al 2017
6q16 (SIM1)	6q16	6	100943471	101018532	75061	100836750	100911811	75061	Kendall et al 2017
7q11.23 (Williams-Beuren syndrome)	7q11.23	7	72382851	73780828	1397977	72744915	74142892	1397977	Kendall et al 2017
7q11.23	7q11.23	7	74976230	75902348	926118	75138294	76064412	926118	Kendall et al 2017
8p23.1	8p23.1	8	8136400	11909967	3773567	8098990	11872558	3773568	Kendall et al 2017
9q34 (EHMT1)	9q34	9	139633265	139850399	217134	140513444	140730578	217134	Kendall et al 2017

10q23 (NRG3,GRID1)	10q22q23	10	82035452	88921631	6886179	82045472	88931651	6886179	Kendall et al 2017
11p11.2 (EXT2)	11p11.2	11	43896576	45976576	2080000	43940000	46020000	2080000	Kendall et al 2017
13q12.11 (ZMYM5)	13q12	13	19875806	19998012	122206	20977806	21100012	122206	Kendall et al 2017
15q11.2-13.1_BP1-2 (PWS/AS)	15q11.2q12	15	20356677	26063934	5707257	22805313	28390339	5585026	Kendall et al 2017
15q11q13 BP3-BP5	15q13.1q13.3	15	26996736	30250068	3253332	29161368	32462776	3301408	Kendall et al 2017
15q24	15q24	15	70687225	75938308	5251083	72900171	78151253	5251082	Kendall et al 2017
15q25	15q25.2	15	81016790	83523043	2506253	83219735	85722039	2502304	Kendall et al 2017
16p13.3 (Rubinstein Taybi) (CREBBP)	16p13.3	16	3715057	3870122	155065	3775056	3930121	155065	Kendall et al 2017
16p12.2-p11.2 (7.1-8.7Mb)	16p11.2p12.1	16	21503916	28255309	6751393	21596415	28347808	6751393	Kendall et al 2017
16p11.2_-_distal_large	16p11.2	16	28360697	28954284	593587	28453196	29046783	593587	Sønderby et al 2018
16p11.2_entireregion	16p11.2	16	28360697	30108274	1747577	28453196	30200773	1747577	Sønderby et al 2018
17p13.3 (YWHAE)	17p13.3	17	1194584	1250306	55722	1247834	1303556	55722	Kendall et al 2017
17p13.3 (PAFAH1B1)	17p13.3	17	2443673	2535659	91986	2496923	2588909	91986	Kendall et al 2017
17q11.2 (NF1)	17q11.2	17	26131617	27289188	1157571	29107491	30265075	1157584	Kendall et al 2017
17q21.31	17q21.31	17	41061139	41520509	459370	43705356	44164691	459335	Kendall et al 2017
17q23.1q23.2	17q23.1q23.2	17	55657171	57643923	1986752	58302389	60289141	1986752	Kendall et al 2017
22q11_distal	22q11.2	22	20250127	21983646	1733519	21920127	23653646	1733519	Kendall et al 2017
SHANK3	22q13	22	49459936	49518506	58570	51113070	51171640	58570	Kendall et al 2017

Table S3 – Chips and corresponding PFB-files used for PennCNV CNV calling.

Chip	PFB-file
Illumina Human660W-Quad	hhall.hg18.pfb
Illumina HumanQuad 610	hhall.hg18.pfb
Affymetrix6.0	affygw6.hg18.pfb
Affymetrix6.0	self-generated (Japanese cohort)
Axiom	self-generated (ukb)
Illumina Human Core Exome	humancoreexome-12v1-1_a_hg18.pfb_adj
HumanHap550	hhall.hg18.pfb
HumanOmni1-Quad	OmniExpress_hg18.pfb
HumanOmni2.5	OmniExpress_hg18.pfb, hhall.hg18.pfb
HumanOmni5-4	OmniExpress_hg18.pfb
Illumina OmniExpress	OmniExpress_hg18.pfb
Illumina OmniExpress Exome	OmniExpressHumanCoreExome_hg18.pfb
PsychChip	humancoreexome-12v1-1_a_hg18.pfb_adj

Table S4 – Technical details concerning scanners and acquisition parameters utilized at the participating ENIGMA-CNV scanner sites.

Dataset	Scanner Site	Sequence	FieldStrength	Acquisition Direction	NumberOfSlices	Slice Gap	Voxel Size_m3	TI	TE	TR	Flip Angle	Segmentation
16p11.2Consortium	16p11_consortium_b	3D T1-weighted magnetization prepared rapid acquisition gradient echo (MPRAGE)	3T Siemens Magnetom Prisma Syngo	Sagittal	176	0.5	1x1x1	920 ms	2.39 ms	2000 ms	9	Freesurfer (5.1.0) freesurfer-x86_64-redhat-linux-gnu-stable5-20110522
	16p11_consortium_a	3D T1-weight multi-Echo Magnetization Prepared RApid Gradient Echo sequence (ME-MPRAGE)	3T Magnetom TIM Trio	Sagittal	176	0.5	1x1x1	1200 ms	TE1 = 1.64 ms, TE2 = 3.5 ms, TE3 = 5.36 ms, TE4 = 7.22 ms	2530ms	7	Freesurfer (5.1.0) freesurfer-x86_64-redhat-linux-gnu-stable5-20110522
Cardiff/ECHO-DEFINE	ECHO_DEFINE	3D T1-weighted fast spoiled gradient recall (3D FSPGR)	3T GE SIGNA HDx		256	1mm	1x1x1	450 ms	2.984ms	7.816ms	20	FreeSurfer(5.3.0)
COBRE	COBRE	3D T1-weighted magnetization prepared rapid acquisition gradient echo (MPRAGE)	3T Siemens Trio	Sagittal	192	0	1x1x1		5 echoes, 1.64/3.5/5.36/7.22/9.08 ms	2520ms	7	FreeSurfer (5.3.0)

Dublin	Dublin	3D T1-weighted Fast Field Echo	Philips Achieva 3T system	Sagittal	180		0.9x0.9 x0.9	516 ms	3ms	8.4ms	8	FreeSurfer (5.4.0)
GOBS	GOBS	T1-weighted MPRAGE (also, "3D turbo-flash sequences with an adiabatic inversion contrast pulse")	3T TIM Treo	Axial	160	0mm	1.7x1.7 x3	785	3.04	2100ms	13	FreeSurfer (5.3.0)
Hubin	Hubin	3D T1-weighted fast spoiled gradient recall (3D FSPGR)	1.5T GE signa Echo-speed	Coronal	124	0mm	0.975 x 1.5 x 0.975		6	24	35	FreeSurfer (5.3.0)
HUNT	HUNT	3D T1-weighted inversion recovery prepared fast spoiled gradient recalled sequence (IR-FSPGR)	1.5T GE Signa HDx	Sagittal	162	0mm	0.9375*0.9375*1.2		4.2ms	10.2ms	10	FreeSurfer (5.3.0)
IMAGEN	IMAGEN_a	ADNI MPRAGE	3T	Sagittal	170	0mm	1.1x1.1 x1.1 mm	900 ms	3,016 ms	7,16 ms	8	Freesurfer
	IMAGEN_b	ADNI MPRAGE	3T	Sagittal	160	0mm	1.1x1.1 x1.1 mm	900 ms	2,93 ms	2300 ms	9	Freesurfer
	IMAGEN_c	ADNI MPRAGE	3T	Sagittal	160	0mm	1.1x1.1 x1.1 mm	900 ms	2,93 ms	2300 ms	9	Freesurfer
	IMAGEN_d	ADNI MPRAGE	3T	Sagittal	137	0mm	1.1x1.1 x1.1 mm	900 ms	2,78 ms	6,9 ms	9	Freesurfer
	IMAGEN_e	ADNI MPRAGE	3T	Sagittal	160	0mm	1.1×1.1 ×1.1 mm	900 ms	2.93 ms	2300 ms	9	Freesurfer
	IMAGEN_f	ADNI MPRAGE	3T	Sagittal	170	0mm	1.1×1.1 ×1.1 mm	900 ms	2,81 ms	6,608 ms	8	Freesurfer
	IMAGEN_g	ADNI MPRAGE	3T	Sagittal	160	0mm	1.1×1.1 ×1.1 mm	900 ms	2.93 ms	2300 ms	9	Freesurfer
	IMAGEN_h	ADNI MPRAGE	3T	Sagittal	137	0mm	1.1×1.1 ×1.1 mm	900 ms	2,78 ms	6,9 ms	9	Freesurfer
	IMAGEN_i	ADNI MPRAGE	3T	Sagittal	160	0mm	1.1×1.1 ×1.1 mm	900 ms	2.93 ms	2300 ms	9	Freesurfer
MAS	MAS_0, MAS_1,	3D T1-weighted	3T Philips Achieva Quasar	Coronal	190	0mm	1x1x1	439	2.9	6.39	8	FreeSurfer (5.3.0)
MCIC	MCIC_MGH	Gradient Echo	1.5T Siemens	Coronal	128	0mm	0.625 × 0.625 × 1.5		4.76ms	12ms	20	FreeSurfer (5.3.0)
	MCIC_IA	Gradient Echo	1.5T GE Signa	Coronal	128	0mm	0.6641 × 0.6641 × 1.6		6ms	20ms	30	FreeSurfer (5.3.0)

	MCIC_UMN	3D T1-weighted magnetization prepared rapid acquisition gradient echo (MPRAGE)	3T Siemens Trio	Coronal	128	0mm	0.625 × 0.625 × 1.5	1100 ms	3.79ms	2530ms	7	FreeSurfer (5.3.0)
	MCIC_UNM	Gradient Echo	1.5T Siemens	Coronal	128	0mm	0.625 × 0.625 × 1.5		4.76ms	12ms	20	FreeSurfer (5.3.0)
METH-CT	METH-CT	3D T1-weighted multi-echo magnetization prepared rapid acquisition gradient echo (MEMPRAGE)	3T Siemens MAGNETOM Allegra	Sagittal	160	0.5mm	1x1x1	1100 ms	1.53ms, 3.21ms, 4.89ms, 6.57ms	2530ms	7	FreeSurfer (5.3.0)
OATS	OATS_a	3D T1-weighted volumetric	Siemens Sonata 1.5T	Coronal	144	0mm	1x1x1.5mm	780 ms	3.24	1530ms	8	FreeSurfer (5.3.0)
	OATS_b	3D T1-weighted	1.5T Siemens Magnetom Avanto	Coronal	144	0	1x1x1.5		3.24	1530	8	FreeSurfer (5.3.0)
	OATS_c	3D T1-weighted	3T Philips Achieva Quasar	Coronal	190	0	1×1×1	439	2.9	6.39	8	FreeSurfer (5.3.0)
	OATS_d	T1TFE	1.5T Philips Gyroscan	Coronal	150	0	1x1x1.5	385	3.7	7.73	8	FreeSurfer (5.3.0)
QTIM	QTIM	3D T1-weighted (MPRAGE)	4T Bruker Medspec	Coronal (85%), Sagittal (15%)	256 (coronal), 240 (sagittal)	0.45 mm	0.9375x0.9375x0.9	700 ms	3.35ms	1500ms	8	FreeSurfer (5.3)
Stroke (TOP_T3)	TOP_T3	3D gradient echo (TFE) sequence (FSPGR)	3T GE Signa HDxT	Sagittal	170	0	1x1x1,2	450	2.956	7.8	12	FreeSurfer (5.3.0)
GAP	GAP	3D T1-weighted magnetization prepared rapid acquisition gradient echo (MPRAGE)_	3T Siemens Tim Trio	Coronal	180	0mm	1.25x1.25x1.2	500 ms	4ms	500ms	8	FreeSurfer (5.1.0)
Haavik	Bergen	3D SPGR	3T GE Medical systems	Sagittal	180	1 mm	1x1x1	500 ms	3.15 ms	7.95 ms	11	FreeSurfer (5.3.0)
NCNG	NCNG1	3D T1-weighted magnetization prepared rapid acquisition gradient echo (MPRAGE)	1.5T Siemens Avanto	Sagittal	160	0mm	1.3x1.3x1.2	1000 ms	3.60ms	2400ms	8	FreeSurfer (5.3.0)
	NCNG_b	3D T1-weighted magnetization prepared rapid acquisition gradient echo (MPRAGE)	1.5T Siemens Sonata	Sagittal	128	0mm	1.0x1.0x1.33	1000 ms	3.43ms	2730ms	7	FreeSurfer (5.3.0)
NTR	NTR_1	3D T1-weighted magnetization prepared rapid acquisition gradient echo (MPRAGE)	1.5T Siemens Sonata	Sagittal	160	0mm	1.00x1.00x1.00	1100 ms	3.93ms	1900ms	15	FreeSurfer (5.1.0)
	NTR_2	3D gradient-echo T1-weighted sequence (technique: T1TFE)	3.0T Philips Intera	Coronal	182	0mm	1.00x1.00x1.20		4.60ms	9.69ms	8	FreeSurfer (5.1.0)

	NTR_3	3D T1-weighted magnetization prepared rapid acquisition gradient echo (MPRAGE)	1.5T Siemens Sonata	Coronal	160	0mm	1.00x1.00X1.50	300 ms	7.00ms	15ms	8	FreeSurfer (5.1.0)
	NTR_42	3D spoiled gradient-echo T1-weighted sequence (technique: T1FFE)	1.5T Philips GyroScan Intera	Coronal	170-180	0mm	1.00x1.00x1.20		4.6ms	30ms	30	FreeSurfer (5.3.0)
	Brainscale											
PAFIP	PAFIP	3D T1-weighted turbo field echo (TFE)	3T Philips Achieva	Sagittal	160	1	0.9375x0.9375x1		3.704ms	8.104ms	8	FreeSurfer (5.3)
SHIP	SHIP	3D T1-weighted magnetization prepared rapid acquisition gradient echo (MPRAGE)	1.5T Siemens Magnetom Avanto	Axial	176	0mm	1x1x1	1100 ms	3.4ms	1900ms	15	subcortical: FreeSurfer (5.1.0); cortical: FreeSurfer (5.3.0)

Table S5 – Number carriers that were diagnosed with any medical condition, and number of carriers that were specifically diagnosed with a neurological or psychiatric condition for each CNV in the ENIGMA-CNV dataset.

CNVs present in the study sample	N	N carriers with a medical diagnosis	N neurological or psychiatric diagnosis
1q21 TAR deletion	2	1	1
1q21 TAR duplication	9	2	1
1q21.1 BP3-BP4 deletion	19	12	3
1q21.1 BP3-BP4 duplication	10	8	1
2p16.3 (NRXN1) deletion	2	0	0
2q11.2 deletion	2	0	0
2q11.2 duplication	1	0	0
2q13 (NHP1) deletion	72	3	2
2q13 (NHP1) duplication	48	3	2
2q13 deletion	1	0	0
3q29 deletion	2	2	2
10q11.21q11.23 deletion	5	0	0
10q11.21q11.23 duplication	1	0	0
13q12.12 deletion	3	0	0
15q11.2 BP1-BP2 deletion	26	3	3
15q11.2 BP1-BP2 duplication	42	3	2
15q11q13 BP3-BP4 deletion	1	0	0

15q11q13 BP3-BP4 duplication	1	0	0
15q13.3 BP4-BP5 deletion	2	2	2
15q13.3 BP4-BP5 duplication	7	1	1
15q13.3 (CHRNA7) deletion	1	0	0
15q13.3 (CHRNA7) duplication	53	4	4
16p13.11 deletion	2	0	0
16p13.11 duplication	21	1	1
16p12.1 deletion	7	2	1
16p12.1 duplication	6	0	0
16p11.2 BP2-BP3 deletion	3	0	0
16p11.2 BP2-BP3 duplication	8	2	2
16p11.2 BP4-BP5 deletion	3	0	0
16p11.2 BP4-BP5 duplication	2	0	0
17p12 deletion	3	1	0
17p12 duplication	5	1	0
17q12 deletion	2	0	0
17q12 duplication	9	2	2
22q11 (3Mb) deletion	11	3	3
22q11 (3Mb) duplication	6	1	1
Total	398	57	34

Table S6 – Details of cohorts from ENIGMA-22q (274 carriers of the 22q11.2 (3Mb) deletion and 291 non-carriers).

Cohort	Total n	n, females	Mean age	sd age	min age	max age	References
IoP	25	15	19.6	6.08	8	28	1,2,3,4
Maastricht	59	24	29.7	9.03	18	52	5,6
Newcastle	29	16	17.4	2.75	12	22	7
Penn	90	37	17.4	3.21	10	22	8,9
SUNY	39	16	20.7	1.73	18	26	10,11,12,13,14,15,16,17
Toronto 1	25	10	42.6	7.81	30	56	18
Toronto 2	28	16	28.1	10.5	18	52	19
Davis 1	57	28	10.5	2.33	7	15	20,21

Davis 2	95	47	11.2	2.46	7	15	22,23,24,25,26
UCLA 1	44	27	14.5	5.4	6	26	27,28,29,30,31,32
UCLA 2	47	21	14.6	6.97	6	39	27,28,29,30,31,32
Utrecht	27	19	16.5	3.08	12	25	33,34

References to Table S6:

1 Gudbrandsen, Maria, et al. "The neuroanatomy of autism spectrum disorder symptomatology in 22q11. 2 deletion syndrome." Cerebral Cortex 29.8 (2019): 3655-3665.

2 Gudbrandsen, Maria, et al. "Patterns of cortical folding associated with autistic symptoms in carriers and noncarriers of the 22q11. 2 microdeletion." Cerebral Cortex 30.10 (2020): 5281-5292

3 Gudbrandsen, Maria, et al. "Neuroanatomical underpinnings of autism symptomatology in carriers and non-carriers of the 22q11. 2 microdeletion." Molecular autism 11.1 (2020): 1-15

4 Gudbrandsen, M., et al. "Brain morphometry in 22q11. 2 deletion syndrome: an exploration of differences in cortical thickness, surface area, and their contribution to cortical volume." Scientific reports 10.1 (2020): 1-12

5 Bakker, Geor, et al. "Cortical morphology differences in subjects at increased vulnerability for developing a psychotic disorder: a comparison between subjects with ultra-high risk and 22q11. 2 deletion syndrome." PLoS One 11.11 (2016): e0159928

6 da Silva Alves, Fabiana, et al. "White matter abnormalities in adults with 22q11 deletion syndrome with and without schizophrenia." Schizophrenia research 132.1 (2011): 75-83

7 McCabe, Kathryn Louise, et al. "Pre-pulse inhibition and antisaccade performance indicate impaired attention modulation of cognitive inhibition in 22q11. 2 deletion syndrome (22q11DS)." Journal of neurodevelopmental disorders 6.1 (2014): 1-8

8 Schmitt, JE, Vandekar S, Yi J, Calkins ME, Ruparel K, Roalf DR, Gur RE. Schmitt, J. Eric, et al. "Aberrant cortical morphometry in the 22q11. 2 deletion syndrome." Biological psychiatry 78.2 (2015): 135-143. Biological Psychiatry 2015; 78(2): 135–143

9 Schmitt, J. Eric, et al. "Disrupted anatomic networks in the 22q11. 2 deletion syndrome." NeuroImage: Clinical 12 (2016): 420-428

10 Radoeva, Petya D., et al. "Atlas-based white matter analysis in individuals with velo-cardio-facial syndrome (22q11. 2 deletion syndrome) and unaffected siblings." Behavioral and Brain Functions 8.1 (2012): 1-11

11 Kunwar, Arun, et al. "Cortical gyrification in velo-cardio-facial (22q11. 2 deletion) syndrome: a longitudinal study." Schizophrenia research 137.1-3 (2012): 20-25

12 Kates, Wendy R., et al. "Neuroanatomic predictors to prodromal psychosis in velocardiofacial syndrome (22q11. 2 deletion syndrome): a longitudinal study." Biological psychiatry 69.10 (2011): 945-952

13 Kates, Wendy R., et al. "Mapping cortical morphology in youth with velocardiofacial (22q11. 2 deletion) syndrome." Journal of the American Academy of Child & Adolescent Psychiatry 50.3 (2011): 272-282

14 Coman, Ioana L., et al. "The effects of gender and catechol O-methyltransferase (COMT) Val108/158Met polymorphism on emotion regulation in velo-cardio-facial syndrome (22q11. 2 deletion syndrome): an fMRI study." Neuroimage 53.3 (2010): 1043-1050

15 Antshel, Kevin M., et al. "Associations between performance on the Rey-Osterrieth Complex Figure and regional brain volumes in children with and without velocardiofacial syndrome." Developmental neuropsychology 33.5 (2008): 601-622

16 Kates, Wendy R., et al. "The neural correlates of non-spatial working memory in velocardiofacial syndrome (22q11. 2 deletion syndrome)." Neuropsychologia 45.12 (2007): 2863-2873

17 Kates, Wendy R., et al. "A gender-moderated effect of a functional COMT polymorphism on prefrontal brain morphology and function in velo-cardio-facial syndrome (22q11. 2 deletion syndrome)." American Journal of Medical Genetics Part B: Neuropsychiatric Genetics 141.3 (2006): 274-280

18 Butcher, Nancy J., et al. "Neuroimaging and clinical features in adults with a 22q11. 2 deletion at risk of Parkinson's disease." Brain 140.5 (2017): 1371-1383

19 Chow, Eva WC, et al. "Association of schizophrenia in 22q11. 2 deletion syndrome and gray matter volumetric deficits in the superior temporal gyrus." American Journal of Psychiatry 168.5 (2011): 522-529

20 DeBoer, Tracy, et al. "Hippocampal volume reduction in children with chromosome 22q11. 2 deletion syndrome is associated with cognitive impairment." Behavioral and Brain Functions 3.1 (2007): 1-9

21 Simon, Tony J., et al. "Atypical cortical connectivity and visuospatial cognitive impairments are related in children with chromosome 22q11. 2 deletion syndrome." Behavioral and Brain Functions 4.1 (2008): 1-11

22 Beaton, Elliott A., et al. "Increased incidence and size of cavum septum pellucidum in children with chromosome 22q11. 2 deletion syndrome." Psychiatry Research: Neuroimaging 181.2 (2010): 108-113

23 Srivastava, Siddharth, Michael H. Buonocore, and Tony J. Simon. "Atypical developmental trajectory of functionally significant cortical areas in children with chromosome 22q11. 2 deletion syndrome." Human brain mapping 33.1 (2012): 213-223

24 Villalon-Reina, Julio, et al. "White matter microstructural abnormalities in girls with chromosome 22q11. 2 deletion syndrome, Fragile X or Turner syndrome as evidenced by diffusion tensor imaging." Neuroimage 81 (2013): 441-454

25 Deng, Yi, et al. "Disrupted fornix integrity in children with chromosome 22q11. 2 deletion syndrome." Psychiatry Research: Neuroimaging 232.1 (2015): 106-114

26 Scott, Julia A., et al. "The hippocampi of children with chromosome 22q11. 2 deletion syndrome have localized anterior alterations that predict severity of anxiety." Journal of Psychiatry and Neuroscience 41.3 (2016): 203-213

27 Lin, Amy, et al. "Mapping 22q11. 2 gene dosage effects on brain morphometry." Journal of Neuroscience 37.26 (2017): 6183-6199

28 Jalbrzikowski, Maria, et al. "Categorical versus dimensional approaches to autism-associated intermediate phenotypes in 22q11. 2 microdeletion syndrome." Biological Psychiatry: Cognitive Neuroscience and Neuroimaging 2.1 (2017): 53-65

29 Jonas, Rachel K., et al. "Altered brain structure-function relationships underlie executive dysfunction in 22q11. 2 Deletion Syndrome." Complex Psychiatry 1.4 (2015): 235-246

30 Montojo, C. A., et al. "Neural mechanisms of response inhibition and impulsivity in 22q11. 2 deletion carriers and idiopathic attention deficit hyperactivity disorder." NeuroImage: Clinical 9 (2015): 310

31 Montojo, C. A., et al. "Disrupted working memory circuitry and psychotic symptoms in 22q11. 2 deletion syndrome." NeuroImage: Clinical 4 (2014): 392-402

32 Jalbrzikowski, Maria, et al. "Structural abnormalities in cortical volume, thickness, and surface area in 22q11. 2 microdeletion syndrome: relationship with psychotic symptoms." NeuroImage: Clinical 3 (2013): 405-415

33 Fiksinski, A. M., et al. "Neurocognition and adaptive functioning in a genetic high risk model of schizophrenia." Psychological Medicine 49.6 (2019): 1047-1054

34 Nuninga, Jasper Olivier, et al. "White matter abnormalities in 22q11. 2 deletion syndrome patients showing cognitive decline." Psychological Medicine 48.10 (2018): 1655-1663

Supplementary Results

Table S7 – Main analyses results. Linear regression results in the main sample (ENIGMA-CNV) after quality control, and after correcting for scanner site (using ComBat), sex, age, age^2 and ICV. P-values adjusted for multiple testing using FDR correction. P-values adjusted with a more conservative approach (FWE Bonferroni) are also shown. *p<0.05, **p<0.01, ***p<0.001.

		Associations with PenSZ					Associations with PenDD				
**Measure**	**regions**	**b value**	**t value**	**p value**	**p value (FDR)**	**P value (FWE)**	**b value**	**t value**	**p value**	**p value (FDR)**	**P value (FWE)**
Volume of subcortical structures	accumbens	-0.02	-0.43	0.66	0.73	1	-0.03	-0.64	0.52	0.70	1
	amygdala	-0.03	-0.55	0.58	0.71	1	-0.06	-1.13	0.26	0.43	1
	caudate	0.08	1.67	0.09	0.21	1	0.05	1.06	0.29	0.46	1
	hippocampus	-0.06	-1.10	0.27	0.44	1	-0.07	-1.48	0.14	0.31	1
	pallidum	-0.003	-0.07	0.95	0.95	1	-0.03	-0.62	0.54	0.71	1
	putamen	-0.05	-1.07	0.29	0.46	1	-0.07	-1.37	0.17	0.34	1
	thalalamus	-0.03	-0.60	0.55	0.69	1	-0.0002	-0.003	0.997	0.997	1
Cortical surface area	banks superior temporal	-0.04	-0.80	0.43	0.56	1	-0.08	-1.61	0.11	0.26	1
	caudal anterior cingulate	-0.18	-3.55	0.0004	**0.008***	**0.03***	-0.19	-3.83	0.0002	**0.007***	**0.01***
	caudal middle frontal	-0.08	-1.66	0.097	0.21	1	-0.05	-0.94	0.35	0.53	1
	**cuneus**	-0.20	-4.09	5.35E-05	**0.002***	**0.004***	-0.17	-3.30	0.001	**0.013***	0.08
	entorhinal	0.03	0.50	0.62	0.71	1	-0.0095	-0.19	0.85	0.94	1
	fusiform	-0.09	-1.71	0.09	0.21	1	-0.15	-3.05	0.002	**0.03***	0.2
	inferior parietal	-0.05	-1.04	0.30	0.47	1	-0.08	-1.66	0.097	0.24	1
	inferior temporal	-0.11	-2.20	0.03	0.11	1	-0.09	-1.86	0.06	0.18	1
	isthmus cingulate	-0.005	-0.11	0.92	0.93	1	0.05	0.94	0.35	0.53	1
	lateral occipital	-0.12	-2.42	0.02	0.11	1	-0.12	-2.31	0.02	0.10	1
	lateral orbitofrontal	-0.14	-2.86	0.004	**0.049***	0	-0.11	-2.28	0.02	0.10	1
	**lingual**	-0.20	-3.95	9.44E-05	**0.002***	**0.007***	-0.19	-3.78	0.0002	**0.007***	**0.01***
	medial orbitofrontal	-0.15	-3.06	0.002	**0.04***	0.2	-0.13	-2.58	0.01	0.07	0.8
	middle temporal	-0.02	-0.47	0.64	0.73	1	-0.07	-1.41	0.16	0.34	1
	parahippocampal	-0.05	-0.96	0.34	0.50	1	-0.05	-0.92	0.36	0.53	1
	para central	-0.05	-0.91	0.36	0.52	1	-0.03	-0.66	0.51	0.70	1
	pars opercularis	-0.11	-2.24	0.03	0.11	1	-0.09	-1.73	0.08	0.22	1
	pars orbitalis	-0.09	-1.71	0.09	0.21	1	-0.15	-2.97	0.003	**0.03***	0.2

	pars triangularis	-0.10	-2.02	0.04	0.13	1	-0.13	-2.61	0.009	0.07	0.7
	pericalcarine	-0.15	-2.99	0.003	**0.04***	0.2	-0.13	-2.49	0.01	0.09	1
	post central	-0.08	-1.54	0.12	0.25	1	-0.14	-2.82	0.005	**0.04***	0.4
	posterior cingulate	-0.06	-1.17	0.24	0.40	1	-0.10	-2.05	0.04	0.16	1
	pre central	-0.01	-0.28	0.78	0.82	1	-0.006	-0.12	0.90	0.94	1
	precuneus	-0.12	-2.38	0.02	0.11	1	-0.10	-2.01	0.04	0.16	1
	rostral anterior cingulate	-0.11	-2.15	0.03	0.11	1	-0.11	-2.23	0.03	0.11	1
	rostral middle frontal	-0.09	-1.78	0.08	0.20	1	-0.12	-2.45	0.01	0.09	1
	superior frontal	-0.08	-1.67	0.096	0.21	1	-0.07	-1.30	0.19	0.35	1
	superior parietal	-0.098	-1.95	0.05	0.14	1	-0.12	-2.35	0.02	0.099	1
	superior temporal	-0.12	-2.40	0.02	0.11	1	-0.17	-3.33	0.0009	**0.013***	0.07
	supramarginal	-0.03	-0.50	0.61	0.71	1	-0.06	-1.12	0.26	0.43	1
	frontal pole	-0.07	-1.37	0.17	0.33	1	-0.10	-1.89	0.06	0.17	1
	temporal pole	-0.06	-1.27	0.20	0.36	1	0.003	0.06	0.95	0.98	1
	transverse temporal	-0.07	-1.35	0.18	0.34	1	-0.06	-1.28	0.20	0.35	1
	insula	-0.01	-0.24	0.81	0.85	1	-0.01	-0.20	0.84	0.94	1
	**Total Surface Area**	-0.14	-2.72	0.007	**0.07.**	0.5	-0.17	-3.39	0.0008	**0.013***	0.06
Cortical Thickness	banks superior temporal	0.09	1.74	0.08	0.21	1	0.098	1.94	0.05	0.17	1
	caudal anterior cingulate	0.03	0.57	0.57	0.70	1	0.02	0.47	0.64	0.73	1
	caudal middle frontal	0.04	0.86	0.39	0.54	1	0.02	0.47	0.64	0.73	1
	**cuneus**	0.03	0.61	0.54	0.69	1	0.02	0.44	0.66	0.74	1
	entorhinal	-0.06	-1.22	0.22	0.38	1	-0.02	-0.48	0.63	0.73	1
	fusiform	0.05	0.95	0.34	0.50	1	0.03	0.57	0.57	0.71	1
	inferior parietal	0.06	1.21	0.23	0.38	1	0.09	1.73	0.08	0.22	1
	inferior temporal	0.08	1.65	0.10	0.21	1	0.12	2.40	0.02	0.09	1
	isthmus cingulate	-0.03	-0.62	0.53	0.69	1	-0.07	-1.32	0.19	0.35	1
	lateral occipital	0.04	0.82	0.41	0.56	1	0.03	0.61	0.54	0.71	1
	lateral orbitofrontal	0.10	2.04	0.04	0.13	1	0.07	1.47	0.14	0.31	1
	**lingual**	0.12	2.30	0.02	0.11	1	0.07	1.31	0.19	0.35	1
	medial orbitofrontal	0.12	2.43	0.02	0.11	1	0.06	1.18	0.24	0.41	1
	middle temporal	0.11	2.25	0.02	0.11	1	0.10	2.00	0.05	0.16	1
	parahippocampal	-0.04	-0.87	0.38	0.54	1	-0.03	-0.54	0.59	0.72	1
	para central	0.03	0.51	0.61	0.71	1	0.05	0.92	0.36	0.53	1
	pars opercularis	0.11	2.27	0.02	0.11	1	0.07	1.36	0.17	0.34	1
	pars orbitalis	0.11	2.23	0.03	0.11	1	0.097	1.92	0.06	0.17	1

	pars triangularis	0.12	2.33	0.02	0.11	1	0.12	2.33	0.02	0.10	1
	pericalcarine	-0.007	-0.14	0.89	0.92	1	0.002	0.04	0.97	0.98	1
	post central	0.07	1.32	0.19	0.34	1	0.04	0.81	0.42	0.59	1
	posterior cingulate	0.02	0.40	0.69	0.73	1	-0.007	-0.14	0.89	0.94	1
	pre central	-0.05	-1.01	0.31	0.48	1	-0.02	-0.48	0.63	0.73	1
	precuneus	0.11	2.13	0.03	0.11	1	0.07	1.39	0.17	0.34	1
	rostral anterior cingulate	0.02	0.42	0.68	0.73	1	0.007	0.13	0.90	0.94	1
	rostral middle frontal	0.13	2.67	0.008	0.07	0.6	0.07	1.47	0.14	0.31	1
	superior frontal	0.07	1.34	0.18	0.34	1	0.03	0.60	0.55	0.71	1
	superior parietal	0.10	2.05	0.04	0.13	1	0.098	1.93	0.05	0.17	1
	superior temporal	0.02	0.43	0.67	0.73	1	0.04	0.75	0.45	0.63	1
	supramarginal	0.08	1.57	0.12	0.24	1	0.06	1.22	0.22	0.39	1
	frontal pole	0.11	2.16	0.03	0.11	1	0.10	2.01	0.04	0.16	1
	temporal pole	0.04	0.80	0.42	0.56	1	0.03	0.57	0.57	0.71	1
	transverse temporal	-0.03	-0.52	0.61	0.71	1	0.008	0.15	0.88	0.94	1
	insula	0.11	2.09	0.04	0.12	1	0.05	0.90	0.37	0.53	1
	**Mean Thickness**	0.09	1.77	0.08	0.20	1	0.08	1.49	0.14	0.31	1
Intracranial volume	**ICV**	-0.24	-5.01	8.10E-07	**6.31E-05*** **	**6.31E-05*** **	-0.18	-3.56	0.0004	**0.011***	**0.03***

Table S8 – Comparisons between CNV carriers and non-carrier controls, after correcting for scanner site (using ComBat), sex, age, age^2 and ICV . *p<0.05, **p<0.01, ***p<0.001.

		**A) including all CNVs in ENIGMA-CNV sample**			**B) including only CNVs (1q21.1 distal deletion, 1q21.1 distal duplication, 2p16.3 (NRXN1) deletion, 15q11.2 BP1-BP2 deletion, 16p13.11 duplication and 16p12.1 deletion) in Caseras et al. study [1]**		
**Measure**	**regions**	**t value**	**p value**	**p value (FDR)**	**t value**	**p value**	**p value (FDR)**
Volume of subcortical structures	accumbens	-0.78	0.44	0.72	-1.74	0.08	0.22
	amygdala	-2.79	0.005	0.06	-2.25	0.02	0.11
	caudate	-0.99	0.32	0.71	-0.59	0.56	0.71
	hippocampus	-1.17	0.24	0.70	-1.12	0.26	0.44

	pallidum	-0.28	0.78	0.89	0.28	0.78	0.85
	putamen	-3.05	0.002	**0.04***	-1.71	0.09	0.22
	thalalamus	-1.76	0.08	0.38	-0.49	0.62	0.76
Cortical surface area	banks superior temporal	0.04	0.97	0.98	0.53	0.59	0.73
	caudal anterior cingulate	-2.38	0.02	0.13	-2.49	0.01	0.08
	caudal middle frontal	0.27	0.79	0.89	-1.64	0.10	0.24
	**cuneus**	-0.13	0.89	0.97	-1.30	0.20	0.37
	entorhinal	-1.53	0.13	0.47	-0.93	0.35	0.54
	fusiform	-2.71	0.007	0.07	-0.35	0.73	0.83
	inferior parietal	-2.44	0.01	0.13	-2.09	0.04	0.15
	inferior temporal	-1.02	0.31	0.71	-1.97	0.05	0.17
	isthmus cingulate	0.51	0.61	0.81	0.28	0.78	0.85
	lateral occipital	-3.98	6.81E-05	**0.005****	-1.33	0.18	0.36
	lateral orbitofrontal	0.03	0.98	0.98	-2.76	0.006	**0.04***
	**lingual**	-0.93	0.35	0.72	-0.43	0.67	0.80
	medial orbitofrontal	-0.03	0.97	0.98	-1.27	0.20	0.37
	middle temporal	-0.33	0.74	0.89	0.73	0.46	0.64
	parahippocampal	-0.56	0.57	0.80	-0.32	0.75	0.83
	para central	-0.71	0.48	0.72	-1.96	0.05	0.17
	pars opercularis	-0.16	0.87	0.96	-0.59	0.56	0.71
	pars orbitalis	-0.23	0.82	0.91	-1.28	0.20	0.37
	pars triangularis	-0.27	0.79	0.89	-0.16	0.87	0.88
	pericalcarine	1.03	0.30	0.71	0.23	0.82	0.85
	post central	-1.12	0.26	0.70	-0.42	0.68	0.80
	posterior cingulate	-0.44	0.66	0.85	-0.24	0.81	0.85
	pre central	-2.91	0.004	**0.047***	-2.38	0.02	0.09
	precuneus	-1.72	0.08	0.38	-1.80	0.07	0.21
	rostral anterior cingulate	-0.85	0.40	0.72	-2.05	0.04	0.16
	rostral middle frontal	-2.04	0.04	0.23	-0.80	0.42	0.62
	superior frontal	-2.34	0.02	0.14	-2.08	0.04	0.15
	superior parietal	-0.27	0.79	0.89	0.26	0.79	0.85
	superior temporal	-0.82	0.41	0.72	0.35	0.73	0.83
	supramarginal	-0.87	0.38	0.72	1.10	0.27	0.44
	frontal pole	0.46	0.65	0.84	1.34	0.18	0.36
	temporal pole	-3.19	0.001	**0.04***	-3.07	0.002	**0.03***

	transverse temporal	0.57	0.57	0.80	0.85	0.40	0.60
	insula	0.54	0.59	0.80	-0.16	0.87	0.88
	**Total Surface Area**	-2.23	0.03	0.17	-1.21	0.23	0.40
Cortical Thickness	banks superior temporal	-0.42	0.68	0.85	1.50	0.13	0.30
	caudal anterior cingulate	-0.10	0.92	0.98	1.82	0.07	0.21
	caudal middle frontal	-1.15	0.25	0.70	1.98	0.05	0.17
	**cuneus**	-1.98	0.05	0.25	-0.74	0.46	0.64
	entorhinal	0.69	0.49	0.72	-0.55	0.58	0.73
	fusiform	-1.49	0.14	0.48	0.60	0.55	0.71
	inferior parietal	0.02	0.98	0.98	1.70	0.09	0.22
	inferior temporal	0.71	0.48	0.72	2.41	0.02	0.09
	isthmus cingulate	1.29	0.20	0.64	2.52	0.01	0.08
	lateral occipital	-0.98	0.33	0.71	0.13	0.90	0.90
	lateral orbitofrontal	-0.53	0.59	0.80	3.30	0.00098	**0.02***
	**lingual**	-1.59	0.11	0.46	1.02	0.31	0.49
	medial orbitofrontal	-0.79	0.43	0.72	3.63	0.0003	**0.01***
	middle temporal	0.37	0.71	0.88	2.85	0.004	**0.04***
	parahippocampal	-3.10	0.002	**0.04***	-1.72	0.09	0.22
	para central	-0.91	0.36	0.72	-0.34	0.73	0.83
	pars opercularis	-0.70	0.49	0.72	3.18	0.001	**0.02***
	pars orbitalis	-0.70	0.48	0.72	1.36	0.17	0.36
	pars triangularis	-0.84	0.40	0.72	1.86	0.06	0.19
	pericalcarine	-0.99	0.32	0.71	-0.72	0.47	0.65
	post central	-1.54	0.12	0.47	0.78	0.43	0.63
	posterior cingulate	-1.08	0.28	0.70	0.95	0.34	0.53
	pre central	-0.87	0.39	0.72	1.14	0.25	0.43
	precuneus	-0.69	0.49	0.72	1.94	0.05	0.17
	rostral anterior cingulate	-1.71	0.09	0.38	1.48	0.14	0.30
	rostral middle frontal	-1.10	0.27	0.70	2.90	0.004	**0.04***
	superior frontal	-0.28	0.78	0.89	3.37	0.0007	**0.02***
	superior parietal	0.02	0.98	0.98	2.83	0.005	**0.04***
	superior temporal	-1.17	0.24	0.70	1.36	0.17	0.36
	supramarginal	-0.59	0.56	0.80	1.14	0.25	0.43
	frontal pole	-3.02	0.003	**0.04***	1.70	0.09	0.22
	temporal pole	-0.79	0.43	0.72	2.44	0.01	0.09

	transverse temporal	-1.39	0.16	0.55	-0.69	0.49	0.66
	insula	-1.11	0.27	0.70	1.51	0.13	0.30
	**Mean Thickness**	-0.94	0.35	0.72	2.36	0.02	0.09
Intracranial volume	**ICV**	-2.16	0.03	0.19	-3.79	0.0002	**0.01***

[1] Caseras, X. et al. Effects of genomic copy number variants penetrant for schizophrenia on cortical thickness and surface area in healthy individuals: analysis of the UK Biobank. The British Journal of Psychiatry 218, 104–111 (2021).

Table S9 – Linear regression results including additional data from the ENIGMA-22q dataset (n=274 22q11.2 deletion carriers and n=291 non-carriers).

		Associations with PenSZ				Associations with PenDD			
**Measure**	**regions**	**b value**	**t value**	**p value**	**p value (FDR)**	**b value**	**t value**	**p value**	**p value (FDR)**
Volume of subcortical structures	accumbens	0.21	5.64	2.57E-08	**1.06E-07 (+)**	0.21	5.56	3.96E-08	**1.71E-07 (+)**
	amygdala	0.004	0.11	0.91	0.92	-0.006	-0.14	0.89	0.91
	caudate	0.26	6.83	1.93E-11	**1.67E-10 (+)**	0.25	6.58	9.67E-11	**7.54E-10 (+)**
	hippocampus	-0.21	-5.47	6.54E-08	**2.43E-07 (+)**	-0.21	-5.65	2.41E-08	**1.18E-07 (+)**
	pallidum	-0.02	-0.60	0.55	0.62	-0.03	-0.81	0.42	0.47
	putamen	-0.06	-1.61	0.11	0.14	-0.07	-1.74	0.08	0.11
	thalalamus	0.02	0.59	0.55	0.62	0.03	0.84	0.40	0.45
Cortical surface area	banks superior temporal	-0.10	-2.70	0.007	**0.01 (+)**	-0.12	-3.04	0.002	**0.004 (+)**
	caudal anterior cingulate	-0.32	-8.63	4.47E-17	**6.98E-16 (=)**	-0.32	-8.78	1.43E-17	**2.23E-16 (=)**
	caudal middle frontal	-0.06	-1.47	0.14	0.17	-0.05	-1.17	0.24	0.29
	**cuneus**	-0.44	-12.41	6.61E-32	**5.16E-30 (=)**	-0.43	-12.02	3.23E-30	**2.52E-28 (=)**
	entorhinal	-0.10	-2.66	0.008	**0.01 (+)**	-0.12	-2.96	0.003	**0.005 (+)**
	fusiform	-0.26	-6.77	2.79E-11	**2.18E-10 (+)**	-0.28	-7.39	4.61E-13	**4.49E-12 (=)**
	inferior parietal	-0.03	-0.89	0.37	0.43	-0.05	-1.15	0.25	0.29
	inferior temporal	-0.25	-6.58	9.75E-11	**6.91E-10 (+)**	-0.24	-6.44	2.29E-10	**1.49E-09 (+)**
	isthmus cingulate	-0.01	-0.33	0.74	0.79	0.005	0.12	0.90	0.91
	lateral occipital	-0.22	-5.67	2.11E-08	**9.66E-08 (+)**	-0.21	-5.63	2.67E-08	**1.23E-07 (+)**
	lateral orbitofrontal	-0.08	-2.04	0.04	**0.06 (-)**	-0.07	-1.81	0.07	0.09
	**lingual**	-0.40	-11.29	3.77E-27	**1.47E-25 (=)**	-0.40	-11.23	6.53E-27	**2.55E-25 (=)**
	medial orbitofrontal	-0.08	-2.16	0.03	**0.046 (=)**	-0.08	-1.97	0.05	0.07

	middle temporal	-0.14	-3.56	0.0004	**0.0008 (+)**	-0.15	-3.96	8.24E-05	**0.0002 (+)**
	parahippocampal	0.01	0.27	0.79	0.82	0.01	0.28	0.78	0.85
	para central	-0.16	-4.15	3.78E-05	**8.92E-05 (+)**	-0.16	-4.04	6.10E-05	**0.0001 (+)**
	pars opercularis	-0.05	-1.19	0.23	0.27	-0.04	-0.99	0.32	0.37
	pars orbitalis	-0.09	-2.32	0.02	**0.03 (+)**	-0.11	-2.83	0.005	**0.008 (=)**
	pars triangularis	-0.22	-5.71	1.71E-08	**8.32E-08 (+)**	-0.23	-5.98	3.68E-09	**2.05E-08 (+)**
	pericalcarine	-0.36	-9.74	5.04E-21	**1.12E-19 (=)**	-0.35	-9.51	3.58E-20	**6.98E-19 (+)**
	post central	-0.22	-5.79	1.07E-08	**5.58E-08 (+)**	-0.24	-6.39	3.20E-10	**1.92E-09 (=)**
	posterior cingulate	-0.14	-3.52	0.0005	**0.0009 (+)**	-0.15	-3.91	0.0001	**0.0002 (+)**
	pre central	0.13	3.34	0.0009	**0.002 (+)**	0.13	3.41	0.0007	**0.001 (+)**
	precuneus	-0.36	-9.72	5.76E-21	**1.12E-19 (+)**	-0.35	-9.56	2.41E-20	**6.26E-19 (+)**
	rostral anterior cingulate	-0.20	-5.17	3.08E-07	**1.05E-06 (+)**	-0.20	-5.21	2.47E-07	**8.76E-07 (+)**
	rostral middle frontal	-0.26	-6.94	9.65E-12	**9.41E-11 (+)**	-0.27	-7.24	1.29E-12	**1.12E-11 (+)**
	superior frontal	-0.07	-1.68	0.09	0.12	-0.06	-1.53	0.13	0.16
	superior parietal	-0.31	-8.43	2.24E-16	**2.91E-15 (+)**	-0.32	-8.61	5.42E-17	**7.05E-16 (+)**
	superior temporal	-0.12	-3.02	0.003	**0.005 (+)**	-0.13	-3.38	0.0008	**0.001 (=)**
	supramarginal	-0.05	-1.27	0.21	0.25	-0.06	-1.51	0.13	0.16
	frontal pole	-0.07	-1.83	0.07	0.09	-0.08	-2.05	0.04	0.06
	temporal pole	-0.16	-4.25	2.40E-05	**5.84E-05 (+)**	-0.14	-3.68	0.0003	**5.57E-04 (+)**
	transverse temporal	-0.14	-3.64	0.0003	**0.0006 (+)**	-0.14	-3.61	0.0003	**0.0007 (+)**
	insula	0.14	3.56	0.0004	**0.0008 (+)**	0.14	3.58	0.0004	**0.0008 (+)**
	**Total Surface Area**	-0.28	-7.48	2.42E-13	**2.70E-12 (+)**	-0.29	-7.76	3.25E-14	**3.62E-13 (=)**
Cortical Thickness	banks superior temporal	0.007	0.19	0.85	0.88	0.01	0.27	0.79	0.85
	caudal anterior cingulate	-0.08	-2.18	0.03	**0.04 (+)**	-0.09	-2.22	0.03	**0.04 (+)**
	caudal middle frontal	0.20	5.12	4.06E-07	**1.32E-06 (+)**	0.19	4.95	9.25E-07	**3.01E-06 (+)**
	**cuneus**	0.17	4.49	8.48E-06	**2.28E-05 (+)**	0.17	4.44	1.07E-05	**2.87E-05 (+)**
	entorhinal	-0.002	-0.04	0.97	0.97	0.0098	0.25	0.80	0.85
	fusiform	0.08	2.03	0.04	0.06	0.07	1.87	0.06	0.09
	inferior parietal	0.06	1.60	0.11	0.14	0.07	1.83	0.07	0.09
	inferior temporal	0.07	1.91	0.06	0.08	0.09	2.22	0.03	**0.04 (+)**
	isthmus cingulate	-0.03	-0.88	0.38	0.44	-0.05	-1.18	0.24	0.29
	lateral occipital	0.07	1.75	0.08	0.11	0.06	1.66	0.097	0.12
	lateral orbitofrontal	0.13	3.26	0.001	**0.002 (+)**	0.12	3.02	0.003	**0.004 (+)**
	**lingual**	0.19	4.95	9.26E-07	**2.89E-06 (+)**	0.17	4.55	6.52E-06	**1.96E-05 (+)**
	medial orbitofrontal	0.18	4.77	2.26E-06	**6.54E-06 (+)**	0.16	4.24	2.58E-05	**6.50E-05 (+)**

	middle temporal	0.13	3.27	0.001	**0.002 (+)**	0.12	3.17	0.002	**0.003 (+)**
	parahippocampal	-0.13	-3.36	0.0008	**0.002 (+)**	-0.12	-3.22	0.001	**0.002 (+)**
	para central	0.17	4.34	1.69E-05	**4.24E-05 (+)**	0.17	4.53	6.92E-06	**2.00E-05 (+)**
	pars opercularis	0.18	4.72	2.90E-06	**8.08E-06 (+)**	0.17	4.34	1.67E-05	**4.33E-05 (+)**
	pars orbitalis	0.13	3.23	0.001	**0.002 (+)**	0.12	3.11	0.002	**0.003 (+)**
	pars triangularis	0.17	4.45	1.02E-05	**2.66E-05 (+)**	0.17	4.45	9.87E-06	**2.75E-05 (+)**
	pericalcarine	0.24	6.42	2.67E-10	**1.73E-09 (+)**	0.25	6.52	1.38E-10	**9.81E-10 (+)**
	post central	0.22	5.66	2.30E-08	**9.97E-08 (+)**	0.21	5.47	6.58E-08	**2.70E-07 (+)**
	posterior cingulate	-0.06	-1.53	0.13	0.16	-0.07	-1.76	0.08	0.10
	pre central	0.15	3.81	0.0001	**0.0003 (+)**	0.16	4.04	5.95E-05	**0.0001 (+)**
	precuneus	0.14	3.66	0.0003	**0.0006 (+)**	0.13	3.36	0.0008	**0.002 (+)**
	rostral anterior cingulate	0.01	0.28	0.78	0.82	0.007	0.17	0.87	0.90
	rostral middle frontal	0.23	6.17	1.21E-09	**7.24E-09 (+)**	0.22	5.67	2.13E-08	**1.11E-07 (+)**
	superior frontal	0.13	3.25	0.001	**0.002 (+)**	0.11	2.94	0.003	**0.005 (+)**
	superior parietal	0.11	2.86	0.004	**0.007 (+)**	0.11	2.82	0.005	**0.008 (+)**
	superior temporal	-0.08	-1.97	0.05	0.07	-0.07	-1.84	0.07	0.09
	supramarginal	0.21	5.40	9.20E-08	**3.26E-07 (+)**	0.20	5.26	1.99E-07	**7.38E-07 (+)**
	frontal pole	0.09	2.42	0.02	**0.02 (+)**	0.09	2.37	0.02	**0.03 (+)**
	temporal pole	0.01	0.34	0.73	0.79	0.009	0.24	0.81	0.85
	transverse temporal	-0.01	-0.37	0.71	0.78	-0.003	-0.09	0.93	0.93
	insula	0.22	5.91	5.44E-09	**3.03E-08 (+)**	0.21	5.39	9.91E-08	**3.86E-07 (+)**
	**Mean Thickness**	0.18	4.78	2.15E-06	**6.46E-06 (+)**	0.18	4.67	3.64E-06	**1.14E-05 (+)**
Intracranial volume	**ICV**	-0.21	-5.52	4.74E-08	**1.85E-07 (=)**	-0.19	-4.96	9.04E-07	**3.01E-06 (=)**

Table S10 – Linear regression results after excluding CNV carriers younger than 18 years old. (=) significant in main analysis and after exclusion; (+) significant after exclusion but not in main analysis; (-) not significant after exclusion but significant in main analysis.

		**Associations with PenSZ**				**Associations with PenDD**			
**Measure**	**regions**	**b value**	**t value**	**p value**	**p value (FDR)**	**b value**	**t value**	**p value**	**p value (FDR)**
	accumbens	-0.006	-0.11	0.91	0.92	-0.03	-0.58	0.56	0.88
	amygdala	-0.05	-0.80	0.42	0.65	-0.06	-1.09	0.28	0.58

Volume of subcortical structures	caudate	0.02	0.29	0.77	0.86	-0.03	-0.60	0.55	0.87
	hippocampus	-0.05	-0.83	0.41	0.65	-0.07	-1.32	0.19	0.47
	pallidum	-0.03	-0.53	0.60	0.77	-0.03	-0.51	0.61	0.88
	putamen	-0.05	-0.89	0.37	0.62	-0.05	-0.80	0.42	0.76
	thalalamus	-0.03	-0.46	0.65	0.79	0.02	0.41	0.69	0.88
Cortical surface area	banks superior temporal	-0.03	-0.60	0.55	0.74	-0.07	-1.30	0.20	0.47
	caudal anterior cingulate	-0.16	-2.96	0.003	**0.06 (-)**	-0.19	-3.39	0.0008	**0.06 (-)**
	caudal middle frontal	-0.02	-0.34	0.74	0.86	-0.009	-0.15	0.88	0.96
	**cuneus**	-0.17	-3.05	0.002	**0.06 (-)**	-0.14	-2.49	0.01	**0.09 (-)**
	entorhinal	-0.0097	-0.17	0.86	0.90	-0.07	-1.17	0.24	0.54
	fusiform	-0.09	-1.64	0.10	0.32	-0.14	-2.49	0.01	**0.09 (-)**
	inferior parietal	-0.12	-2.19	0.03	0.16	-0.12	-2.19	0.03	0.15
	inferior temporal	-0.14	-2.57	0.01	0.11	-0.12	-2.08	0.04	0.15
	isthmus cingulate	-0.04	-0.66	0.51	0.71	0.02	0.37	0.71	0.88
	lateral occipital	-0.10	-1.83	0.07	0.29	-0.10	-1.80	0.07	0.27
	lateral orbitofrontal	-0.16	-2.88	0.004	**0.07 (-)**	-0.15	-2.70	0.007	0.08
	**lingual**	-0.16	-2.80	0.005	**0.07 (-)**	-0.16	-2.80	0.005	**0.07 (-)**
	medial orbitofrontal	-0.17	-3.11	0.002	**0.06 (-)**	-0.14	-2.51	0.01	0.09
	middle temporal	-0.07	-1.19	0.23	0.48	-0.08	-1.45	0.15	0.38
	parahippocampal	-0.09	-1.56	0.12	0.34	-0.12	-2.13	0.03	0.15
	para central	0.01	0.23	0.82	0.88	0.009998	0.18	0.86	0.96
	pars opercularis	-0.09	-1.67	0.095	0.32	-0.09	-1.55	0.12	0.35
	pars orbitalis	-0.11	-1.87	0.06	0.29	-0.16	-2.88	0.004	**0.07 (-)**
	pars triangularis	-0.13	-2.27	0.02	0.14	-0.17	-3.07	0.002	0.06
	pericalcarine	-0.14	-2.50	0.01	**0.11 (-)**	-0.13	-2.37	0.02	0.11
	post central	-0.07	-1.29	0.20	0.47	-0.14	-2.51	0.01	**0.09 (-)**
	posterior cingulate	-0.07	-1.24	0.22	0.48	-0.099	-1.76	0.08	0.27
	pre central	0.002	0.03	0.98	0.98	0.002	0.03	0.98	0.98
	precuneus	-0.14	-2.43	0.02	0.11	-0.12	-2.13	0.03	0.15
	rostral anterior cingulate	-0.10	-1.85	0.07	0.29	-0.12	-2.08	0.04	0.15
	rostral middle frontal	-0.099	-1.76	0.08	0.31	-0.13	-2.38	0.02	0.11
	superior frontal	-0.09	-1.57	0.12	0.34	-0.09	-1.54	0.13	0.35
	superior parietal	-0.096	-1.70	0.09	0.32	-0.10	-1.78	0.08	0.27
	superior temporal	-0.13	-2.39	0.02	0.11	-0.16	-2.78	0.006	**0.07 (-)**
	supramarginal	-0.05	-0.93	0.35	0.62	-0.09	-1.51	0.13	0.35

	frontal pole	-0.07	-1.26	0.21	0.48	-0.12	-2.09	0.04	0.15
	temporal pole	-0.03	-0.60	0.55	0.74	0.02	0.27	0.78	0.94
	transverse temporal	-0.05	-0.93	0.35	0.62	-0.09	-1.54	0.13	0.35
	insula	-0.05	-0.80	0.42	0.65	-0.04	-0.80	0.43	0.76
	**Total Surface Area**	-0.14	-2.42	0.02	0.11	-0.17	-3.08	0.002	**0.06 (-)**
Cortical Thickness	banks superior temporal	0.05	0.96	0.34	0.62	0.05	0.91	0.36	0.71
	caudal anterior cingulate	0.03	0.47	0.64	0.79	0.008	0.14	0.89	0.96
	caudal middle frontal	0.02	0.42	0.67	0.81	-0.04	-0.64	0.52	0.86
	**cuneus**	0.00999	0.18	0.86	0.90	-0.009	-0.16	0.87	0.96
	entorhinal	-0.04	-0.70	0.49	0.70	-0.01	-0.20	0.84	0.96
	fusiform	0.06	1.08	0.28	0.55	-0.007	-0.13	0.90	0.96
	inferior parietal	0.05	0.92	0.36	0.62	0.05	0.83	0.41	0.76
	inferior temporal	0.09	1.68	0.09	0.32	0.09	1.65	0.10	0.31
	isthmus cingulate	-0.02	-0.39	0.69	0.82	-0.07	-1.29	0.20	0.47
	lateral occipital	0.06	1.15	0.25	0.50	0.02	0.44	0.66	0.88
	lateral orbitofrontal	0.08	1.40	0.16	0.42	0.04	0.74	0.46	0.79
	**lingual**	0.09	1.65	0.10	0.32	0.03	0.49	0.62	0.88
	medial orbitofrontal	0.14	2.46	0.01	0.11	0.07	1.24	0.22	0.50
	middle temporal	0.0998	1.78	0.08	0.31	0.06	1.02	0.31	0.62
	parahippocampal	-0.02	-0.27	0.79	0.86	-0.02	-0.30	0.76	0.93
	para central	-0.03	-0.54	0.59	0.77	-0.03	-0.54	0.59	0.88
	pars opercularis	0.06	1.04	0.30	0.57	0.003	0.05	0.96	0.97
	pars orbitalis	0.09	1.60	0.11	0.33	0.06	1.12	0.26	0.57
	pars triangularis	0.08	1.34	0.18	0.44	0.06	1.05	0.30	0.61
	pericalcarine	-0.02	-0.32	0.75	0.86	-0.006	-0.11	0.91	0.96
	post central	0.05	0.87	0.38	0.62	0.008	0.15	0.88	0.96
	posterior cingulate	0.008	0.13	0.89	0.92	-0.03	-0.47	0.64	0.88
	pre central	-0.11	-1.93	0.05	0.28	-0.097	-1.73	0.08	0.27
	precuneus	0.08	1.35	0.18	0.44	0.02	0.39	0.69	0.88
	rostral anterior cingulate	0.03	0.58	0.56	0.74	0.02	0.37	0.71	0.88
	rostral middle frontal	0.07	1.23	0.22	0.48	-0.02	-0.42	0.68	0.88
	superior frontal	0.03	0.46	0.65	0.79	-0.04	-0.69	0.49	0.83
	superior parietal	0.07	1.21	0.23	0.48	0.04	0.63	0.53	0.86
	superior temporal	-0.02	-0.28	0.78	0.86	-0.01	-0.23	0.82	0.96
	supramarginal	0.04	0.73	0.47	0.69	0.004	0.08	0.94	0.96

	frontal pole	0.09	1.52	0.13	0.35	0.05	0.89	0.37	0.71
	temporal pole	0.0099	0.17	0.86	0.90	-0.03	-0.52	0.61	0.88
	transverse temporal	-0.04	-0.74	0.46	0.69	-0.02	-0.37	0.71	0.88
	insula	0.04	0.68	0.50	0.71	-0.02	-0.39	0.69	0.88
	**Mean Thickness**	0.05	0.88	0.38	0.62	0.005	0.096	0.92	0.96
Intracranial volume	**ICV**	-0.19	-3.42	0.0007	**0.056 (-)**	-0.13	-2.31	0.02	**0.12 (-)**

Table S11 – Linear regression results after excluding individuals with neurodevelopment and neuropsychiatric conditions. (=) significant in main analysis and after exclusion; (+) significant after exclusion but not in main analysis; (-) not significant after exclusion but significant in main analysis.

		Associations with PenSZ				Associations with PenDD			
**Measure**	**regions**	**b value**	**t value**	**p value**	**p value (FDR)**	**b value**	**t value**	**p value**	**p value (FDR)**
Volume of subcortical structures	accumbens	-0.08	-1.58	0.12	0.43	-0.06	-1.17	0.24	0.51
	amygdala	-0.05	-0.87	0.39	0.68	-0.08	-1.54	0.12	0.36
	caudate	0.003	0.06	0.96	0.99	-0.04	-0.72	0.47	0.72
	hippocampus	-0.08	-1.42	0.16	0.43	-0.11	-2.09	0.04	0.18
	pallidum	-0.005	-0.09	0.93	0.99	-0.06	-1.20	0.23	0.50
	putamen	-0.04	-0.80	0.42	0.70	-0.07	-1.23	0.22	0.49
	thalalamus	-0.03	-0.52	0.60	0.77	0.01	0.25	0.80	0.89
Cortical surface area	banks superior temporal	-0.06	-1.09	0.28	0.54	-0.098	-1.83	0.07	0.25
	caudal anterior cingulate	-0.14	-2.61	0.0096	**0.12 (-)**	-0.16	-2.95	0.003	**0.048 (=)**
	caudal middle frontal	-0.07	-1.37	0.17	0.45	-0.04	-0.72	0.47	0.72
	**cuneus**	-0.21	-3.94	9.94E-05	**0.004 (=)**	-0.15	-2.92	0.004	**0.048 (=)**
	entorhinal	0.08	1.41	0.16	0.43	0.0095	0.18	0.86	0.93
	fusiform	-0.04	-0.69	0.49	0.70	-0.14	-2.63	0.009	**0.09 (-)**
	inferior parietal	-0.08	-1.52	0.13	0.43	-0.12	-2.24	0.03	0.15
	inferior temporal	-0.11	-2.03	0.04	0.26	-0.098	-1.83	0.07	0.25
	isthmus cingulate	-0.02	-0.40	0.69	0.83	0.05	0.89	0.37	0.66

	lateral occipital	-0.12	-2.22	0.03	0.21	-0.11	-2.09	0.04	0.18
	lateral orbitofrontal	-0.15	-2.83	0.005	**0.08 (-)**	-0.10	-1.90	0.06	0.25
	**lingual**	-0.17	-3.27	0.001	**0.03 (=)**	-0.16	-3.03	0.003	**0.048 (=)**
	medial orbitofrontal	-0.11	-2.05	0.04	**0.26 (-)**	-0.09	-1.69	0.09	0.29
	middle temporal	0.01	0.19	0.85	0.93	-0.06	-1.15	0.25	0.51
	parahippocampal	-0.01	-0.22	0.83	0.92	-0.02	-0.44	0.66	0.80
	para central	0.02	0.40	0.69	0.83	0.03	0.55	0.58	0.77
	pars opercularis	-0.12	-2.28	0.02	0.20	-0.08	-1.51	0.13	0.37
	pars orbitalis	-0.06	-1.18	0.24	0.51	-0.14	-2.69	0.008	**0.08 (-)**
	pars triangularis	-0.09	-1.72	0.09	0.43	-0.12	-2.32	0.02	0.14
	pericalcarine	-0.17	-3.14	0.002	**0.04 (=)**	-0.12	-2.35	0.02	0.14
	post central	-0.04	-0.71	0.48	0.70	-0.13	-2.35	0.02	**0.14 (-)**
	posterior cingulate	-0.00099	-0.02	0.99	0.99	-0.08	-1.41	0.16	0.43
	pre central	-0.03	-0.56	0.57	0.75	-0.02	-0.34	0.74	0.87
	precuneus	-0.08	-1.51	0.13	0.43	-0.06	-1.15	0.25	0.51
	rostral anterior cingulate	-0.08	-1.55	0.12	0.43	-0.09	-1.67	0.095	0.29
	rostral middle frontal	-0.07	-1.25	0.21	0.46	-0.12	-2.22	0.03	0.15
	superior frontal	-0.08	-1.54	0.12	0.43	-0.06	-1.08	0.28	0.55
	superior parietal	-0.0007	-0.01	0.99	0.99	-0.05	-0.98	0.33	0.61
	superior temporal	-0.07	-1.31	0.19	0.45	-0.16	-3.05	0.002	**0.048 (=)**
	supramarginal	0.0006	0.01	0.99	0.99	-0.04	-0.77	0.44	0.71
	frontal pole	-0.05	-0.85	0.39	0.68	-0.09	-1.66	0.098	0.29
	temporal pole	-0.03	-0.56	0.58	0.75	0.05	0.94	0.35	0.63
	transverse temporal	-0.07	-1.25	0.21	0.46	-0.07	-1.30	0.19	0.49
	insula	-0.009	-0.17	0.86	0.94	-0.003	-0.06	0.95	0.99
	**Total Surface Area**	-0.12	-2.31	0.02	0.20	-0.16	-3.03	0.003	**0.048 (=)**
Cortical Thickness	banks superior temporal	0.097	1.83	0.07	0.38	0.10	1.96	0.05	0.23
	caudal anterior cingulate	0.08	1.48	0.14	0.43	0.07	1.23	0.22	0.49
	caudal middle frontal	0.02	0.40	0.69	0.83	0.007	0.14	0.89	0.95
	**cuneus**	-0.07	-1.32	0.19	0.45	-0.04	-0.83	0.41	0.69
	entorhinal	-0.07	-1.33	0.19	0.45	-0.02	-0.42	0.67	0.81
	fusiform	-0.01	-0.28	0.78	0.88	-0.02	-0.30	0.76	0.87
	inferior parietal	0.02	0.36	0.72	0.85	0.07	1.31	0.19	0.49
	inferior temporal	0.04	0.75	0.45	0.70	0.099	1.86	0.06	0.25
	isthmus cingulate	-0.04	-0.74	0.46	0.70	-0.096	-1.80	0.07	0.26

	lateral occipital	-0.04	-0.74	0.46	0.70	-0.04	-0.66	0.51	0.72
	lateral orbitofrontal	0.08	1.46	0.15	0.43	0.04	0.70	0.48	0.72
	**lingual**	0.03	0.63	0.53	0.71	-0.002	-0.03	0.97	0.99
	medial orbitofrontal	0.12	2.30	0.02	0.20	0.05	0.87	0.39	0.67
	middle temporal	0.09	1.62	0.11	0.43	0.09	1.66	0.098	0.29
	parahippocampal	-0.08	-1.55	0.12	0.43	-0.04	-0.69	0.49	0.72
	para central	0.002	0.05	0.96	0.99	0.04	0.68	0.50	0.72
	pars opercularis	0.09	1.61	0.11	0.43	0.03	0.48	0.63	0.79
	pars orbitalis	0.06	1.13	0.26	0.54	0.04	0.76	0.44	0.71
	pars triangularis	0.06	1.10	0.27	0.54	0.07	1.27	0.21	0.49
	pericalcarine	-0.07	-1.41	0.16	0.43	-0.04	-0.81	0.42	0.69
	post central	0.02	0.39	0.69	0.83	0.0008	0.02	0.99	0.99
	posterior cingulate	0.02	0.30	0.76	0.87	-0.02	-0.30	0.76	0.87
	pre central	-0.04	-0.66	0.51	0.70	-0.002	-0.04	0.97	0.99
	precuneus	0.04	0.67	0.51	0.70	0.01	0.28	0.78	0.88
	rostral anterior cingulate	0.05	0.89	0.37	0.68	0.03	0.63	0.53	0.73
	rostral middle frontal	0.08	1.43	0.15	0.43	0.01	0.19	0.85	0.93
	superior frontal	0.04	0.70	0.48	0.70	0.0009	0.02	0.99	0.99
	superior parietal	0.05	0.94	0.35	0.65	0.07	1.25	0.21	0.49
	superior temporal	0.04	0.78	0.44	0.70	0.06	1.06	0.29	0.55
	supramarginal	0.04	0.68	0.50	0.70	0.03	0.47	0.64	0.79
	frontal pole	0.11	2.06	0.04	0.26	0.12	2.30	0.02	0.14
	temporal pole	0.05	0.97	0.33	0.63	0.03	0.58	0.56	0.75
	transverse temporal	-0.02	-0.31	0.76	0.87	0.03	0.54	0.59	0.77
	insula	0.07	1.28	0.20	0.46	-0.03	-0.50	0.62	0.79
	**Mean Thickness**	0.04	0.68	0.50	0.70	0.03	0.62	0.54	0.73
Intracranial volume	**ICV**	-0.24	-4.61	5.52E-06	**0.0004 (=)**	-0.17	-3.16	0.002	**0.048 (=)**

Table S12 – Linear regression results after excluding first-degree and second-degree relatives. (=) significant in main analysis and after exclusion; (+) significant after exclusion but not in main analysis; (-) not significant after exclusion but significant in main analysis.

		Associations with PenSZ				Associations with PenDD			
**Measure**	**regions**	**b value**	**t value**	**p value**	**p value (FDR)**	**b value**	**t value**	**p value**	**p value (FDR)**
Volume of subcortical structures	accumbens	-0.01	-0.21	0.83	0.90	-0.009	-0.16	0.87	0.96
	amygdala	-0.02	-0.44	0.66	0.81	-0.05	-0.98	0.33	0.54
	caudate	0.09	1.75	0.08	0.24	0.08	1.56	0.12	0.31
	hippocampus	-0.05	-0.89	0.38	0.56	-0.07	-1.22	0.22	0.48
	pallidum	-0.01	-0.19	0.85	0.90	-0.03	-0.52	0.60	0.77
	putamen	-0.03	-0.54	0.59	0.74	-0.03	-0.63	0.53	0.74
	thalalamus	-0.03	-0.57	0.57	0.73	0.004	0.07	0.94	0.96
Cortical surface area	banks superior temporal	-0.05	-0.94	0.35	0.53	-0.10	-1.93	0.05	0.19
	caudal anterior cingulate	-0.17	-3.26	0.001	**0.02 (=)**	-0.20	-3.79	0.0002	**0.007 (=)**
	caudal middle frontal	-0.09	-1.63	0.10	0.27	-0.06	-1.08	0.28	0.49
	**cuneus**	-0.21	-3.95	9.65E-05	**0.003 (=)**	-0.18	-3.45	0.0006	**0.009 (=)**
	entorhinal	0.02	0.34	0.73	0.84	-0.02	-0.45	0.65	0.82
	fusiform	-0.097	-1.82	0.07	0.24	-0.16	-2.97	0.003	**0.03 (=)**
	inferior parietal	-0.06	-1.16	0.25	0.45	-0.09	-1.74	0.08	0.26
	inferior temporal	-0.0996	-1.87	0.06	0.24	-0.09	-1.68	0.09	0.28
	isthmus cingulate	-0.02	-0.38	0.70	0.83	0.03	0.64	0.52	0.74
	lateral occipital	-0.14	-2.67	0.008	0.06	-0.14	-2.54	0.01	0.08
	lateral orbitofrontal	-0.14	-2.71	0.007	**0.06 (-)**	-0.11	-2.11	0.04	0.15
	**lingual**	-0.23	-4.33	2.00E-05	**0.0008 (=)**	-0.22	-4.20	3.35E-05	**0.003 (=)**
	medial orbitofrontal	-0.17	-3.13	0.002	**0.03 (=)**	-0.15	-2.78	0.006	0.05
	middle temporal	-0.04	-0.81	0.42	0.59	-0.10	-1.93	0.05	0.19
	parahippocampal	-0.08	-1.47	0.14	0.32	-0.07	-1.36	0.17	0.41
	para central	-0.04	-0.82	0.41	0.59	-0.02	-0.41	0.68	0.83
	pars opercularis	-0.11	-2.04	0.04	0.20	-0.07	-1.24	0.22	0.48
	pars orbitalis	-0.09	-1.73	0.08	0.24	-0.14	-2.72	0.007	**0.053 (-)**
	pars triangularis	-0.11	-2.08	0.04	0.20	-0.12	-2.27	0.02	0.11
	pericalcarine	-0.14	-2.67	0.008	**0.06 (-)**	-0.13	-2.37	0.02	0.10
	post central	-0.10	-1.90	0.06	0.24	-0.16	-3.05	0.002	**0.03 (=)**
	posterior cingulate	-0.06	-1.08	0.28	0.46	-0.12	-2.32	0.02	0.10

	pre central	-0.009	-0.18	0.86	0.90	0.004	0.08	0.94	0.96
	precuneus	-0.14	-2.55	0.01	0.08	-0.12	-2.17	0.03	0.13
	rostral anterior cingulate	-0.11	-2.03	0.04	0.20	-0.12	-2.34	0.02	0.10
	rostral middle frontal	-0.098	-1.85	0.07	0.24	-0.14	-2.66	0.008	0.06
	superior frontal	-0.08	-1.44	0.15	0.33	-0.06	-1.15	0.25	0.48
	superior parietal	-0.12	-2.33	0.02	0.13	-0.13	-2.48	0.01	0.08
	superior temporal	-0.15	-2.82	0.005	0.06	-0.19	-3.59	0.0004	**0.009 (=)**
	supramarginal	-0.05	-0.95	0.34	0.53	-0.08	-1.43	0.15	0.39
	frontal pole	-0.07	-1.31	0.19	0.37	-0.09	-1.62	0.11	0.28
	temporal pole	-0.07	-1.30	0.19	0.37	0.008	0.15	0.88	0.96
	transverse temporal	-0.08	-1.43	0.15	0.34	-0.06	-1.21	0.23	0.48
	insula	-0.03	-0.47	0.64	0.79	-0.02	-0.37	0.71	0.85
	**Total Surface Area**	-0.15	-2.80	0.005	0.06	-0.18	-3.47	0.0006	**0.009 (=)**
Cortical Thickness	banks superior temporal	0.05	0.86	0.39	0.58	0.06	1.12	0.26	0.48
	caudal anterior cingulate	0.006	0.11	0.91	0.93	0.02	0.30	0.77	0.89
	caudal middle frontal	0.06	1.08	0.28	0.46	0.04	0.71	0.48	0.71
	**cuneus**	0.02	0.31	0.75	0.85	0.02	0.33	0.74	0.88
	entorhinal	-0.06	-1.13	0.26	0.45	-0.01	-0.27	0.78	0.90
	fusiform	0.04	0.73	0.47	0.64	0.009	0.17	0.87	0.96
	inferior parietal	0.04	0.76	0.45	0.63	0.07	1.34	0.18	0.42
	inferior temporal	0.06	1.15	0.25	0.45	0.11	2.00	0.05	0.18
	isthmus cingulate	-0.03	-0.65	0.51	0.69	-0.05	-1.02	0.31	0.53
	lateral occipital	0.03	0.63	0.53	0.70	0.03	0.59	0.56	0.74
	lateral orbitofrontal	0.07	1.40	0.16	0.34	0.05	0.87	0.39	0.63
	**lingual**	0.11	2.02	0.04	0.20	0.06	1.14	0.26	0.48
	medial orbitofrontal	0.08	1.58	0.11	0.28	0.03	0.60	0.55	0.74
	middle temporal	0.08	1.58	0.11	0.28	0.09	1.63	0.10	0.28
	parahippocampal	-0.03	-0.59	0.56	0.72	-0.01	-0.19	0.85	0.96
	para central	0.02	0.42	0.67	0.81	0.04	0.80	0.42	0.66
	pars opercularis	0.11	1.98	0.05	0.21	0.06	1.08	0.28	0.49
	pars orbitalis	0.096	1.79	0.07	0.24	0.09	1.66	0.099	0.28
	pars triangularis	0.09	1.78	0.08	0.24	0.10	1.90	0.06	0.20
	pericalcarine	-0.005	-0.09	0.93	0.94	0.005	0.096	0.92	0.96
	post central	0.06	1.13	0.26	0.45	0.04	0.68	0.50	0.74
	posterior cingulate	-0.0008	-0.02	0.99	0.99	-0.004	-0.07	0.95	0.96

	pre central	-0.05	-0.95	0.34	0.53	-0.03	-0.56	0.58	0.75
	precuneus	0.09	1.71	0.09	0.24	0.06	1.18	0.24	0.48
	rostral anterior cingulate	-0.02	-0.30	0.76	0.85	-0.03	-0.63	0.53	0.74
	rostral middle frontal	0.11	2.14	0.03	0.20	0.06	1.11	0.27	0.48
	superior frontal	0.07	1.33	0.18	0.37	0.04	0.72	0.47	0.71
	superior parietal	0.09	1.70	0.09	0.24	0.09	1.73	0.08	0.26
	superior temporal	-0.00995	-0.19	0.85	0.90	0.006	0.11	0.91	0.96
	supramarginal	0.05	0.99	0.33	0.53	0.04	0.84	0.40	0.64
	frontal pole	0.08	1.52	0.13	0.30	0.07	1.39	0.17	0.40
	temporal pole	0.01	0.21	0.83	0.90	-0.00098	-0.02	0.99	0.99
	transverse temporal	-0.02	-0.37	0.71	0.83	0.02	0.41	0.68	0.83
	insula	0.09	1.71	0.09	0.24	0.03	0.60	0.55	0.74
	**Mean Thickness**	0.07	1.30	0.20	0.37	0.06	1.16	0.25	0.48
Intracranial volume	**ICV**	-0.25	-4.77	2.68E-06	**0.0002 (=)**	-0.18	-3.43	0.0007	**0.009 (=)**

Table S13 – Linear regression results after excluding A)1q21.1 distal deletion carriers and B) 22q11.2 deletion carriers. (=) significant in main analysis and after exclusion; (+) significant after exclusion but not in main analysis; (-) not significant after exclusion but significant in main analysis.

		**A) excluding 1q21.1 distal deletion carriers**							
		**Associations with PenSZ**				**Associations with PenDD**			
**Measure**	**regions**	**b value**	**t value**	**p value**	**p value (FDR)**	**b value**	**t value**	**p value**	**p value (FDR)**
Volume of subcortical structures	accumbens	0.0007	0.01	0.99	0.998	-0.02	-0.31	0.76	0.91
	amygdala	-0.01	-0.26	0.79	0.91	-0.05	-0.93	0.35	0.61
	caudate	0.07	1.27	0.20	0.50	0.03	0.68	0.50	0.72
	hippocampus	-0.05	-0.92	0.36	0.61	-0.07	-1.33	0.18	0.49
	pallidum	-0.003	-0.06	0.96	0.998	-0.03	-0.64	0.52	0.74
	putamen	-0.05	-0.89	0.37	0.61	-0.06	-1.23	0.22	0.50
	thalalamus	-0.04	-0.68	0.50	0.69	-0.0007	-0.01	0.99	0.999

Cortical surface area	banks superior temporal	0.002	0.04	0.97	0.998	-0.05	-1.01	0.31	0.59
	caudal anterior cingulate	-0.14	-2.68	0.008	**0.15 (-)**	-0.16	-3.10	0.002	**0.055 (-)**
	caudal middle frontal	0.02	0.36	0.72	0.86	0.04	0.68	0.50	0.72
	**cuneus**	-0.17	-3.25	0.001	**0.049 (=)**	-0.13	-2.54	0.01	**0.14 (-)**
	entorhinal	0.05	0.93	0.35	0.61	0.004	0.08	0.94	1.00
	fusiform	-0.05	-0.93	0.35	0.61	-0.13	-2.51	0.01	**0.14 (-)**
	inferior parietal	-0.02	-0.48	0.63	0.82	-0.07	-1.27	0.21	0.49
	inferior temporal	-0.04	-0.80	0.42	0.64	-0.04	-0.73	0.46	0.71
	isthmus cingulate	-0.02	-0.42	0.67	0.85	0.04	0.77	0.44	0.70
	lateral occipital	-0.11	-2.12	0.03	0.23	-0.10	-2.02	0.04	0.29
	lateral orbitofrontal	-0.07	-1.41	0.16	**0.44 (-)**	-0.06	-1.09	0.28	0.59
	**lingual**	-0.18	-3.59	0.0004	**0.03 (=)**	-0.17	-3.41	0.0007	**0.055 (-)**
	medial orbitofrontal	-0.10	-2.00	0.05	**0.26 (-)**	-0.09	-1.68	0.09	0.38
	middle temporal	0.01	0.19	0.85	0.93	-0.05	-0.95	0.34	0.61
	parahippocampal	-0.04	-0.72	0.47	0.67	-0.04	-0.72	0.47	0.71
	para central	-0.008	-0.16	0.87	0.94	-0.003	-0.06	0.95	1.00
	pars opercularis	-0.04	-0.76	0.45	0.66	-0.03	-0.54	0.59	0.78
	pars orbitalis	-0.05	-0.88	0.38	0.61	-0.12	-2.36	0.02	**0.16 (-)**
	pars triangularis	-0.04	-0.86	0.39	0.61	-0.09	-1.73	0.08	0.38
	pericalcarine	-0.12	-2.27	0.02	**0.20 (-)**	-0.096	-1.85	0.07	0.34
	post central	-0.098	-1.90	0.06	0.30	-0.16	-3.15	0.002	**0.055 (-)**
	posterior cingulate	-0.02	-0.39	0.70	0.85	-0.08	-1.48	0.14	0.45
	pre central	0.05	0.99	0.32	0.61	0.04	0.85	0.39	0.64
	precuneus	-0.13	-2.62	0.01	0.15	-0.11	-2.11	0.04	0.25
	rostral anterior cingulate	-0.05	-0.95	0.34	0.61	-0.07	-1.29	0.20	0.49
	rostral middle frontal	-0.02	-0.39	0.70	0.85	-0.07	-1.40	0.16	0.47
	superior frontal	-0.01	-0.27	0.79	0.91	-0.0096	-0.18	0.85	0.97
	superior parietal	-0.13	-2.61	0.01	0.15	-0.15	-2.84	0.00	0.09
	superior temporal	-0.08	-1.55	0.12	0.42	-0.14	-2.70	0.007	**0.11 (-)**
	supramarginal	-0.01	-0.24	0.81	0.92	-0.05	-0.95	0.34	0.61
	frontal pole	-0.04	-0.84	0.40	0.61	-0.08	-1.48	0.14	0.45
	temporal pole	-0.07	-1.31	0.19	0.49	0.007	0.14	0.89	0.99
	transverse temporal	-0.0002	-0.003	1.00	0.998	-0.01	-0.23	0.82	0.95
	insula	0.03	0.59	0.56	0.75	0.02	0.43	0.67	0.86
	**Total Surface Area**	-0.08	-1.50	0.13	0.42	-0.13	-2.46	0.01	**0.14 (-)**

Cortical Thickness	banks superior temporal	0.07	1.30	0.19	0.49	0.08	1.59	0.11	0.42
	caudal anterior cingulate	-0.03	-0.57	0.57	0.75	-0.02	-0.41	0.68	0.86
	caudal middle frontal	0.02	0.32	0.75	0.88	0.001	0.02	0.98	0.999
	**cuneus**	0.06	1.25	0.21	0.50	0.05	0.90	0.37	0.62
	entorhinal	-0.05	-0.93	0.35	0.61	-0.009	-0.18	0.86	0.97
	fusiform	0.07	1.43	0.15	0.44	0.05	0.88	0.38	0.63
	inferior parietal	0.06	1.13	0.26	0.57	0.09	1.69	0.09	0.38
	inferior temporal	0.08	1.52	0.13	0.42	0.12	2.32	0.02	0.16
	isthmus cingulate	-0.04	-0.86	0.39	0.61	-0.08	-1.54	0.12	0.44
	lateral occipital	0.08	1.51	0.13	0.42	0.06	1.09	0.28	0.59
	lateral orbitofrontal	0.08	1.60	0.11	0.42	0.06	1.06	0.29	0.59
	**lingual**	0.11	2.06	0.04	0.24	0.05	1.01	0.31	0.59
	medial orbitofrontal	0.05	1.05	0.29	0.59	-0.0004	-0.007	0.99	0.999
	middle temporal	0.096	1.87	0.06	0.30	0.09	1.65	0.10	0.39
	parahippocampal	-0.01	-0.22	0.82	0.92	-3.49E-05	-0.0007	0.999	0.999
	para central	0.06	1.08	0.28	0.58	0.07	1.37	0.17	0.48
	pars opercularis	0.13	2.51	0.01	0.16	0.07	1.43	0.15	0.47
	pars orbitalis	0.12	2.37	0.02	0.20	0.10	1.95	0.05	0.30
	pars triangularis	0.08	1.64	0.10	0.42	0.09	1.76	0.08	0.38
	pericalcarine	0.06	1.10	0.27	0.57	0.05	0.99	0.32	0.60
	post central	0.04	0.73	0.47	0.67	0.02	0.30	0.77	0.91
	posterior cingulate	-0.005	-0.10	0.92	0.98	-0.03	-0.58	0.56	0.78
	pre central	-0.06	-1.19	0.23	0.54	-0.03	-0.56	0.58	0.78
	precuneus	0.09	1.77	0.08	0.35	0.05	1.02	0.31	0.59
	rostral anterior cingulate	-0.02	-0.43	0.67	0.85	-0.03	-0.53	0.59	0.78
	rostral middle frontal	0.12	2.31	0.02	0.20	0.06	1.06	0.29	0.59
	superior frontal	0.06	1.10	0.27	0.57	0.02	0.33	0.74	0.90
	superior parietal	0.11	2.11	0.04	0.23	0.0998	1.93	0.05	0.30
	superior temporal	-0.03	-0.59	0.55	0.75	-0.0001	-0.003	0.998	0.999
	supramarginal	0.05	1.03	0.30	0.59	0.04	0.75	0.45	0.71
	frontal pole	0.07	1.45	0.15	0.44	0.07	1.42	0.16	0.47
	temporal pole	0.0008	0.02	0.99	1.00	-0.0032	-0.06	0.95	0.999
	transverse temporal	-0.07	-1.32	0.19	0.49	-0.02	-0.39	0.70	0.87
	insula	0.09	1.73	0.08	0.37	0.03	0.50	0.61	0.80

	**Mean Thickness**	0.08	1.58	0.12	0.42	0.07	1.29	0.20	0.49
Intracranial volume	**ICV**	-0.11	-2.24	0.03	**0.20 (-)**	-0.07	-1.29	0.20	**0.49 (-)**

		**B) excluding 22q11.2 deletion carriers**							
		**Associations with PenSZ**				**Associations with PenDD**			
**Measure**	**regions**	**b value**	**t value**	**p value**	**p value (FDR)**	**b value**	**t value**	**p value**	**p value (FDR)**
Volume of subcortical structures	accumbens	-0.07	-1.40	0.16	0.42	-0.08	-1.64	0.10	0.39
	amygdala	-0.01	-0.25	0.80	0.89	-0.05	-0.96	0.34	0.61
	caudate	0.005	0.09	0.93	0.97	-0.03	-0.65	0.51	0.75
	hippocampus	-0.03	-0.58	0.56	0.77	-0.05	-1.05	0.29	0.57
	pallidum	-0.03	-0.50	0.62	0.77	-0.06	-1.16	0.25	0.53
	putamen	-0.01	-0.26	0.79	0.89	-0.03	-0.65	0.52	0.75
	thalalamus	-0.03	-0.63	0.53	0.77	0.005	0.097	0.92	0.92
Cortical surface area	banks superior temporal	-0.03	-0.51	0.61	0.77	-0.08	-1.52	0.13	0.40
	caudal anterior cingulate	-0.14	-2.69	0.007	**0.097 (-)**	-0.15	-3.03	0.003	**0.052 (-)**
	caudal middle frontal	-0.09	-1.81	0.07	0.29	-0.05	-0.92	0.36	0.63
	**cuneus**	-0.17	-3.42	0.0007	**0.03 (=)**	-0.13	-2.47	0.01	**0.12 (-)**
	entorhinal	0.01	0.29	0.77	0.88	-0.03	-0.55	0.58	0.76
	fusiform	-0.05	-1.01	0.31	0.65	-0.14	-2.65	0.008	**0.11 (-)**
	inferior parietal	-0.06	-1.10	0.27	0.59	-0.096	-1.88	0.06	0.30
	inferior temporal	-0.12	-2.43	0.02	0.17	-0.10	-2.01	0.05	0.25
	isthmus cingulate	-0.005	-0.10	0.92	0.97	0.06	1.18	0.24	0.53
	lateral occipital	-0.07	-1.42	0.16	0.42	-0.07	-1.28	0.20	0.52
	lateral orbitofrontal	-0.16	-3.24	0.001	**0.03 (=)**	-0.13	-2.51	0.01	0.12
	**lingual**	-0.14	-2.77	0.006	**0.09 (-)**	-0.13	-2.58	0.01	**0.12 (-)**
	medial orbitofrontal	-0.15	-2.88	0.004	**0.08 (-)**	-0.12	-2.29	0.02	0.16
	middle temporal	-0.007	-0.13	0.90	0.97	-0.07	-1.29	0.20	0.52
	parahippocampal	-0.04	-0.72	0.47	0.77	-0.03	-0.67	0.50	0.75
	para central	0.02	0.36	0.72	0.88	0.03	0.65	0.51	0.75
	pars opercularis	-0.09	-1.84	0.07	0.29	-0.06	-1.22	0.22	0.53
	pars orbitalis	-0.098	-1.92	0.06	0.27	-0.18	-3.49	0.0005	**0.02 (=)**

	pars triangularis	-0.08	-1.53	0.13	0.39	-0.12	-2.26	0.02	0.16
	pericalcarine	-0.12	-2.37	0.02	**0.18 (-)**	-0.09	-1.76	0.08	0.34
	post central	-0.02	-0.37	0.71	0.88	-0.099	-1.92	0.06	**0.29 (-)**
	posterior cingulate	-0.02	-0.30	0.76	0.88	-0.07	-1.38	0.17	0.47
	pre central	-0.04	-0.71	0.48	0.77	-0.03	-0.51	0.61	0.76
	precuneus	-0.06	-1.23	0.22	0.48	-0.04	-0.79	0.43	0.70
	rostral anterior cingulate	-0.099	-1.94	0.05	0.27	-0.10	-2.04	0.04	0.25
	rostral middle frontal	-0.08	-1.52	0.13	0.39	-0.12	-2.34	0.02	0.15
	superior frontal	-0.08	-1.54	0.12	0.39	-0.06	-1.09	0.28	0.56
	superior parietal	-0.03	-0.59	0.56	0.77	-0.06	-1.08	0.28	0.56
	superior temporal	-0.12	-2.26	0.02	0.19	-0.17	-3.40	0.0007	**0.02 (=)**
	supramarginal	0.002	0.04	0.97	0.97	-0.04	-0.71	0.48	0.74
	frontal pole	-0.04	-0.76	0.45	0.76	-0.07	-1.39	0.17	0.47
	temporal pole	-0.05	-1.01	0.31	0.65	0.03	0.62	0.54	0.75
	transverse temporal	-0.09	-1.75	0.08	0.32	-0.09	-1.66	0.098	0.39
	insula	-0.05	-0.94	0.35	0.68	-0.05	-0.89	0.37	0.63
	**Total Surface Area**	-0.10	-2.01	0.05	0.26	-0.14	-2.82	0.005	0.08
Cortical Thickness	banks superior temporal	0.07	1.37	0.17	0.42	0.08	1.62	0.11	0.39
	caudal anterior cingulate	0.03	0.51	0.61	0.77	0.02	0.38	0.71	0.79
	caudal middle frontal	0.03	0.64	0.52	0.77	0.008	0.16	0.87	0.89
	**cuneus**	-0.002	-0.04	0.97	0.97	-0.01	-0.24	0.81	0.85
	entorhinal	-0.10	-2.04	0.04	0.26	-0.06	-1.12	0.26	0.56
	fusiform	0.01	0.22	0.82	0.90	-0.01	-0.24	0.81	0.85
	inferior parietal	0.03	0.54	0.59	0.77	0.06	1.20	0.23	0.53
	inferior temporal	0.03	0.68	0.50	0.77	0.08	1.60	0.11	0.39
	isthmus cingulate	-0.03	-0.60	0.55	0.77	-0.08	-1.47	0.14	0.43
	lateral occipital	-0.004	-0.09	0.93	0.97	-0.02	-0.34	0.73	0.81
	lateral orbitofrontal	0.07	1.28	0.20	0.46	0.03	0.60	0.55	0.75
	**lingual**	0.11	2.06	0.04	0.26	0.04	0.84	0.40	0.67
	medial orbitofrontal	0.12	2.29	0.02	0.19	0.04	0.75	0.45	0.72
	middle temporal	0.096	1.87	0.06	0.28	0.08	1.56	0.12	0.39
	parahippocampal	-0.05	-0.90	0.37	0.71	-0.02	-0.47	0.64	0.76
	para central	-0.003	-0.06	0.95	0.97	0.02	0.46	0.65	0.76
	pars opercularis	0.09	1.67	0.095	0.35	0.03	0.56	0.58	0.76
	pars orbitalis	0.04	0.78	0.43	0.76	0.02	0.42	0.68	0.79

	pars triangularis	0.08	1.58	0.12	0.39	0.08	1.58	0.11	0.39
	pericalcarine	-0.04	-0.78	0.44	0.76	-0.03	-0.55	0.58	0.76
	post central	0.05	0.94	0.35	0.68	0.02	0.33	0.74	0.81
	posterior cingulate	-0.02	-0.30	0.76	0.88	-0.05	-0.96	0.34	0.61
	pre central	-0.07	-1.30	0.19	0.46	-0.03	-0.63	0.53	0.75
	precuneus	0.07	1.42	0.16	0.42	0.03	0.51	0.61	0.76
	rostral anterior cingulate	0.07	1.37	0.17	0.42	0.05	1.00	0.32	0.60
	rostral middle frontal	0.08	1.57	0.12	0.39	0.006	0.12	0.91	0.92
	superior frontal	0.03	0.53	0.59	0.77	-0.02	-0.38	0.70	0.79
	superior parietal	0.07	1.37	0.17	0.42	0.06	1.24	0.22	0.53
	superior temporal	0.04	0.78	0.44	0.76	0.06	1.17	0.24	0.53
	supramarginal	0.03	0.67	0.50	0.77	0.01	0.24	0.81	0.85
	frontal pole	0.10	1.99	0.05	0.26	0.09	1.81	0.07	0.33
	temporal pole	0.04	0.69	0.49	0.77	0.02	0.40	0.69	0.79
	transverse temporal	-0.02	-0.31	0.76	0.88	0.03	0.53	0.60	0.76
	insula	0.03	0.55	0.58	0.77	-0.05	-0.90	0.37	0.63
	**Mean Thickness**	0.04	0.81	0.42	0.76	0.02	0.48	0.63	0.76
Intracranial volume	**ICV**	-0.26	-5.25	2.46E-07	**1.92E-05 (=)**	-0.17	-3.44	0.0007	**0.02 (=)**

Table S14 – Linear regression results after excluding CNVs with n<3. (=) significant in main analysis and after exclusion; (+) significant after exclusion but not in main analysis; (-) not significant after exclusion but significant in main analysis.

		**Associations with PenSZ**				**Associations with PenDD**			
**Measure**	**regions**	**b value**	**t value**	**p value**	**p value (FDR)**	**b value**	**t value**	**p value**	**p value (FDR)**
Volume of subcortical structures	accumbens	-0.02	-0.40	0.69	0.79	-0.006	-0.11	0.92	0.98
	amygdala	-0.05	-1.04	0.30	0.40	-0.06	-1.06	0.29	0.45
	caudate	0.13	2.55	0.01	**0.04 (+)**	0.096	1.85	0.06	0.20
	hippocampus	-0.08	-1.57	0.12	0.20	-0.07	-1.36	0.17	0.33
	pallidum	-0.003	-0.05	0.96	0.998	-0.002	-0.03	0.97	0.98
	putamen	-0.09	-1.81	0.07	0.15	-0.09	-1.75	0.08	0.20
	thalalamus	-0.02	-0.48	0.63	0.75	0.006	0.11	0.91	0.98
	banks superior temporal	-0.08	-1.57	0.12	0.20	-0.10	-1.94	0.05	0.20

Cortical surface area	caudal anterior cingulate	-0.19	-3.63	0.0003	**0.005 (=)**	-0.18	-3.59	0.0004	**0.01 (=)**
	caudal middle frontal	-0.12	-2.34	0.02	0.05	-0.07	-1.39	0.17	0.33
	**cuneus**	-0.21	-4.07	5.80E-05	**0.002 (=)**	-0.17	-3.32	0.001	**0.02 (=)**
	entorhinal	-0.0096	-0.19	0.85	0.91	-0.03	-0.64	0.52	0.70
	fusiform	-0.10	-1.97	0.05	0.11	-0.16	-3.18	0.002	**0.02 (=)**
	inferior parietal	-0.06	-1.21	0.23	0.32	-0.09	-1.78	0.08	0.20
	inferior temporal	-0.13	-2.46	0.01	**0.04 (+)**	-0.12	-2.35	0.02	0.10
	isthmus cingulate	0.02	0.29	0.77	0.84	0.08	1.48	0.14	0.31
	lateral occipital	-0.14	-2.66	0.008	**0.04 (+)**	-0.13	-2.60	0.0096	0.07
	lateral orbitofrontal	-0.14	-2.80	0.005	**0.03 (=)**	-0.11	-2.14	0.03	0.15
	**lingual**	-0.18	-3.57	0.0004	**0.005 (=)**	-0.18	-3.55	0.0004	**0.01 (=)**
	medial orbitofrontal	-0.15	-2.95	0.003	**0.02 (=)**	-0.13	-2.42	0.02	0.10
	middle temporal	-0.04	-0.80	0.43	0.53	-0.08	-1.58	0.11	0.27
	parahippocampal	-0.05	-0.90	0.37	0.47	-0.04	-0.76	0.45	0.62
	para central	-0.13	-2.47	0.01	**0.04 (+)**	-0.05	-0.97	0.33	0.51
	pars opercularis	-0.11	-2.16	0.03	0.08	-0.07	-1.44	0.15	0.32
	pars orbitalis	-0.09	-1.75	0.08	0.16	-0.16	-3.02	0.003	**0.03 (=)**
	pars triangularis	-0.08	-1.62	0.11	0.20	-0.09	-1.79	0.07	0.20
	pericalcarine	-0.15	-2.95	0.003	**0.02 (=)**	-0.12	-2.30	0.02	0.11
	post central	-0.08	-1.56	0.12	0.20	-0.13	-2.60	0.0097	**0.07 (-)**
	posterior cingulate	-0.09	-1.69	0.09	0.18	-0.11	-2.12	0.03	0.15
	pre central	-0.06	-1.16	0.25	0.35	-0.03	-0.65	0.52	0.70
	precuneus	-0.13	-2.59	0.01	**0.04 (+)**	-0.096	-1.84	0.07	0.20
	rostral anterior cingulate	-0.14	-2.64	0.009	**0.04 (+)**	-0.10	-2.01	0.05	0.19
	rostral middle frontal	-0.11	-2.05	0.04	0.10	-0.14	-2.68	0.008	0.07
	superior frontal	-0.13	-2.43	0.02	**0.045 (+)**	-0.08	-1.45	0.15	0.32
	superior parietal	-0.08	-1.56	0.12	0.20	-0.098	-1.89	0.06	0.20
	superior temporal	-0.10	-1.96	0.05	0.11	-0.14	-2.62	0.009	**0.07 (-)**
	supramarginal	-0.04	-0.72	0.47	0.57	-0.06	-1.20	0.23	0.39
	frontal pole	-0.06	-1.13	0.26	0.36	-0.09	-1.81	0.07	0.20
	temporal pole	-0.08	-1.55	0.12	0.20	0.001	0.02	0.98	0.98
	transverse temporal	-0.08	-1.48	0.14	0.21	-0.06	-1.13	0.26	0.41
	insula	-0.02	-0.33	0.74	0.81	0.03	0.54	0.59	0.74
	**Total Surface Area**	-0.16	-3.06	0.002	**0.02 (+)**	-0.17	-3.31	0.001	**0.02 (=)**
	banks superior temporal	0.10	2.00	0.05	0.11	0.095	1.84	0.07	0.20

Cortical Thickness	caudal anterior cingulate	0.07	1.26	0.21	0.30	0.01	0.27	0.79	0.92
	caudal middle frontal	0.08	1.46	0.15	0.22	0.02	0.36	0.72	0.85
	**cuneus**	-0.0009	-0.02	0.99	0.998	-0.002	-0.04	0.96	0.98
	entorhinal	-0.02	-0.34	0.73	0.81	-0.004	-0.08	0.94	0.98
	fusiform	0.04	0.81	0.42	0.52	0.01	0.23	0.82	0.93
	inferior parietal	0.08	1.50	0.13	0.21	0.06	1.18	0.24	0.39
	inferior temporal	0.13	2.60	0.0096	**0.04 (+)**	0.13	2.49	0.01	0.09
	isthmus cingulate	0.02	0.36	0.72	0.81	-0.04	-0.78	0.44	0.62
	lateral occipital	0.03	0.50	0.62	0.74	0.01	0.22	0.83	0.93
	lateral orbitofrontal	0.17	3.26	0.001	**0.01 (+)**	0.07	1.36	0.17	0.33
	**lingual**	0.095	1.85	0.07	0.14	0.03	0.61	0.54	0.70
	medial orbitofrontal	0.20	3.91	0.0001	**0.003 (+)**	0.09	1.79	0.08	0.20
	middle temporal	0.13	2.59	0.01	**0.04 (+)**	0.09	1.77	0.08	0.20
	parahippocampal	-0.07	-1.43	0.15	0.23	-0.04	-0.82	0.41	0.59
	para central	-0.0001	-0.002	0.998	0.998	0.004	0.08	0.94	0.98
	pars opercularis	0.14	2.65	0.008	**0.04 (+)**	0.05	0.91	0.36	0.55
	pars orbitalis	0.15	2.99	0.003	**0.02 (+)**	0.09	1.77	0.08	0.20
	pars triangularis	0.15	2.98	0.003	**0.02 (+)**	0.098	1.89	0.06	0.20
	pericalcarine	-0.02	-0.43	0.67	0.77	-0.03	-0.62	0.54	0.70
	post central	0.08	1.52	0.13	0.21	0.03	0.56	0.57	0.73
	posterior cingulate	0.08	1.51	0.13	0.21	0.006	0.11	0.91	0.98
	pre central	0.0007	0.01	0.99	0.998	-0.02	-0.48	0.63	0.77
	precuneus	0.13	2.46	0.01	**0.04 (+)**	0.06	1.18	0.24	0.39
	rostral anterior cingulate	0.05	0.97	0.33	0.44	-0.006	-0.11	0.92	0.98
	rostral middle frontal	0.17	3.34	0.0009	**0.01 (+)**	0.07	1.39	0.17	0.33
	superior frontal	0.12	2.34	0.02	0.054	0.03	0.52	0.60	0.74
	superior parietal	0.10	1.96	0.05	0.11	0.08	1.51	0.13	0.30
	superior temporal	0.05	0.93	0.35	0.46	0.04	0.83	0.40	0.59
	supramarginal	0.12	2.28	0.02	0.06	0.06	1.19	0.24	0.39
	frontal pole	0.14	2.68	0.008	**0.04 (+)**	0.12	2.39	0.02	0.095
	temporal pole	0.09	1.72	0.09	0.17	0.07	1.26	0.21	0.38
	transverse temporal	-0.006	-0.12	0.90	0.95	0.02	0.45	0.65	0.78
	insula	0.18	3.61	0.0003	**0.005 (+)**	0.07	1.29	0.20	0.37
	**Mean Thickness**	0.13	2.45	0.01	**0.04 (+)**	0.06	1.24	0.21	0.38

Intracranial volume	**ICV**	-0.25	-4.90	1.46E-06	**0.0001 (=)**	-0.20	-4.00	7.65E-05	**0.006 (=)**

Table S15 – Linear regression results when not correcting for ICV. A) Main sample and B) when excluding 1q21.1 distal deletion. (=) significant in main analysis and after exclusion; (+) significant after exclusion but not in main analysis; (-) not significant after exclusion but significant in main analysis.

		**A) Main sample**							
		**Associations with PenSZ**				**Associations with PenDD**			
**Measure**	**regions**	**b value**	**t value**	**p value**	**p value (FDR)**	**b value**	**t value**	**p value**	**p value (FDR)**
Volume of subcortical structures	accumbens	-0.09	-1.78	0.08	0.11	-0.08	-1.59	0.11	0.18
	amygdala	-0.12	-2.35	0.02	**0.03 (+)**	-0.12	-2.34	0.02	**0.04 (+)**
	caudate	-0.05	-1.03	0.30	0.36	-0.04	-0.85	0.40	0.49
	hippocampus	-0.16	-3.12	0.002	**0.005 (+)**	-0.14	-2.85	0.005	**0.01 (+)**
	pallidum	-0.11	-2.15	0.03	0.05	-0.10	-2.05	0.04	0.08
	putamen	-0.15	-3.02	0.003	**0.006 (+)**	-0.14	-2.71	0.007	**0.02 (+)**
	thalalamus	-0.17	-3.39	0.0008	**0.002 (+)**	-0.10	-2.02	0.04	0.08
Cortical surface area	banks superior temporal	-0.15	-3.04	0.002	**0.006 (+)**	-0.16	-3.10	0.002	**0.007 (+)**
	caudal anterior cingulate	-0.28	-5.73	2.05E-08	**6.14E-07 (=)**	-0.26	-5.23	2.80E-07	**2.18E-05 (=)**
	caudal middle frontal	-0.19	-3.84	0.0001	**0.0005 (+)**	-0.13	-2.52	0.01	**0.03 (+)**
	**cuneus**	-0.28	-5.85	1.04E-08	**6.14E-07 (=)**	-0.22	-4.45	1.12E-05	**0.0002 (=)**
	entorhinal	-0.07	-1.29	0.20	0.25	-0.07	-1.43	0.15	0.23
	fusiform	-0.20	-4.09	5.31E-05	**0.0002 (+)**	-0.22	-4.39	1.46E-05	**0.0002 (=)**
	inferior parietal	-0.19	-3.89	0.0001	**0.0004 (+)**	-0.18	-3.56	0.0004	**0.002 (+)**
	inferior temporal	-0.22	-4.54	7.39E-06	**4.80E-05 (+)**	-0.17	-3.46	0.0006	**0.002 (+)**
	isthmus cingulate	-0.15	-2.98	0.003	**0.007 (+)**	-0.06	-1.25	0.21	0.31
	lateral occipital	-0.23	-4.66	4.48E-06	**3.41E-05 (+)**	-0.19	-3.79	0.0002	**0.0009 (+)**
	lateral orbitofrontal	-0.25	-5.16	3.96E-07	**4.42E-06 (=)**	-0.19	-3.82	0.0002	**0.0008 (+)**
	**lingual**	-0.27	-5.65	3.11E-08	**6.14E-07 (=)**	-0.24	-4.85	1.80E-06	**5.93E-05 (=)**
	medial orbitofrontal	-0.27	-5.61	3.93E-08	**6.14E-07 (=)**	-0.21	-4.20	3.35E-05	**0.0003 (+)**
	middle temporal	-0.16	-3.29	0.001	**0.003 (+)**	-0.16	-3.16	0.002	**0.006 (+)**
	parahippocampal	-0.16	-3.27	0.001	**0.003 (+)**	-0.13	-2.54	0.01	**0.03 (+)**

	para central	-0.17	-3.35	0.0009	**0.003 (+)**	-0.12	-2.40	0.02	**0.04 (+)**
	pars opercularis	-0.20	-4.14	4.24E-05	**0.0002 (+)**	-0.15	-3.05	0.002	**0.008 (+)**
	pars orbitalis	-0.19	-3.87	0.0001	**0.0005 (+)**	-0.21	-4.21	3.13E-05	**0.0003 (=)**
	pars triangularis	-0.19	-3.89	0.0001	**0.0004 (+)**	-0.19	-3.84	0.0001	**0.0008 (+)**
	pericalcarine	-0.22	-4.49	9.62E-06	**5.36E-05 (=)**	-0.18	-3.51	0.0005	**0.002 (+)**
	post central	-0.21	-4.16	4.00E-05	**0.0002 (+)**	-0.22	-4.36	1.70E-05	**0.0002 (=)**
	posterior cingulate	-0.19	-3.73	0.0002	**0.0007 (+)**	-0.19	-3.73	0.0002	**0.001 (+)**
	pre central	-0.17	-3.33	0.00096	**0.003 (+)**	-0.12	-2.31	0.02	**0.04 (+)**
	precuneus	-0.26	-5.34	1.60E-07	**2.08E-06 (+)**	-0.20	-3.95	9.21E-05	**0.0007 (+)**
	rostral anterior cingulate	-0.23	-4.64	4.81E-06	**3.41E-05 (+)**	-0.19	-3.81	0.0002	**0.0008 (+)**
	rostral middle frontal	-0.22	-4.49	9.51E-06	**5.36E-05 (+)**	-0.20	-4.11	4.75E-05	**0.0004 (+)**
	superior frontal	-0.22	-4.47	1.04E-05	**5.42E-05 (+)**	-0.16	-3.23	0.001	**0.005 (+)**
	superior parietal	-0.22	-4.42	1.27E-05	**6.19E-05 (+)**	-0.19	-3.91	0.0001	**0.0007 (+)**
	superior temporal	-0.24	-4.92	1.31E-06	**1.13E-05 (+)**	-0.23	-4.65	4.63E-06	**9.02E-05 (=)**
	supramarginal	-0.17	-3.35	0.0009	**0.003 (+)**	-0.15	-2.97	0.003	**0.009 (+)**
	frontal pole	-0.14	-2.75	0.006	**0.01 (+)**	-0.14	-2.82	0.005	**0.01 (+)**
	temporal pole	-0.14	-2.76	0.006	**0.01 (+)**	-0.05	-1.06	0.29	0.38
	transverse temporal	-0.16	-3.26	0.001	**0.003 (+)**	-0.13	-2.59	0.0099	**0.03 (+)**
	insula	-0.16	-3.31	0.001	**0.003 (+)**	-0.12	-2.38	0.02	**0.04 (+)**
	**Total Surface Area**	-0.27	-5.65	3.16E-08	**6.14E-07 (+)**	-0.24	-4.80	2.28E-06	**5.93E-05 (=)**
Cortical Thickness	banks superior temporal	0.08	1.55	0.12	0.17	0.09	1.80	0.07	0.13
	caudal anterior cingulate	0.07	1.40	0.16	0.22	0.05	1.06	0.29	0.38
	caudal middle frontal	0.03	0.63	0.53	0.59	0.02	0.30	0.76	0.81
	**cuneus**	0.01	0.29	0.77	0.80	0.01	0.22	0.83	0.86
	entorhinal	-0.05	-0.98	0.33	0.38	-0.02	-0.31	0.76	0.81
	fusiform	0.06	1.20	0.23	0.28	0.04	0.75	0.45	0.54
	inferior parietal	0.06	1.17	0.24	0.29	0.09	1.70	0.09	0.15
	inferior temporal	0.095	1.90	0.06	0.09	0.13	2.58	0.01	**0.03 (+)**
	isthmus cingulate	0.004	0.09	0.93	0.93	-0.04	-0.80	0.42	0.51
	lateral occipital	0.03	0.58	0.56	0.61	0.02	0.44	0.66	0.74
	lateral orbitofrontal	0.13	2.49	0.01	**0.02 (+)**	0.09	1.80	0.07	0.13
	**lingual**	0.11	2.18	0.03	**0.0497 (+)**	0.06	1.22	0.22	0.32
	medial orbitofrontal	0.15	3.09	0.002	**0.005 (+)**	0.08	1.64	0.10	0.16
	middle temporal	0.12	2.43	0.02	**0.03 (+)**	0.11	2.13	0.03	0.07
	parahippocampal	-0.02	-0.47	0.64	0.68	-0.01	-0.24	0.81	0.85

	para central	0.008	0.16	0.88	0.89	0.03	0.67	0.51	0.58
	pars opercularis	0.10	2.01	0.05	0.07	0.06	1.18	0.24	0.33
	pars orbitalis	0.13	2.59	0.00995	**0.02 (+)**	0.11	2.17	0.03	0.06
	pars triangularis	0.12	2.41	0.02	**0.03 (+)**	0.12	2.39	0.02	**0.04 (+)**
	pericalcarine	-0.02	-0.42	0.67	0.71	-0.009	-0.17	0.87	0.89
	post central	0.03	0.64	0.52	0.59	0.02	0.34	0.74	0.81
	posterior cingulate	0.03	0.60	0.55	0.60	-0.0002	-0.004	0.997	0.997
	pre central	-0.07	-1.35	0.18	0.23	-0.04	-0.72	0.47	0.55
	precuneus	0.09	1.86	0.06	0.10	0.06	1.20	0.23	0.33
	rostral anterior cingulate	0.07	1.36	0.17	0.23	0.04	0.81	0.42	0.51
	rostral middle frontal	0.15	2.91	0.004	**0.008 (+)**	0.08	1.64	0.10	0.16
	superior frontal	0.09	1.72	0.09	0.12	0.04	0.87	0.39	0.49
	superior parietal	0.08	1.65	0.10	0.14	0.08	1.65	0.10	0.16
	superior temporal	0.01	0.20	0.84	0.86	0.03	0.59	0.56	0.63
	supramarginal	0.08	1.53	0.13	0.17	0.06	1.19	0.23	0.33
	frontal pole	0.12	2.49	0.01	**0.02 (+)**	0.11	2.25	0.03	0.0504
	temporal pole	0.06	1.20	0.23	0.28	0.04	0.85	0.40	0.49
	transverse temporal	-0.04	-0.78	0.44	0.50	-0.002	-0.04	0.97	0.98
	insula	0.10	2.02	0.04	0.07	0.04	0.85	0.40	0.49
	**Mean Thickness**	0.09	1.75	0.08	0.12	0.07	1.47	0.14	0.22
Intracranial volume	**ICV**	-0.24	-5.01	8.10E-07	**7.89E-06 (=)**	-0.18	-3.56	0.0004	**0.002 (=)**

		**B) When excluding 1q21.1 distal deletion**							
		**Associations with PenSZ**				**Associations with PenDD**			
**Measure**	**regions**	**b value**	**t value**	**p value**	**p value (FDR)**	**b value**	**t value**	**p value**	**p value (FDR)**
Volume of subcortical structures	accumbens	-0.03	-0.60	0.55	0.63	-0.03	-0.64	0.52	0.70
	amygdala	-0.05	-1.06	0.29	0.41	-0.07	-1.31	0.19	0.39
	caudate	-2.28E-05	-0.0004	0.9996	0.9996	-0.002	-0.04	0.97	0.97
	hippocampus	-0.09	-1.76	0.08	0.21	-0.09	-1.75	0.08	0.26
	pallidum	-0.05	-0.98	0.33	0.43	-0.06	-1.12	0.26	0.47
	putamen	-0.09	-1.72	0.09	0.21	-0.08	-1.64	0.10	0.30
	thalalamus	-0.09	-1.82	0.07	0.21	-0.04	-0.68	0.50	0.69
	banks superior temporal	-0.05	-1.01	0.31	0.42	-0.08	-1.51	0.13	0.33

Cortical surface area	caudal anterior cingulate	-0.18	-3.62	0.0003	**0.006 (=)**	-0.18	-3.52	0.0005	**0.02 (=)**
	caudal middle frontal	-0.04	-0.83	0.41	0.50	-0.004	-0.08	0.93	0.97
	**cuneus**	-0.20	-3.94	9.90E-05	**0.004 (=)**	-0.15	-2.83	0.005	**0.07 (-)**
	entorhinal	0.004	0.08	0.94	0.99	-0.02	-0.38	0.70	0.86
	fusiform	-0.10	-1.97	0.05	0.19	-0.14	-2.73	0.007	**0.07 (-)**
	inferior parietal	-0.09	-1.77	0.08	0.21	-0.097	-1.87	0.06	0.26
	inferior temporal	-0.098	-1.91	0.06	0.19	-0.07	-1.34	0.18	0.39
	isthmus cingulate	-0.08	-1.63	0.10	0.21	-0.004	-0.07	0.94	0.97
	lateral occipital	-0.15	-2.94	0.004	**0.04 (+)**	-0.12	-2.36	0.02	0.12
	lateral orbitofrontal	-0.13	-2.44	0.02	**0.09 (-)**	-0.08	-1.61	0.11	0.30
	**lingual**	-0.21	-4.14	4.24E-05	**0.003 (=)**	-0.18	-3.56	0.0004	**0.02 (=)**
	medial orbitofrontal	-0.16	-3.04	0.003	**0.03 (=)**	-0.11	-2.11	0.04	0.19
	middle temporal	-0.06	-1.17	0.24	0.35	-0.08	-1.49	0.14	0.33
	parahippocampal	-0.09	-1.70	0.09	0.21	-0.06	-1.24	0.22	0.41
	para central	-0.07	-1.29	0.20	0.31	-0.04	-0.71	0.48	0.67
	pars opercularis	-0.09	-1.66	0.097	0.21	-0.05	-1.04	0.30	0.52
	pars orbitalis	-0.09	-1.83	0.07	0.21	-0.13	-2.61	0.009	**0.09 (-)**
	pars triangularis	-0.09	-1.71	0.09	0.21	-0.11	-2.13	0.03	0.19
	pericalcarine	-0.15	-2.87	0.004	**0.04 (=)**	-0.11	-2.15	0.03	0.19
	post central	-0.14	-2.80	0.005	**0.046 (+)**	-0.17	-3.25	0.001	**0.03 (=)**
	posterior cingulate	-0.08	-1.55	0.12	0.23	-0.10	-2.01	0.05	0.21
	pre central	-0.03	-0.60	0.55	0.63	-0.006	-0.12	0.90	0.97
	precuneus	-0.19	-3.70	0.0003	**0.006 (+)**	-0.13	-2.54	0.01	0.10
	rostral anterior cingulate	-0.11	-2.11	0.04	0.16	-0.09	-1.78	0.08	0.26
	rostral middle frontal	-0.09	-1.71	0.09	0.21	-0.099	-1.93	0.05	0.24
	superior frontal	-0.09	-1.69	0.09	0.21	-0.05	-0.98	0.33	0.53
	superior parietal	-0.17	-3.33	0.0009	**0.01 (+)**	-0.15	-2.96	0.003	0.06
	superior temporal	-0.13	-2.57	0.01	0.08	-0.14	-2.78	0.006	**0.07 (-)**
	supramarginal	-0.08	-1.50	0.14	0.24	-0.08	-1.49	0.14	0.33
	frontal pole	-0.07	-1.44	0.15	0.26	-0.09	-1.77	0.08	0.26
	temporal pole	-0.099	-1.92	0.06	0.19	-0.01	-0.26	0.79	0.94
	transverse temporal	-0.05	-0.95	0.34	0.43	-0.04	-0.75	0.46	0.66
	insula	-0.05	-0.95	0.34	0.43	-0.03	-0.48	0.63	0.81
	**Total Surface Area**	-0.14	-2.77	0.006	**0.046 (+)**	-0.13	-2.51	0.01	**0.098 (-)**
	banks superior temporal	0.06	1.22	0.22	0.34	0.08	1.54	0.13	0.33

Cortical Thickness	caudal anterior cingulate	-0.01	-0.20	0.84	0.90	-0.01	-0.20	0.84	0.97
	caudal middle frontal	0.01	0.22	0.82	0.90	-0.002	-0.04	0.97	0.97
	**cuneus**	0.06	1.11	0.27	0.38	0.04	0.82	0.41	0.62
	entorhinal	-0.04	-0.82	0.41	0.50	-0.006	-0.12	0.91	0.97
	fusiform	0.08	1.54	0.12	0.23	0.05	0.95	0.34	0.54
	inferior parietal	0.06	1.11	0.27	0.38	0.09	1.68	0.09	0.28
	inferior temporal	0.08	1.63	0.10	0.21	0.12	2.37	0.02	0.12
	isthmus cingulate	-0.03	-0.55	0.58	0.66	-0.07	-1.35	0.18	0.39
	lateral occipital	0.07	1.40	0.16	0.27	0.05	1.03	0.30	0.52
	lateral orbitofrontal	0.09	1.80	0.07	0.21	0.06	1.18	0.24	0.45
	**lingual**	0.10	2.01	0.05	0.19	0.05	0.98	0.33	0.53
	medial orbitofrontal	0.07	1.35	0.18	0.29	0.008	0.16	0.87	0.97
	middle temporal	0.10	1.94	0.05	0.19	0.09	1.69	0.09	0.28
	parahippocampal	-0.002	-0.04	0.97	0.9996	0.005	0.10	0.92	0.97
	para central	0.05	0.92	0.36	0.44	0.07	1.28	0.20	0.39
	pars opercularis	0.12	2.39	0.02	0.09	0.07	1.37	0.17	0.39
	pars orbitalis	0.13	2.53	0.01	0.08	0.11	2.04	0.04	0.21
	pars triangularis	0.09	1.68	0.09	0.21	0.09	1.78	0.08	0.26
	pericalcarine	0.05	0.97	0.33	0.43	0.05	0.91	0.36	0.55
	post central	0.02	0.44	0.66	0.74	0.008	0.15	0.88	0.97
	posterior cingulate	-0.0006	-0.01	0.99	0.9996	-0.03	-0.53	0.60	0.79
	pre central	-0.07	-1.33	0.18	0.29	-0.03	-0.64	0.52	0.70
	precuneus	0.09	1.65	0.10	0.21	0.05	0.95	0.34	0.54
	rostral anterior cingulate	8.44E-05	0.002	1.00	0.9996	-0.01	-0.28	0.78	0.93
	rostral middle frontal	0.12	2.42	0.02	0.09	0.06	1.12	0.26	0.47
	superior frontal	0.07	1.27	0.21	0.32	0.02	0.43	0.67	0.83
	superior parietal	0.0996	1.94	0.05	0.19	0.09	1.83	0.07	0.26
	superior temporal	-0.04	-0.70	0.49	0.58	-0.003	-0.06	0.95	0.97
	supramarginal	0.05	1.01	0.31	0.42	0.04	0.74	0.46	0.66
	frontal pole	0.08	1.59	0.11	0.22	0.08	1.50	0.13	0.33
	temporal pole	0.01	0.19	0.85	0.90	0.002	0.04	0.97	0.97
	transverse temporal	-0.07	-1.44	0.15	0.26	-0.02	-0.46	0.65	0.82
	insula	0.09	1.70	0.09	0.21	0.03	0.49	0.63	0.81
	**Mean Thickness**	0.08	1.57	0.12	0.22	0.07	1.28	0.20	0.39

Intracranial volume	**ICV**	-0.11	-2.24	0.03	**0.13 (-)**	-0.07	-1.29	0.20	**0.39 (-)**

Table S16 – Effects of age on brain measures, while correcting for scanner site (using ComBat), sex and ICV.

		Linear association				Quadratic association			
**Measure**	**regions**	**b value**	**t value**	**p value**	**p value (FDR)**	**b value**	**t value**	**p value**	**p value (FDR)**
Volume of subcortical structures	accumbens	-53.53	-71.32	<2e-16	<2e-16	-0.002	-0.002	1.00	0.999
	amygdala	-35.21	-44.38	<2e-16	<2e-16	-3.49	-4.50	7.03E-06	5.48E-05
	caudate	-28.86	-37.32	<2e-16	<2e-16	3.68	4.87	1.13E-06	1.10E-05
	hippocampus	-43.86	-60.86	<2e-16	<2e-16	-6.58	-9.33	1.28E-20	4.99E-19
	pallidum	-41.43	-57.69	<2e-16	<2e-16	3.01	4.28	1.85E-05	1.31E-04
	putamen	-55.21	-86.58	<2e-16	<2e-16	1.01	1.61	0.11	0.23
	thalalamus	-52.11	-96.27	<2e-16	<2e-16	-2.12	-4.00	6.44E-05	0.0004
Cortical surface area	banks superior temporal	-15.44	-18.45	<2e-16	<2e-16	-0.57	-0.71	0.48	0.70
	caudal anterior cingulate	-5.04	-5.62	1.97E-08	2.16E-08	0.31	0.35	0.72	0.89
	caudal middle frontal	-10.80	-13.00	<2e-16	<2e-16	-0.06	-0.07	0.94	0.98
	**cuneus**	-8.05	-9.48	<2e-16	<2e-16	-3.13	-3.81	1.38E-04	0.0008
	entorhinal	3.16	3.33	8.58E-04	8.93E-04	-1.88	-2.05	0.04	0.13
	fusiform	-17.46	-23.81	<2e-16	<2e-16	-0.91	-1.28	0.20	0.36
	inferior parietal	-12.55	-17.00	<2e-16	<2e-16	0.26	0.37	0.71	0.89
	inferior temporal	-15.01	-19.74	<2e-16	<2e-16	-1.25	-1.71	0.09	0.21
	isthmus cingulate	4.21	5.42	6.23E-08	6.75E-08	0.73	0.97	0.33	0.56
	lateral occipital	-10.25	-13.61	<2e-16	<2e-16	-1.22	-1.68	0.09	0.22
	lateral orbitofrontal	-15.34	-20.90	<2e-16	<2e-16	0.08	0.11	0.91	0.97
	**lingual**	-15.66	-18.21	<2e-16	<2e-16	-2.05	-2.47	0.01	0.06
	medial orbitofrontal	-6.23	-8.57	<2e-16	<2e-16	-1.39	-1.98	0.05	0.14
	middle temporal	-18.41	-25.65	<2e-16	<2e-16	-1.03	-1.48	0.14	0.28
	parahippocampal	-18.99	-23.01	<2e-16	<2e-16	-2.23	-2.80	0.005	0.03
	para central	0.46	0.53	0.59	0.59	0.77	0.93	0.35	0.58
	pars opercularis	-16.26	-18.86	<2e-16	<2e-16	0.51	0.61	0.54	0.74
	pars orbitalis	-13.79	-16.89	<2e-16	<2e-16	-0.24	-0.30	0.77	0.91
	pars triangularis	-13.77	-15.58	<2e-16	<2e-16	0.58	0.68	0.50	0.71
	pericalcarine	-5.66	-6.01	1.89E-09	2.11E-09	-2.21	-2.42	0.02	0.06

	post central	-3.05	-4.14	3.56E-05	3.75E-05	0.47	0.66	0.51	0.71
	posterior cingulate	-12.17	-15.32	<2e-16	<2e-16	-0.19	-0.24	0.81	0.91
	pre central	-1.63	-2.22	0.03	0.03	-0.00099	-0.001	0.999	0.999
	precuneus	-12.83	-17.91	<2e-16	<2e-16	-0.07	-0.098	0.92	0.97
	rostral anterior cingulate	-4.35	-5.41	6.44E-08	6.88E-08	-1.26	-1.63	0.10	0.22
	rostral middle frontal	-12.29	-17.36	<2e-16	<2e-16	0.91	1.33	0.18	0.34
	superior frontal	-14.92	-22.91	<2e-16	<2e-16	0.34	0.54	0.59	0.79
	superior parietal	-13.43	-17.53	<2e-16	<2e-16	-0.21	-0.28	0.78	0.91
	superior temporal	-11.35	-16.35	<2e-16	<2e-16	0.15	0.23	0.82	0.91
	supramarginal	-11.00	-14.84	<2e-16	<2e-16	0.20	0.28	0.78	0.91
	frontal pole	-12.87	-13.40	<2e-16	<2e-16	1.79	1.93	0.05	0.15
	temporal pole	-1.36	-1.40	0.16	0.16	-0.39	-0.42	0.68	0.86
	transverse temporal	-6.30	-7.01	2.49E-12	2.82E-12	-1.11	-1.28	0.20	0.36
	insula	8.77	11.90	<2e-16	<2e-16	-1.47	-2.06	0.04	0.13
	**Total Surface Area**	-14.39	-28.69	<2e-16	<2e-16	-0.22	-0.45	0.65	0.86
Cortical Thickness	banks superior temporal	-64.22	-82.03	<2e-16	<2e-16	0.87	1.15	0.25	0.43
	caudal anterior cingulate	-30.33	-30.42	<2e-16	<2e-16	7.61	7.89	3.34E-15	8.04E-14
	caudal middle frontal	-63.87	-81.57	<2e-16	<2e-16	1.30	1.72	0.09	0.21
	**cuneus**	-48.54	-53.48	<2e-16	<2e-16	1.68	1.92	0.06	0.15
	entorhinal	-19.93	-19.08	<2e-16	<2e-16	-2.22	-2.20	0.03	0.10
	fusiform	-62.18	-76.23	<2e-16	<2e-16	1.08	1.37	0.17	0.33
	inferior parietal	-68.20	-91.86	<2e-16	<2e-16	0.07	0.097	0.92	0.97
	inferior temporal	-58.31	-68.74	<2e-16	<2e-16	0.68	0.83	0.40	0.64
	isthmus cingulate	-60.33	-72.14	<2e-16	<2e-16	1.95	2.41	0.02	0.06
	lateral occipital	-54.67	-63.43	<2e-16	<2e-16	-2.02	-2.43	0.02	0.06
	lateral orbitofrontal	-63.54	-78.61	<2e-16	<2e-16	4.21	5.39	7.36E-08	9.57E-07
	**lingual**	-64.04	-81.57	<2e-16	<2e-16	-0.61	-0.81	0.42	0.64
	medial orbitofrontal	-52.25	-57.83	<2e-16	<2e-16	4.53	5.18	2.24E-07	2.50E-06
	middle temporal	-68.47	-91.45	<2e-16	<2e-16	1.35	1.86	0.06	0.16
	parahippocampal	-36.23	-36.72	<2e-16	<2e-16	0.15	0.15	0.88	0.97
	para central	-56.00	-65.85	<2e-16	<2e-16	0.64	0.78	0.44	0.66
	pars opercularis	-67.70	-91.25	<2e-16	<2e-16	3.24	4.51	6.49E-06	5.48E-05
	pars orbitalis	-62.58	-77.86	<2e-16	<2e-16	0.72	0.92	0.36	0.58
	pars triangularis	-69.04	-93.66	<2e-16	<2e-16	1.46	2.05	0.04	0.13
	pericalcarine	-44.58	-47.78	<2e-16	<2e-16	0.001	0.001	0.999	0.999

	post central	-56.33	-67.80	<2e-16	<2e-16	-1.36	-1.69	0.09	0.22
	posterior cingulate	-63.15	-78.38	<2e-16	<2e-16	6.14	7.87	4.12E-15	8.04E-14
	pre central	-63.73	-81.74	<2e-16	<2e-16	-1.23	-1.63	0.10	0.22
	precuneus	-66.87	-89.13	<2e-16	<2e-16	0.99	1.37	0.17	0.33
	rostral anterior cingulate	-42.00	-44.10	<2e-16	<2e-16	8.85	9.61	9.07E-22	7.07E-20
	rostral middle frontal	-65.30	-83.66	<2e-16	<2e-16	0.32	0.42	0.67	0.86
	superior frontal	-72.38	-103.07	<2e-16	<2e-16	1.63	2.40	0.02	0.06
	superior parietal	-55.15	-64.60	<2e-16	<2e-16	-0.68	-0.83	0.41	0.64
	superior temporal	-70.04	-97.80	<2e-16	<2e-16	-0.46	-0.66	0.51	0.71
	supramarginal	-68.79	-93.49	<2e-16	<2e-16	1.13	1.59	0.11	0.23
	frontal pole	-46.34	-49.97	<2e-16	<2e-16	0.23	0.26	0.80	0.91
	temporal pole	-32.49	-32.30	<2e-16	<2e-16	-1.97	-2.02	0.04	0.14
	transverse temporal	-56.56	-66.44	<2e-16	<2e-16	2.27	2.76	0.006	0.03
	insula	-59.17	-71.14	<2e-16	<2e-16	5.29	6.58	5.00E-11	7.79E-10
	**Mean Thickness**	-74.60	-112.34	<2e-16	<2e-16	1.25	1.95	0.05	0.15
Intracranial volume	**ICV**	-16.29	-20.89	<2e-16	<2e-16	0.20	0.26	0.79	0.91

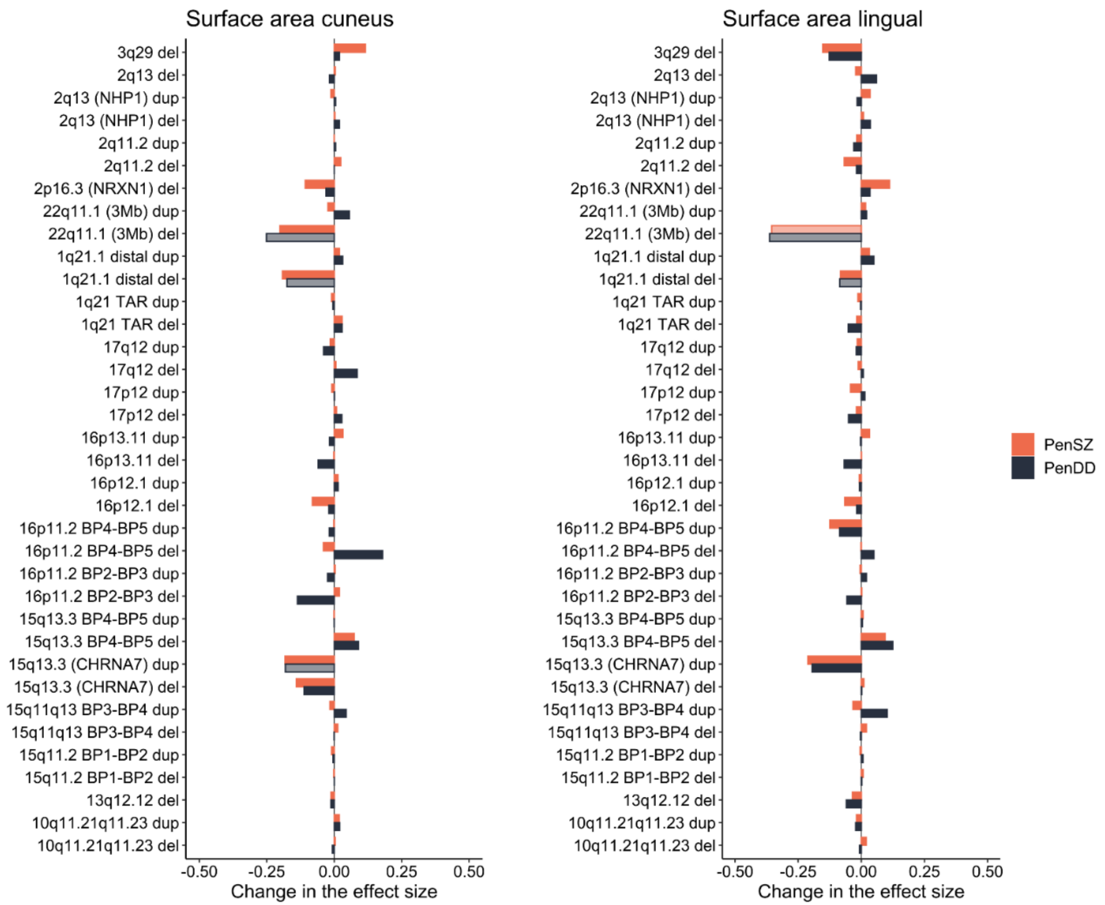

Figure S1 – Effect size change due to each CNV omission from the main analysis in the association between penetrance scores for schizophrenia (PenSZ) and developmental disorders (PenDD) and cortical surface area of the cuneus and lingual gyrus. **Shaded bars** indicate where association was no longer significant after CNV omission. The change in effect size was estimated by calculating the difference between t-values normalised to the square root of the number of participants removed.

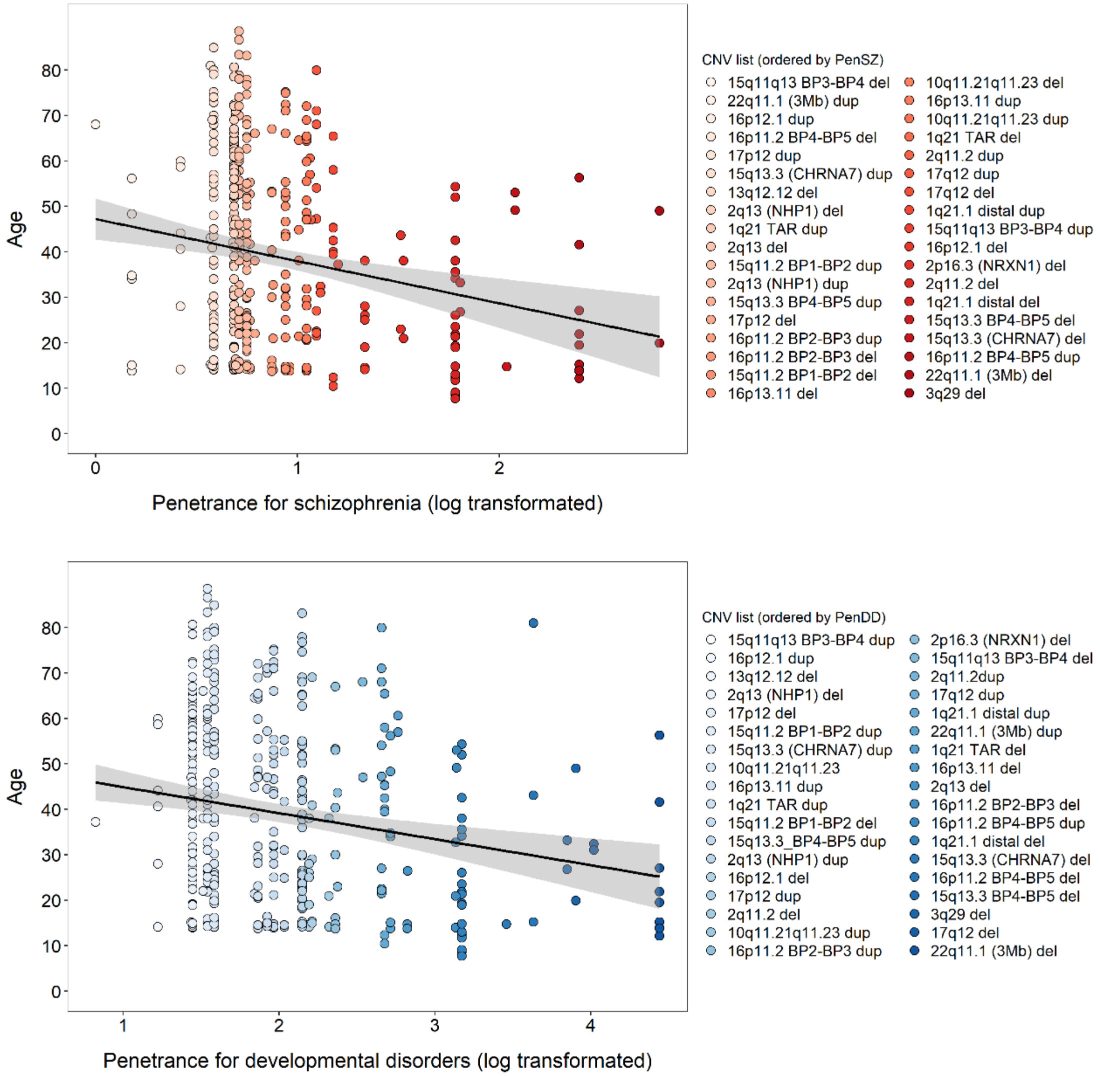

Figure S2 – Scatterplots showing linear associations between logarithmic-transformed CNV penetrance scores and age.
